# Potent Fluorescent Probe for Target‐Engagement Studies of Allosteric Pyruvate Kinase Modulators

**DOI:** 10.1002/anie.202513969

**Published:** 2025-08-29

**Authors:** Oscar Nilsson, Anna P. Valaka, Liliana Håversen, Agnieszka Bogucka, István Köteles, Paul Brear, Mikael Rutberg, Anders Gunnarsson, Marko Hyvönen, Morten Grøtli

**Affiliations:** ^1^ Department of Chemistry and Molecular Biology University of Gothenburg Medicinaregatan 7B Gothenburg SE‐413 90 Sweden; ^2^ Department of Molecular and Clinical Medicine University of Gothenburg and Sahlgrenska University Hospital Gothenburg SE‐413 45 Sweden; ^3^ Department of Biochemistry University of Cambridge 80 Tennis Court Road Cambridge CB2 1GA UK; ^4^ Discovery Sciences R&D Gothenburg AstraZeneca Mölndal SE‐431 83 Sweden

**Keywords:** Allosterism, Biological activity, Drug discovery, Fluorescent probes, Medicinal chemistry

## Abstract

Pyruvate kinases (PKs) are highly allosterically regulated enzymes that play a central role in cellular metabolism and are increasingly recognized as valuable therapeutic targets in cancer, metabolic diseases, and diabetes. Despite their biological and clinical significance, methods to directly assess allosteric ligand engagement of PK isoforms remain limited. Here, we report the development of **LumiPK**, a novel, environment‐sensitive fluorescent tracer designed to monitor allosteric binding to the liver isoform of pyruvate kinase (PKL). **LumiPK** integrates an environment‐sensitive 4‐sulfamonyl‐7‐aminobenzoxadiazole fluorophore into a potent allosteric modulator scaffold. It emerged as the lead compound from a small ligand series, showing high affinity for PKL (*K*_D_  =  37 ± 5 nM) in recombinant assays; the most potent fluorescent PK reporter reported to date. A NanoBRET assay using a PKL‐Nluc fusion (PKL_Nluc_) enabled intracellular monitoring of unlabeled ligand engagement. **LumiPK** maintained high potency (EC_50_  =  18.4 nM) in cellular experiments. Competitive NanoBRET and fluorescence titration assays confirmed binding of known PKL activators (mitapivat, TEPP‐46, DASA‐58) in both cellular and recombinant settings, with *K*_D_ values remaining consistent across these methods. **LumiPK** thus provides a robust tool for probing PKL allosteric modulation and fills a key gap in target engagement technologies for PKL.

## Graphical abstract

**LumiPK**, a novel environment‐sensitive fluorescent tracer, enables direct monitoring of allosteric ligand engagement with pyruvate kinase fused with nanoluciferase (Nluc), focusing on the liver isoform (PKL). In NanoBRET, and fluorescence indicator displacement assays, **LumiPK** shows high affinity and validates known allosteric modulators of PKL. This tool compound advances target engagement technologies and supports broader PK‐related therapeutic research.

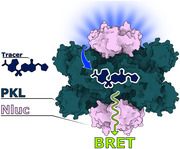

## Introduction

Pyruvate kinases (PKs) are essential enzymes in cellular metabolism, catalyzing the conversion of phosphoenolpyruvate (PEP) to pyruvate while generating adenosine triphosphate (ATP). This reaction, which concludes glycolysis, is critical for maintaining energy homeostasis in the cell.^[^ ^1^ ^]^ PK exists in several isoforms: PKL (predominantly in the liver), PKR (in erythrocytes), PKM1 (in muscle and brain), and PKM2 (in proliferating cells and embryonic tissues), each with distinct regulatory characteristics.^[^ ^2^, ^3^, ^4^ ^]^ Given their central role in energy production, these enzymes are potential targets for therapeutic intervention in conditions where glycolytic activity is altered, including cancer,^[^ ^5^, ^6^, ^7^, ^8^ ^]^ diabetes,^[^ ^9^, ^10^, ^11^, ^12^ ^]^ and various metabolic disorders.^[^ ^13^, ^14^, ^15^, ^16^, ^17^ ^]^

**Figure 1:**
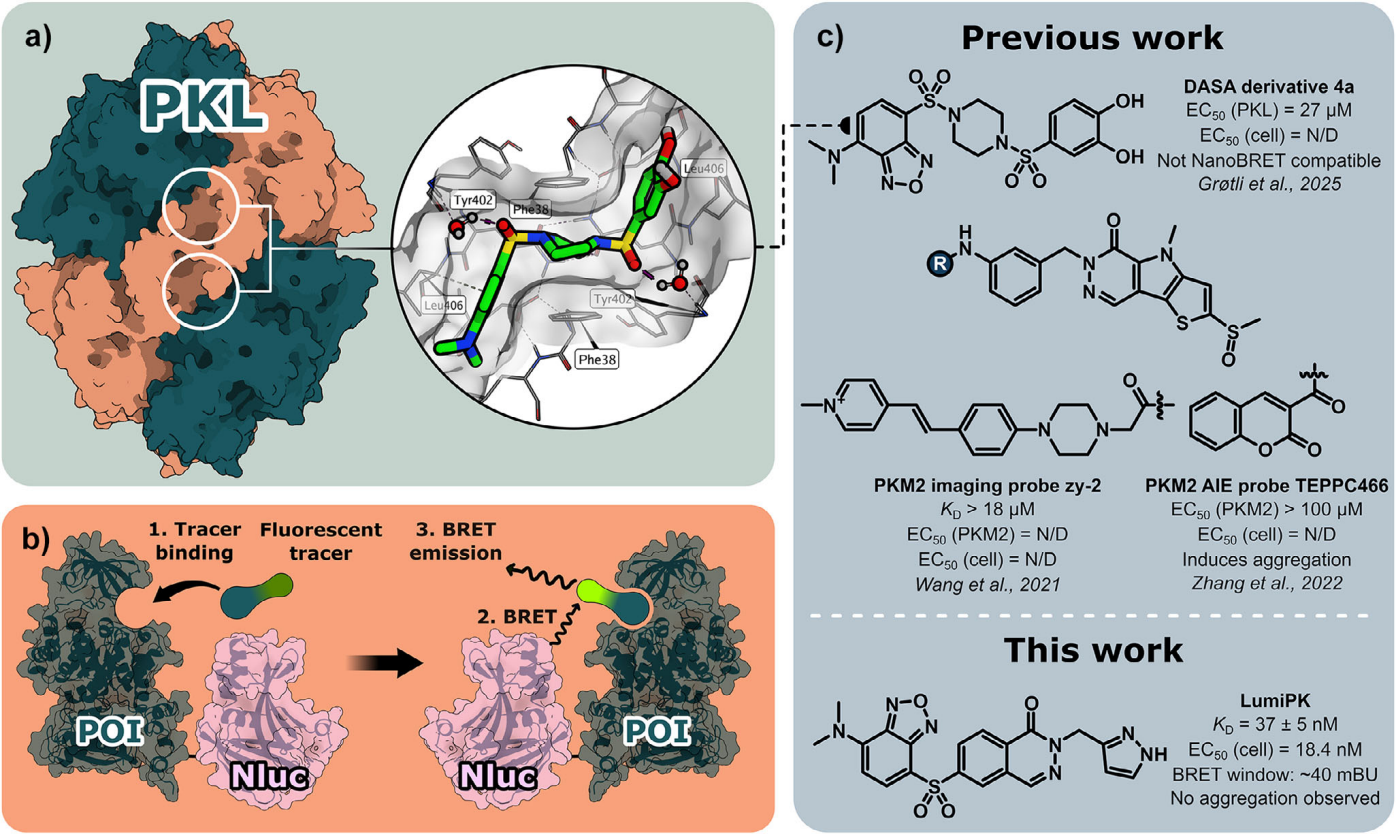
Conceptual summary of PKL structure, NanoBRET principle, and summary of reported fluorescent pyruvate kinase modulators. a) Molecular surface representation of the homotetrameric structure of PKL and locations of the allosteric pockets. Diagonally positioned protein monomers are colored either apricot or petrol. The allosteric pockets are formed at the protomer interfaces and are indicated by white circles. The circular cutout shows the crystal structure of the reported DASA derivative 4a in complex with PKL (PDB ID: 9R3O).^[^ ^43^ ^]^ b) NanoBRET concept: A POI is fused with a Nluc enzyme; 1) a fluorescent tracer binds to the POI; 2) after binding, the tracer is excited through BRET from Nluc; 3) the excited tracer generates a BRET signal with an intensity proportional to pocket occupancy. c) Summary of reported fluorescent allosteric pyruvate kinase modulators, including **LumiPK** (this work). N/D = not determined, AIE = aggregation‐induced emission.

The activity of human PKs is highly modulated by allosteric regulation. Endogenous metabolites, such as fructose 1,6‐bisphosphate (FBP) and amino acids, such as alanine, bind to distinct regulatory sites around the homotetrameric structure and can induce activation or inhibition.^[^ ^16^, ^18^, ^19^, ^20^ ^]^ Additionally, synthetic ligands have been designed to target an allosteric pocket formed at protomer interfaces, enabling control over PK activity (Figure 1a).^[^ ^21^, ^22^, ^23^, ^24^ ^]^ Such ligands have the potential to modulate glycolytic flux inside cells. However, given the multitude of binding sites in PK, their development has been constrained by a lack of cellular target‐engagement assays (TEA) that can selectively report from the allosteric pocket.

TEAs serve as critical tools in pharmacological research by validating the interactions of ligands of interest with targeted proteins. TEAs are especially valuable when used to correlate ligand target engagement with observations of induced phenotypic effects, which is important in validating the mechanism of action (MoA) of drug candidates.^[^ ^25^ ^]^ These assays are also integral in distinguishing on‐target effects from off‐target interactions, a differentiation that is essential for ensuring drug safety.^[^ ^26^ ^]^ Historical examples, such as the cases of iniparib and BIA 10–2474, underscore the risks associated with an incomplete understanding of a compound's MoA, which can lead to serious clinical consequences.^[^ ^27^, ^28^ ^]^

A variety of TEA methodologies exist, including fluorescence resonance energy transfer lifetime imaging microscopy,^[^ ^29^, ^30^ ^]^ bioluminescence resonance energy transfer (BRET),^[^ ^31^, ^32^, ^33^ ^]^ proteolysis targeting chimeras,^[^ ^34^ ^]^ affinity‐based chemical proteomics,^[^ ^30^ ^]^ ligand‐directed protein labeling,^[^ ^30^ ^]^ enzyme fragment complementation assays,^[^ ^30^ ^]^ cellular solvent shift assays,^[^ ^35^ ^]^ and cellular thermal shift assays.^[^ ^30^, ^36^, ^37^ ^]^

Among these, NanoBRET assays are notable for their relative simplicity, high‐throughput compatibility, and low resource requirements, making them prominent in early‐stage research.^[^ ^38^, ^39^ ^]^ As a result, many NanoBRET systems are commercially available for well‐characterized targets such as adenosine kinase, BMX non‐receptor tyrosine kinase, Bruton's tyrosine kinase, cyclin‐dependent kinases 1–19, and death‐associated protein kinase 2.^[^ ^40^ ^]^ However, no NanoBRET systems have been developed for PKL.

Fluorescent ligands are essential for NanoBRET assays, where energy transfer from a nanoluciferase (Nluc)‐tagged protein of interest (POI) to a fluorescent tracer produces a measurable signal upon binding (Figure 1b). Prior to 2025, two instances of fluorescent PKM2 ligands have been reported, both based on the PKM2 activator TEPP‐46 (Figure 1c).^[^ ^41^, ^42^ ^]^ However, because these compounds either induce protein aggregation upon binding,^[^ ^41^ ^]^ or lack the required potency for NanoBRET,^[^ ^42^ ^]^ we designed tracers tailored to allosteric PKL engagement.

We recently reported environment‐sensitive fluorescent diarylsulfonamide (DASA)‐58 derivatives as probes for a fluorescent binding assay targeting recombinant PKL (Figure 1c).^[^ ^43^ ^]^ However, the probes lacked the potency for intracellular applications, making them unsuitable for use in cellular NanoBRET assays.

To address these shortcomings, we developed a novel class of fluorescent phthalazine derivatives as allosteric tracers for PKL. Inspired by modulators described in recent patent literature,^[^ ^44^ ^]^ our design strategy directly incorporated a 4‐sulfamonyl‐7‐aminobenzoxadiazole (SBD) fluorophore into a phthalazine scaffold. Dimethylamine‐substituted SBD is highly environment‐sensitive, with its fluorescence intensity varying according to the polarity of the chemical environment, thereby providing a direct readout of protein occupancy.^[^ ^43^, ^45^ ^]^

Here, we report the development and application of a novel tracer, referred to as **LumiPK** (**IV**), in a functional PKL NanoBRET assay to demonstrate intracellular target engagement of known allosteric PK modulators. This tracer has the potential to accelerate the discovery and optimization of synthetic ligands that modulate PKL activity, and open the door for tracer development targeting the other PK isoforms.

## Results and Discussion

### Design Strategy

The primary drawback of the previously reported DASA‐derived tracers was their low binding affinity for PKL.^[^ ^43^ ^]^ To improve target engagement, we explored alternative molecular scaffolds that align with our design strategy of integrating an SBD fluorophore into the ligand structure.

**Scheme 1:**
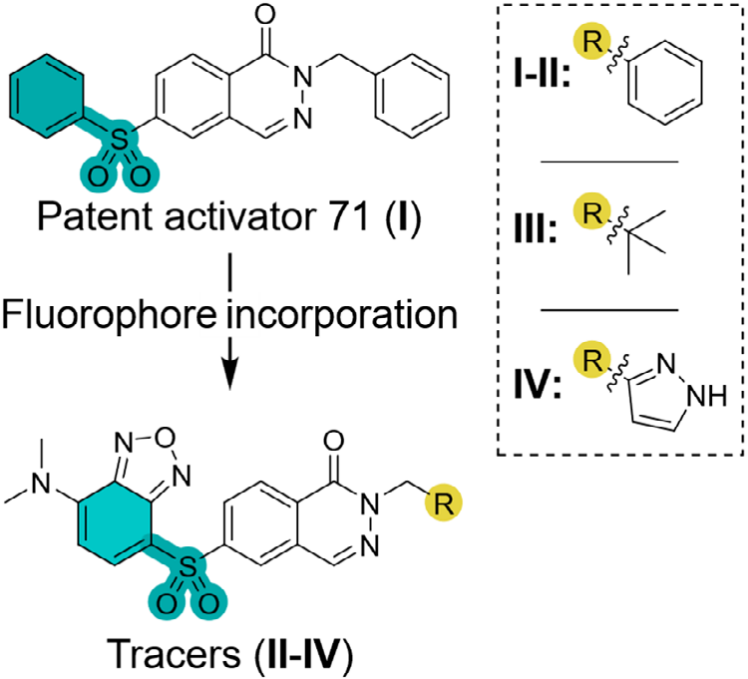
Design strategy for phthalazine‐based fluorescent tracers. The SBD fluorophore was directly incorporated into the phthalazine ligand scaffold. Introduction of benzyl, *tert*‐butyl or 3‐pyrazole R‐groups afforded compounds **I**, **II**, **III** or **IV**, respectively.

Cousin and colleagues previously introduced a class of phthalazine‐derived PKM2 activators containing a sulfone moiety.^[^ ^44^ ^]^ Their study highlighted a broad range of bicyclic aromatic substituents that are well tolerated, yielding several activators with nanomolar potency for both PKM2 and PKL. Drawing inspiration from their approach, we replaced the sulfone‐substituted aromatic group with an SBD fluorophore, aiming to develop a high‐potency tracer (Scheme 1). Starting from patent activator 71 (**I**),^[^ ^44^ ^]^ we incorporated SBD to generate tracer **II**. To explore if the reported structure‐activity relationship (SAR) of the patented activators extends to this new class of tracers, further modifications were made by substituting the benzyl group with a *tert*‐butyl group and a pyrrole group, leading to tracers **III** and **IV**, respectively. Based on the reported data, tracer **IV** (**LumiPK**) was predicted to be the most potent out of the set.^[^ ^44^ ^]^

### Synthesis of Fluorescent Tracers

**Scheme 2:**
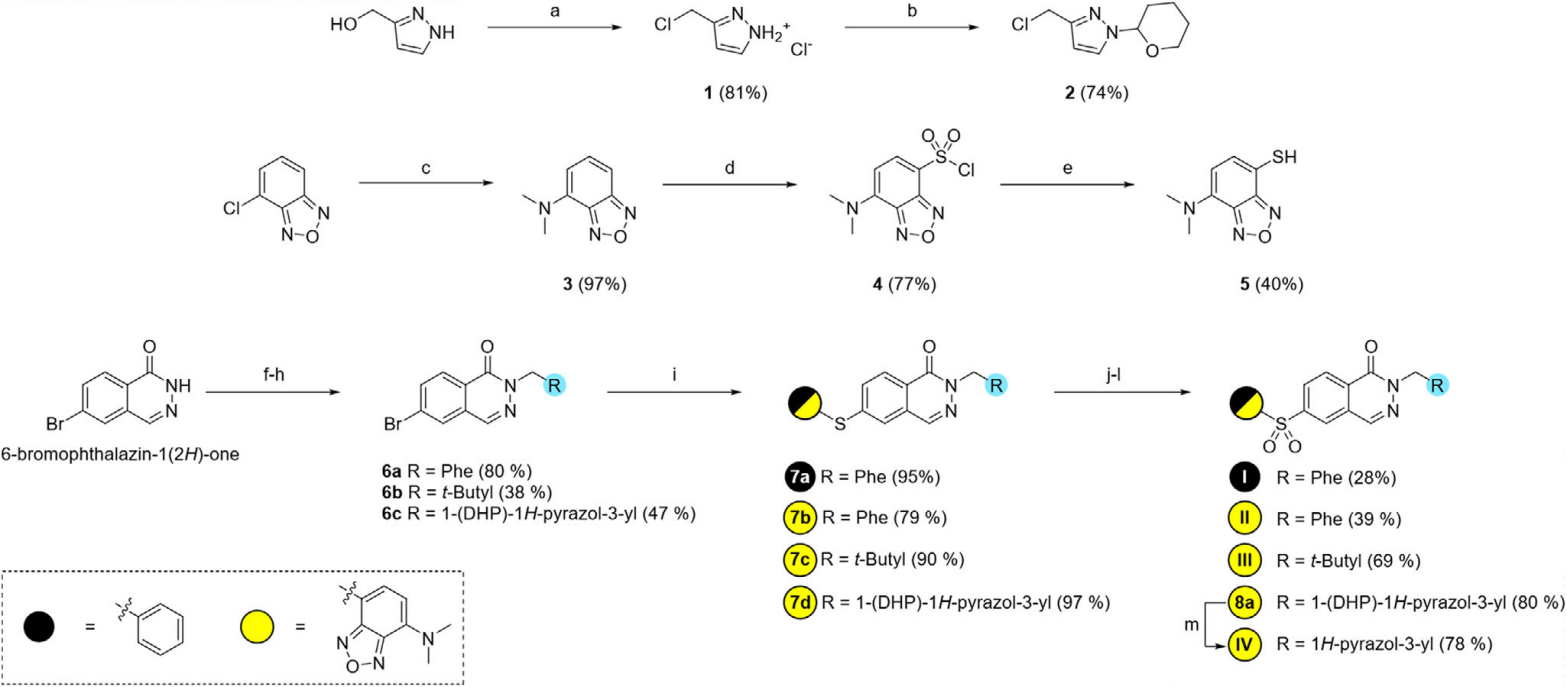
Synthesis of intermediates and phthalazine fluorescent ligands. Isolated yields are reported in parentheses. Reagents and conditions: a) SOCl_2_, room temperature, 2 h. b) 2,3‐Dihydropyran, MeCN, room temperature, 18 h. c) Me_2_NH, 130 °C (MW, 10 bar), 8 h. d) HSO_3_Cl, 100 °C, 20 min. e) SnCl_2_, HCl, 1,4‐dioxane, room temperature, 3 h. f) Neopentyl bromide, KOtBu, DMF, 150 °C, 15 min. g) Benzyl bromide, NaI, NaH, DMF, room temperature, 1 h. h) **2**, NaH, DMF, room temperature, 14 h. i) Thiol, Cs_2_CO_3_, Pd_2_(dba)_3_, Xantphos, DMF, 100 °C, 1–2 h. j) Oxone, DMF, room temperature, 5 h. k) mCPBA, CHCl_3_, 0 °C, 2 h. l) Na_2_WO_4_·2 H_2_O, H_2_O_2_ (30% w/v), DCM/MeOH (1:1), room temperature, 22 h. m) MeSO_4_H, DCM, room temperature, 2 h.

The ligands were synthesized according to a modified version of the previously described procedure (Scheme 2).^[^ ^44^ ^]^ Briefly, 3‐(hydroxymethyl) pyrazole was chlorinated using an excess of thionyl chloride to give **1** as an HCl salt, which was subsequently protected by reacting it with 2,4‐dihydrofuran in MeCN to yield intermediate **2**. 4‐Chloro‐2,1,3‐benzoxadiazole was submitted for nucleophilic aromatic substitution with Me_2_NH at elevated temperature to afford amine **3**. Chlorosulfonation of **3** with an excess of chlorosulfonic acid then gave **4,** which was further reduced with tin(II)chloride to give the thiol intermediate **5**. 6‐Bromophthalazin‐1(2*H*)‐one was reacted with benzyl bromide, neopentyl bromide, or chloride **2** to afford alkylated intermediates **6a**‐**c**, respectively. Intermediates **6a**–**c** were submitted for palladium‐mediated cross‐coupling reactions with either thiophenol or **5** to yield the corresponding thioethers **7a**–**d**. Oxidation of the sulfide groups of **7a**, **7b,** and **7c** under various conditions yielded the final compounds **I**, **II,** and **III,** respectively. Dihydropyran‐protected intermediates **7d** were similarly oxidized and further treated with acid for deprotection, giving tracer **IV**.

### X‐ray Crystallography

**Figure 2:**
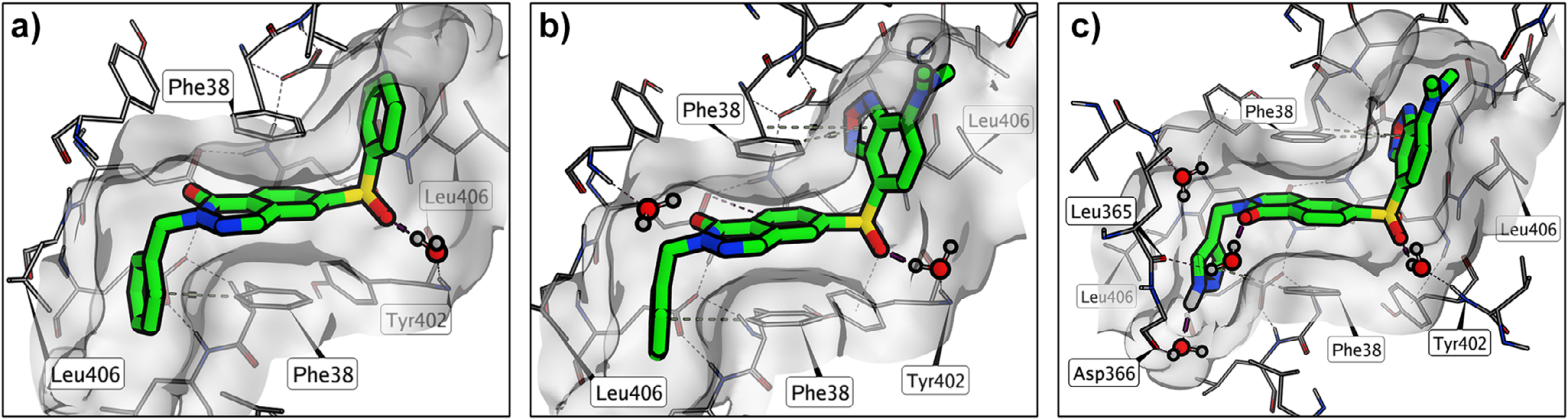
Crystal structures of compounds **I** a) (PDB ID: 9RFQ), **II** b) (PDB ID: 9RFT), and **IV** c) (PDB ID: 9RDF) in complex with PKL. The displayed ligand–protein complexes were processed through protonation of all residues (no relaxation), and energy minimization of water molecules through fixed‐position rotational sampling to find bridged interactions.

Binding of compounds **I**, **II**, and **IV** to the allosteric pocket of PKL was confirmed by X‐ray crystallography (Figure 2). Ligand‐bound crystal structures were obtained by soaking PKL crystals, and all ligands adopted Z‐shaped conformations consistent with previous PKL modulators.^[^ ^21^, ^43^ ^]^ A sulfone oxygen in each ligand formed a water‐mediated interaction with the main‐chain nitrogen of Tyr402. The phenyl groups of **I** engaged in T‐shaped π–π interactions with the phenyl rings of the two Phe38 residues and hydrophobic contacts with Leu406 (Figure 2a). Compound **II** shared these contacts and had extended interactions between the benzoxadiazole ring and the hydrophobic S1 pocket. The dimethylamine group of **II** appeared to cause a minor shift in the phthalazine core, likely due to steric clashes in the S1 subpocket. A high‐resolution structure of **IV** bound to PKL revealed a similar binding mode, with a 180° rotated phthalazine core. This orientation allowed its carboxyl oxygen to form a water‐mediated interaction with Leu365. The pyrazole moiety engaged Phe38 in π–π interactions but was more spatially compact than **II**’s phenyl group. A second water‐mediated interaction was observed between the pyrazole nitrogen and Asp366.

### Buffer Stability Testing

The chemical stability of compounds **I**–**IV** in buffered aqueous solutions was examined at physiological temperature and pH to minimize the risk of degradation‐induced artifacts in the assays. Stability was assessed in both tris‐HCl buffer and serum‐supplemented Opti‐MEM, representing conditions used in recombinant and cellular studies, respectively.

The ligands (10 µM) were incubated in either tris‐HCl buffer or Opti‐MEM buffer (1% FBS) at 37 °C, and the purities of the samples were monitored continuously for 72 h by HPLC. No signs of degradation were observed for any of the compounds in either buffer throughout the experiment (Figure S1).

### The Effect of Compounds **I**–**IV** on PK Activity

As these compounds were binding to a known allosteric site on PK, the effects of ligands **I**–**IV** on the enzymatic activities of PKL and PKM2 were determined (Figure S2). The two isozymes were incubated with increasing concentrations of compounds before the addition of substrates PEP and adenosine diphosphate (ADP), and the reactions were monitored using a Kinase‐Glo MAX assay. The activities of the samples were normalized to 100% against a DMSO control as presented in Table 1.

**Table 1:** Effects of ligands **I**–**IV** on PK activity in recombinant‐ and cell lysate‐assays. For recombinant enzyme, PKL or PKM2 (10 nM) were pre‐incubated with the compounds (0–100 µM) for 15 min, following the addition of PEP (0.1 mM) and ADP (0.2 mM), after which the activities were determined. The activity values are expressed as a percentage of the PK activity of the DMSO control. The potencies were calculated by the best fit of the Hill equation to the luminescence data.^[^ ^47^ ^]^ For HepG2 PKM2 KO cell lysates, the HepG2 PKM2 KO cells were incubated for 48 h with the compounds **I**–**IV**, mitapivat, or vehicle control (DMSO), and PKL activity was determined in cell lysates. Data are expressed as percentage of PKL activity in vehicle‐treated cells and presented as mean ± SE (*n = 3*), ^*^p < 0.05, ^**^p < 0.01, ^***^p < 0.001, ^****^p < 0.0001; One‐way ANOVA followed by Dunnett´s multiple comparison test.

	Recombinant enzyme assay				HepG2 PKM2 KO cell lysate assay		
	PKL		PKM2		PKL activities (% of vehicle)		
Compound	EC_50_ (µM)	*E*_max_ (% vehicle)	EC_50_ (µM)	*E*_max_ (% of vehicle)	0.1 µM	1 µM	10 µM
**I**	0.015	162	0.025	596	115 ± 2^***^	189 ± 3^****^	330 ± 3^****^
**II**	0.7	*120*	187	572	112 ± 1^*^	126 ± 1^****^	133 ± 6^****^
**III**	86	50^a)^	>10	323	N/D^c)^	N/D^c)^	N/D^c)^
**IV**	0.013	169	0.017	449	108 ± 3	146 ± 4^**^	242 ± 7^****^
**Mitapivat**	0.05	170	0.033^b)^	310^b)^	N/D	N/D	622 ± 12^****^

^a)^ *E*_max_ above 100% indicates activation, and *E*_max_ below 100% indicates inhibition.

^b)^ Activities as reported by Kung et al.^[^ ^46^ ^]^

^c)^ Not determined.

Reference compound **I** activated both PKL and PKM2, with similar potencies but with higher efficacy towards PKM2. In contrast, tracer **II** showed significantly reduced potency against both isoforms. Structural analysis suggests that this reduction may be due to conformational strain introduced by the dimethylamine group of **II**, which interacts unfavorably with the side chains of Leu39, Leu42, and Leu406 (Figure 2b). Concordantly, ligand **III** was even less efficient than both **I** and **II** but displayed weak inhibition against PKL at higher concentrations (>50 µM). This diminished activity is likely due to the loss of π–π interactions with Phe38, resulting from the substitution of the phenyl ring in **II** with a *tert*‐butyl group in **III**. Ligand **IV** was much more active against PKL and PKM2 compared with **II**, having a similar potency to that of **I**. Analysis of the crystal structure of **IV** suggests that this potency gain is mainly attributed to the water‐mediated interactions with Leu365 and Asp366 (Figure 2c).

### PKL Activity in Cell Extracts Derived from Compound‐Treated HepG2 PKM2 KO Cells

To test the compound on endogenous PKL, we measured the effects of **I**, **II**, and **IV** on the PKL activity of lysates from HepG2 PKM2 knock‐out (KO) cells. These predominantly express PKL. The cells were incubated with the compounds for 48 h, followed by washings, lysis, and measurement of PK activity of the cell lysates (Table 1). The reported PK activator mitapivat was included as a cell‐permeable control.^[^ ^23^, ^46^ ^]^ PKL activity was expressed as a percentage of the vehicle (DMSO)‐treated cell lysates.

Compounds **I**, **II,** and **IV** activated PKL in a dose‐dependent manner in concordance with the biochemical experiments. Ligand **II** was less effective compared with both **I** and **IV**, presumably because of its lower potency. These results also indicate that all compounds are cell‐permeable, which is a fundamental requirement for NanoBRET tracers.

### Spectroscopic Properties of Tracers **II**–**IV**

The spectroscopic properties of tracers **II–IV** were characterized in tris‐HCl buffer and MeCN using UV–vis fluorescence spectroscopy. The fluorescence spectra are shown in Figure S3, and the spectroscopic properties derived from these are summarized in Table S1. No significant differences between the absorption maxima of tracers **II**–**IV** were observed in either Tris or MeCN, indicating that the alkyl substitution pattern on the phthalazine core has little influence on these properties.

However, the tracers display a bathochromic shift in the emission bands in buffer relative to MeCN, further demonstrating moderate fluorosolvatochromism. The fluorescence of **II**–**IV** is significantly quenched in buffer, as indicated by a reduction in quantum yield (*Φ*_f_) 5.3% in buffer to 1.5% in MeCN for **IV**. This quenching effect is consistent with previous observations for the SBD class of fluorophores, which are known to undergo solvent‐induced fluorescence suppression in polar environments.^[^ ^45^, ^48^ ^]^

### Binding Affinity Measurements

Surface plasmon resonance (SPR) was used to assess binding affinities and kinetics of compounds **I**–**IV** with recombinant PKL and PKM2 (Table 2). Compound **I** displayed a *K*_D_ of 0.133 µM for PKL, while tracer **II** had 250‐fold weaker affinity (*K*_D_  =  36.2 µM), and tracer **III** showed no binding. Tracer **IV** displayed stronger PKL binding (*K*_D_  =  37 nM). For reference, the allosteric PK activator mitapivat had a *K*_D_ of 11 nM, consistent with its measured and reported AC_50_ values (50 and 37 nM, respectively).^[^ ^46^ ^]^ Despite having similar binding affinities, mitapivat exhibited a significantly lower off‐rate, approximately nine‐fold slower than tracer **IV**, indicating a more stable interaction with PKL.

**Table 2:** Kinetic binding parameters of compounds **I**–**IV** were measured towards PKL and PKM2, and binding parameters were assessed by SPR at 20 °C. The kinetic binding parameters of **IV** towards PKL were also measured using fluorescence indication titration. Data are presented as mean ± SE (*n = 3*).

SPR (PKL)			
Compound	*K*_D_ (µM)	*k*_on_ (µM^−1^min^−1^)	*k*_off_ (min^−1^)
**I**	0.133 ± 0.008	2.47 ± 0.53	0.32 ± 0.052
**II**	36.2 ± 0.289	0.01 ± 0.001	0.50 ± 0.001
**III**	>100^a)^	–^b)^	–^b)^
**IV**	0.037 ± 0.001	10.08 ± 0.173	0.37 ± 0.01
**Mitapivat**	0.011 ± 0.001	3.68 ± 0.22	0.04 ± 0.006
SPR (PKM2)			
Compound	*K*_D_ (µM)	*k*_on_ (µM^−1^min^−1^)	*k*_off_ (min^−1^)
**I**	0.033 ± 0.001	0.27 ± 0.01	<0.033^c)^
**II**	>100^a)^	–^b)^	–^b)^
**III**	>100^a)^	–^b)^	–^b)^
**IV**	1.37 ± 0.218	0.59 ± 0.015	0.8 ± 0.109
**mitapivat**	0.032 ± 0.002	0.29 ± 0.022	<0.033 ± 0^c)^
Fluorescence titration (PKL)			
Compound	*K*_D_ (µM)	*k*_on_ (µM^−1^min^−1^)	*k*_off_ (min^−1^)
**IV**	0.042 ± 0.007	12.376 ± 0.74	0.519 ± 074^d)^

^a)^ No binding observed.

^b)^ Not applicable.

^c)^ Undetermined due to very slow dissociation.

^d)^ Indicated errors were calculated according to the Gaussian law of error propagation.

Against PKM2, *K*_D_ values were 33 nM for **I**, 1370 nM for **IV**, and 32 nM for mitapivat, with no detectable binding observed for **II** or **III**. These findings align with the activity measurements, indicating that **II** selectively engages PKL, while **IV** has moderate PKL selectivity (37‐fold). Due to their weak affinities and poor solubilities, **II** and **III** were not pursued further.

The binding kinetics of **IV** were further validated with fluorescence titration experiments conducted with recombinant PKL. As previously reported, SBD‐tagged PKL ligands fluorescence upon binding to the allosteric pocket,^[^ ^43^ ^]^ enabling *K*_D_ determination by saturation binding. In microplate assays, **IV** produced a dose‐responsive increase in fluorescence upon PKL binding (Figure S4), yielding a *K*_D_ of 42 nM after Hill equation fitting (Equation S1).^[^ ^47^ ^]^ Association kinetics were then measured by adding tracer **IV** to PKL and monitoring fluorescence over time. Data were fit using an exponential association model (Equation S2),^[^ ^49^ ^]^ and *k*_on_ was derived from the reciprocal analysis of recorded *k*_obs_ values. The *k*_off_ was subsequently calculated (Equation S3).^[^ ^49^ ^]^ The fluorescent binding experiments were consistent with SPR results, confirming a mid‐nanomolar *K*_D_ of **IV**.

### Live‐Cell Uptake Studies of Tracer **IV**

**Figure 3:**
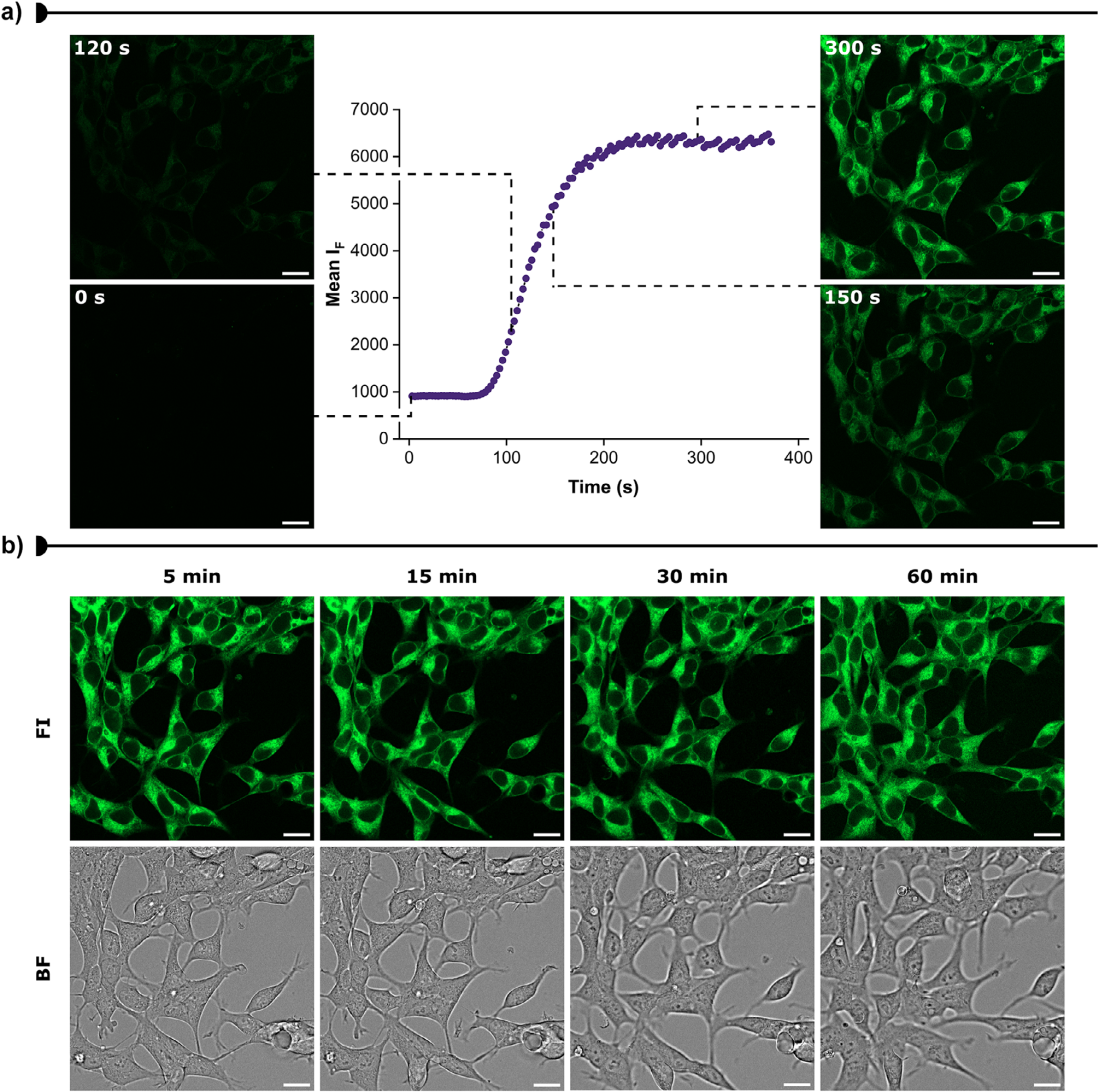
Live‐cell imaging of HEK293 cells after addition of tracer **IV** (1 µM). a) Changes in fluorescence emission upon excitation at 488 nm during the cellular uptake of **IV**. Fluorescence emission (*λ*_ex_ = 488 nm, green) was recorded immediately after the addition of **IV** to HEK293 cells. Fluorescence images after 0, 120, 150, and 300 s of incubation time are displayed next to the time curve. Dashed lines indicate which portion of the curve the images correspond to. b) Confocal microscopy images of HEK293 cells showing the intracellular fluorescence of **IV** (*λ*_ex_ = 488 nm, green, 1 µM) after 5, 15, 30, and 60 min of incubation at 37 °C, 5% CO_2_. The fluorescent images correspond to the middle slice taken from the respective Z‐stacks. The corresponding bright‐field images are also shown. Scale bars: 20 µm. FI = Fluorescence intensity, BF = Brightfield.

To investigate the cellular permeability of **IV**, we employed confocal microscopy to monitor the uptake of the tracer in live HEK293 cells over time. The tracer was added to live cells at a final concentration of 1 µM, and real‐time fluorescence intensity was recorded immediately after addition. A rapid increase in fluorescence was observed, reaching a stable plateau approximately 4 min after addition, indicating fast cellular uptake and membrane permeabilization (Figure 3a and Video S1). In contrast, control cells treated with DMSO exhibited negligible background fluorescence (Figure S5), confirming that the observed signal originated predominantly from tracer **IV**.

Fluorescence intensity was then continuously monitored for 60 min. To assess potential fluorophore degradation, corrected total cell fluorescence (CTCF) was calculated at each time point (Figure S6). Fluorescence values from regions of interest (ROIs) were integrated and normalized cell area and background signals in the absence of tracer. The fluorescence signal from tracer **IV** remained stable throughout the 60‐min incubation, and bright‐field imaging showed no significant changes in cell morphology, although some lateral displacement of the semi‐adherent cells was noted (Figure 3b). While these results do not confirm long‐term tracer stability, they suggest that tracer **IV** remains intact within cells over the observed timeframe.

Photostability of tracer **IV** was subsequently evaluated by treating HEK293 cells with the tracer (1 µM) for 2 h, followed by continuous scanning using 488 nm laser excitation for 10 min. Fluorescence intensity exhibited only a minor, time‐dependent decrease of approximately 6%, indicating good photostability under these conditions (Figure S7).

Finally, the effects of tracer **IV** on cell viability were assessed. HEK293 cells were treated with **IV** at concentrations of 0, 0.01, 0.1, 1, and 10 µM for 2 h, after which cells were lysed, and intracellular ATP levels were quantified using the CellTiter‐Glo 2.0 assay (Figure S8a). Similar ATP levels were observed in both compound‐ and vehicle‐treated cells, demonstrating that compound **IV** does not affect cell viability.

### Expression and Biochemical Activity of Recombinant PKL_Nluc_

NanoBRET assays operate on the principle of BRET, which enables detection of ligand‐target interactions through energy transfer between donor and acceptor molecules.^[^ ^33^ ^]^ Analogous to Förster resonance energy transfer (FRET),^[^ ^50^ ^]^ BRET efficiency depends on factors such as spectral overlap and the distance between donor and acceptor moieties.^[^ ^51^ ^]^ A strong spectral overlap between the emission spectra of the donor and the acceptor excitation spectrum enhances energy transfer, while efficiency sharply declines with increasing donor–acceptor distance.^[^ ^51^ ^]^

In NanoBRET, the donor is Nluc, a small and bright luciferase enzyme.^[^ ^52^ ^]^ When fused to a target protein, the spatial positioning of Nluc relative to the ligand‐binding site becomes critical. To assess this in our system, we modeled Nluc fused to the *N*‐terminus of PKL (PKL_Nluc_) using AlphaFold 3.^[^ ^53^ ^]^ The model predicted an average distance of approximately 25 Å between the catalytic site of Nluc and the allosteric pocket in PKL (Figure S9), within a range conducive to efficient resonance energy transfer.^[^ ^50^ ^]^ To test the functionality of PKL_Nluc_, the protein was expressed and purified, and Kinase‐Glo MAX assays were performed. The biochemical assay showed that PKL_Nluc_ is catalytically active, exhibiting slightly higher activity levels than wild‐type PKL (PKL_wt_) (Figure S10). To probe the allosteric regulation of PKL_Nluc_, we tested both PKL_wt_ and PKL_Nluc_ with the known allosteric modulator mitapivat^[^ ^46^ ^]^ (activator) and urolithin D^[^ ^22^ ^]^ (inhibitor) at 10 µM. Both enzymes responded as expected to these ligands, with PKL_Nluc_ displaying modulation patterns like PKL_wt_, although mitapivat had a marginally stronger activating effect on PKL_wt_. These findings confirm that *N*‐terminal fusion of Nluc to PKL_wt_ does not compromise the catalytic function of PKL or disrupt allosteric communication between the allosteric and active sites.

### Tracer **IV** Selectively Binds to the Tetramers of Recombinant PKL_Nluc_ and PKL_wt_

**Figure 4:**
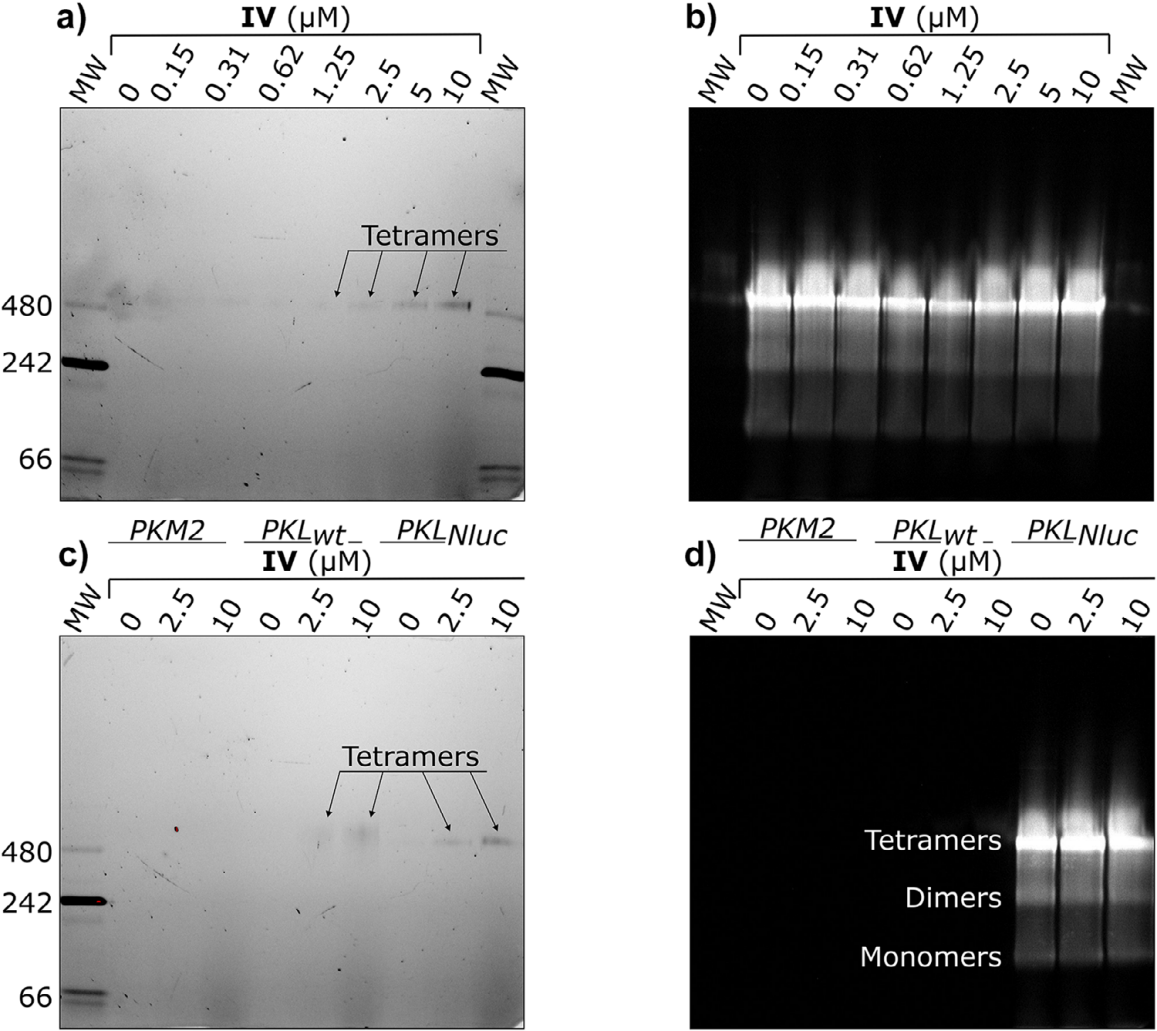
Native PAGE experiments demonstrate engagement of tracer **IV** with the tetramers of PKL_Nluc_ and PKL_wt_. a) Binding of tracer **IV** to PKL_Nluc_ recombinant protein as assessed by in‐gel fluorescent detection of the protein after incubation for 1 h at room temperature with different concentrations of **IV** and separation by native PAGE using 3%–12% bis‐tris gel. MW markers are labeled on the left side of the gel. b) In‐gel luminescence detection of PKL_Nluc_ after addition of Nluc substrate and incubation at room temperature for 5 min. Panels A and B were captured from the same gel. c) Binding of tracer **IV** to PKM2, PKL, and PKL_Nluc_ recombinant proteins as assessed by in‐gel fluorescent detection of the labeled proteins after incubation for 1 h at room temperature with different concentrations of **IV** and separation by native PAGE using 3%–12% bis‐tris gel. MW markers are labeled on the left side of the gel. d) In‐gel luminescence detection of PKL_Nluc_ after addition of Nluc substrate as described in panel B. Arrows indicate the tetramer in the respective lane.

To study the interaction of **IV** with PKL_Nluc_ and its oligomers, native PAGE was performed (Figure 4). Recombinant PKL_Nluc_ was incubated for 2 h with **IV** (0.15–10 µM) or DMSO and loaded onto gels. In‐gel fluorescence showed a single band at 1.25–10 µM **IV** that increased with concentration, indicating dose‐dependent PKL_Nluc_ labeling (Figure 4a). Luminescence imaging post‐substrate addition revealed three bands representing tetramers, dimers, and monomers (Figure 4b and Video S2), with most luminescence coming from the tetramers. Colloidal blue staining and western blotting with anti‐PKL antibodies confirmed the predominance and identity of the bands (Figure S11a,d). Overlaying fluorescence and luminescence images showed **IV** bound only the tetramer (Figure S11c). PKM2 and PKL exist in equilibrium between oligomeric states, with PKM2 mainly dimeric when unbound.^[^ ^18^ ^]^ As oligomerization regulates PK function,^[^ ^11^, ^54^ ^]^ PKL_Nluc_ oligomerization was compared to PKL_wt_ and PKM2 (Figure 4b,c). Native PAGE repeated with all three proteins incubated with **IV** (2.5 or 10 µM) or DMSO showed **IV** labeling only of the tetramers of PKL_Nluc_ and PKL_wt_, while no fluorescence was detected in PKM2 lanes (Figures 4c,d and S11). Colloidal blue and anti‐PKM2 blots confirmed PKM2 was mostly monomeric, explaining the lack of binding (Figure S11). Since SPR showed micromolar affinity of **IV** for PKM2 (Table 2), the assay was repeated with FBP to induce tetramerization.^[^ ^6^ ^]^

With **IV** (2.5 or 10 µM) and FBP (10 µM), the gels showed enhanced **IV** fluorescence for PKL_wt_ but not for PKL_Nluc,_ while PKM2 showed no fluorescence despite being tetrameric as shown by staining and western blot (Figures 4c and S12). Given that FBP levels vary widely in cells,^[^ ^55^, ^56^, ^57^ ^]^ and that it binds PKL with nanomolar affinity, ^[^ ^58^ ^]^ **IV** binding to PKL_wt_ was tested with and without FBP. Fluorescence titrations showed no significant change in binding affinity (Figure S4), though FBP‐treated samples had higher fluorescence, aligning with gel data (Figures S11 and S12).

These results demonstrate that PKL maintains its structure and function when fused with Nluc, and **IV** binds selectively to the tetramer. The FBP‐enhanced fluorescence may benefit NanoBRET assays, especially in FBP‐rich cell lines like HEK293.^[^ ^55^ ^]^

### NanoBRET Assay Validation

To investigate the intracellular interaction of tracer **IV** with PKL_Nluc_, we prepared a mammalian expression construct, fusing Nluc to the *N*‐terminus of PKL. Following transient transfection in HEK293 cells, expression of PKL_Nluc_ was confirmed by SDS‐PAGE analysis followed by immunoblotting with antibodies against PKL (Figure S13). As a negative control for NanoBRET experiments, a construct expressing Nluc alone (without PKL) was also prepared.

**Figure 5:**
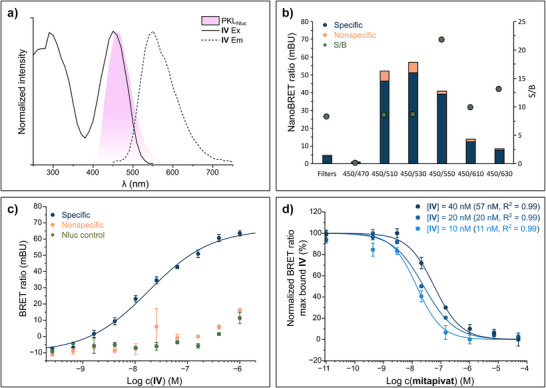
Optimization of plate reader settings and conditions for the NanoBRET assay. a) Overlaid excitation/emission (Ex/Em) spectra of tracer IV (solid and dashed lines, respectively) and luminescence spectra (blue area) of recombinant PKLNluc show that there is a spectral overlap between 400–500 nm. b) Optimization of luminescence wavelength pairs in the NanoBRET system with tracer IV (0.1 µM) and recombinant PKLNluc, relative intensities of specific (specific, dark blue) and nonspecific (10 µM mitapivat, orange) signals are shown. The signal‐to‐background (S/B) ratio (green circles) is expressed as the ratio between the specific and nonspecific samples and is displayed on a secondary axis (right). c) HEK293 cells were transiently transfected with PKLNluc or Nluc plasmids for 24 h and treated thereafter for 2 h at 37 °C with different concentrations of IV. The cells transfected with PKLNluc plasmid were treated both in the absence (specific, dark blue) and presence of 20 µM mitapivat (nonspecific control, orange). BRET ratios were calculated based on the luminescence at 450 nm (donor) and 550 nm (acceptor). Data is presented as mean ± SE (*n* = 3), error bars represent the SE. EC50 values were determined by a four‐parameter fit to the Hill equation (Equation S1).^[^ ^47^ ^]^ d) Competition experiments with mitapivat at different concentrations of tracer **IV** in live HEK293 cells. HEK293 cells were transiently transfected with PKL_Nluc_ plasmid for 24 h and treated thereafter for 2 h at 37 °C with tracer **IV** and mitapivat. BRET ratios were calculated based on the luminescence at 450 nm (donor) and 550 nm (acceptor). Data are presented as mean ± SE (*n = *3), error bars represent the SE. EC_50_ values were determined by a four‐parameter fit to the Hill equation (Equation S1).^[^ ^47^ ^]^ Figure legend: concentrations of tracer **IV** per titration, parentheses show the calculated EC_50_ from Hill fit and the coefficient of determination (R^2^).

To our knowledge, SBD has not been used in previous BRET applications, though structurally related 4‐amino‐7‐nitrobenzoxadiazole dyes have been applied in such systems.^[^ ^59^ ^]^ Due to the low quantum yield of SBD‐containing **IV** (Table S1), we optimized the plate reader settings for the NanoBRET assay to maximize the signal‐to‐background (S/B) ratio. The fluorescence excitation spectrum of tracer **IV** overlapped with the emission band of PKL_Nluc_ (Figure 5a), prompting the screening of wavelength pairs within 400–600 nm. Tracer **IV** (100 nM) was incubated with recombinant PKL_Nluc_ and Furimazine, and BRET ratios were measured with or without saturating concentrations of the commercial competitor mitapivat (20 µM, *K*_D_ = 11 nM) (Figure 5b). The screening revealed that 450/550 nm yielded the optimal S/B ratio while preserving a relatively large assay window (39 mBU).

Incubation of HEK293 cells transiently transfected with the PKL_Nluc_ plasmid and treated with tracer **IV** resulted in a dose‐dependent BRET signal (Figure 5c).

Additional titrations with saturating concentration of mitapivat resulted in a minimal response, comparable to the negative control, in which only Nluc was expressed.

Nonspecific binding signals emerged at tracer concentrations exceeding 400 nM, and subtracting the displacement control from the tracer titration yielded the specific signal, which, when fitted to the Hill equation, gave an EC_50_ of 18.4 nM. Additionally, cell viability assays were conducted on cells transfected with either PKL_Nluc_ or Nluc plasmids and incubated with tracer **IV** (Figure S4b,c). The tracer did not affect the viability of the two cell types up to the highest tested concentration.

To determine the optimal tracer concentration, competition experiments with mitapivat were performed at 10, 20, and 40 nM of tracer **IV** (Figure 5d). All samples exhibited a dose‐dependent decrease in BRET ratio, with progressively increasing EC_50_ values of 11, 20, and 57 nM for the 10, 20, and 40 nM tracer concentrations, respectively. Correcting for displacement effects using Cheng–Prusoff analysis (Equation S4)^[^ ^60^ ^]^ yielded estimated affinities of 7.2, 9.6, and 18 nM, respectively. Since the 20 nM tracer concentration was closest to the *K*_D_ of mitapivat determined by SPR, this concentration was selected for further experiments.

### Competition Experiments with Reported Allosteric PK Modulators

**Figure 6:**
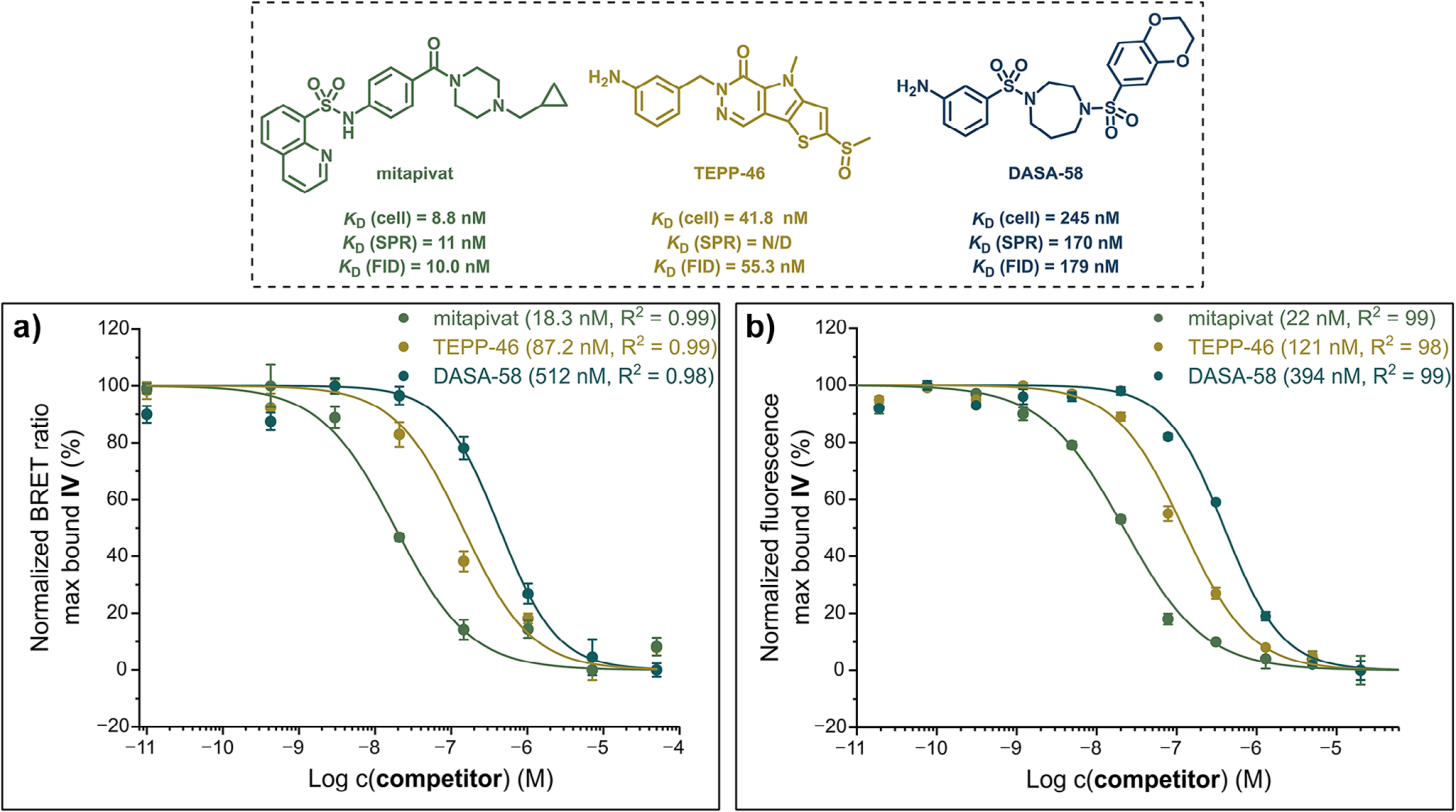
Competitive titrations with known allosteric modulators mitapivat, DASA‐58, and TEPP‐46. (Dashed panel) comparison of *K*_D_ values measured through cellular NanoBRET assay, SPR (20 °C), and FID for mitapivat, TEPP‐46, and DASA‐58. a) HEK293 cells transiently transfected with PKL_Nluc_ plasmid for 24 h and then incubated for 2 h at 37 °C with serially diluted concentrations of competitors in the presence of 20 nM of tracer **IV**. BRET ratios were calculated based on the luminescence at 450 nm (donor) and 550 nm (acceptor). Data is presented as mean ± SE (*n = *3), error bars represent the SE. EC_50_ values were determined by a four‐parameter fit to the Hill equation (Equation S1).^[^ ^47^ ^]^ b) FID experiments with mitapivat, TEPP‐46, and DASA‐58. Recombinant PKLwt was incubated with serially diluted concentrations of competitors in the presence of 50 nM of tracer IV for 30 min at ambient temperature. Data are presented as mean ± SE (n = 3), error bars represent the SE. EC_50_ values were determined by a four‐parameter fit to the Hill equation (Equation S1).^[^ ^47^ ^]^

With a functional NanoBRET assay for PKL in place, we aimed to assess the intracellular interactions of representative allosteric ligands with PKL_Nluc_. Titrations with mitapivat, one of the most potent known PK ligands,^[^ ^24^ ^]^ have already shown that this compound binds competitively to PKL_Nluc_ within cells (Figure 5d). To compare mitapivat with ligands of varying potency, mitapivat, TEPP‐46, and DASA‐58 were titrated against tracer **IV**, yielding displacement curves with EC_50_ values of 18.3, 87.2, and 512 nM, respectively (Figure 6a). Applying Cheng–Prusoff corrections (Equation S4),^[^ ^60^ ^]^ the affinities were estimated to be 8.8, 41.8, and 245 nM for mitapivat, TEPP‐46, and DASA‐58, respectively.

Additionally, we performed fluorescence competition assays with these competitors against **IV**, using recombinant PKL_wt_ (Figure 6b). PKL_wt_ and **IV** were incubated with increasing concentrations of mitapivat, TEPP‐46, and DASA‐58, and the fluorescence of **IV** (*λ*_ex_ = 450 nm, *λ*_em_ = 550 nm) was quantified. Fitting the titration data to the Hill equation and applying Cheng–Prusoff corrections gave estimated *K*_D_ values of 10, 55, and 179 nM for mitapivat, TEPP‐46, and DASA‐58, respectively. The binding affinities measured in the cellular experiments align with SPR and fluorescence indicator displacement (FID) measurements. Additionally, mitapivat, TEPP‐46, and DASA‐58 exhibit progressively weaker binding, consistent with previously reported findings.^[^ ^43^ ^]^ Earlier generations of DASA‐derived fluorescent allosteric PKL ligands were not as potent (EC_50_ > 10 µM), which imposed a detection limit on potent binders in recombinant binding assays. These NanoBRET results support our hypothesis that the prior setup was affected by depletion effects due to the high concentrations of PKL used, which led to similar observed affinities for mitapivat and TEPP‐46.

Overall, these findings establish **IV** as a useful tool for tracking allosteric PKL ligand engagement in living cells. Furthermore, the environment‐sensitive properties of **IV** enable its application in both recombinant and cellular systems.

## Conclusion

A versatile tracer for monitoring ligand engagement to the allosteric pocket of PKL has been developed. This tracer allows for binding affinity determination of unlabeled compounds in both a cellular and a recombinant context.

By building upon prior limitations of low‐affinity DASA‐derived compounds, the incorporation of an environment‐sensitive SBD fluorophore into a phthalazine scaffold yielded **LumiPK** (**IV**). The tracer demonstrated nanomolar binding affinity (*K*_D_ = 37 ± 5 nM) for PKL, and modest selectivity (37‐fold) for PKL over PKM2, as indicated by SPR, fluorescence titrations, and functional kinase assays. Importantly, native PAGE experiments demonstrated **LumiPK** to bind exclusively to the tetrameric form of PKL, and not to monomeric or dimeric oligomers, thus offering a structurally and functionally relevant readout. Crystallographic studies confirmed that **LumiPK** engages the allosteric site in the tetrameric form of PKL, with key water‐mediated interactions contributing to its enhanced potency.

The tracer exhibited buffer stability, rapid membrane permeability, and did not affect the cell viability of HEK293 cells, making it suitable for use in live‐cell assays.

A NanoBRET assay using a PKL‐Nluc fusion protein was established, with **LumiPK** serving as tracer. Competitive displacement experiments with known PKL activators (mitapivat, TEPP‐46, DASA‐58) confirmed intracellular ligand engagement and aligned well with recombinant protein assays. These findings validate **LumiPK** as a robust tool for studying allosteric PKL modulation in live cells.

Additionally, **LumiPK** now enables the development of high‐throughput screening systems for novel PKL‐targeted therapeutics. Importantly, the allosteric site is conserved among PK isoforms, prompting investigation into the broader applicability of this tracer beyond PKL. Overall, this work addresses a critical gap in target engagement technologies for PKL and enables future exploration of allosteric modulation in metabolic diseases.

## Author Contributions

**O.N., M.G**., and **M.H**.: Conceptualization. **O.N**., **L.H**., **A.P.V**., **A.B**., **I.K**., **A.G**., and **M.R**.: Methodology. **O.N**., **A.P.V**., **L.H**., **A.B**., **I.K**., **P.B**., **M.R**., **A.G**., **M.H**., and **M.G**.: Validation; data interpretation; review and editing. **O.N**.: Writing. All authors have read and agreed to the published version of the manuscript.

## Conflict of Interests

The authors declare no conflict of interest.

## Color Palettes and Illustrations

The scientific color map *Batlow* is used in this study to minimize visual distortion of data and illustrations for the convenience of readers with color‐vision deficiencies.^[^ ^65^ ^]^ The Protein Imager was used to create molecular representations of proteins in this study.^[^ ^66^ ^]^

## Supporting Information

The data that support the findings of this study are available in the supplementary material of this article. The authors have cited additional references within the Supporting Information.^[^ ^21^, ^22^, ^46^, ^47^, ^49^, ^53^, ^60^, ^61^, ^62^, ^63^, ^64^ ^]^

## Supporting information



Supporting Information

Supporting Information

Supporting Information

## Data Availability

The data that support the findings of this study are available from the corresponding author upon reasonable request.
