# Supplementary material for: Potent Fluorescent Probe for Target‐Engagement Studies of Allosteric Pyruvate Kinase Modulators
Source: Angew Chem Int Ed Engl. 2025 Aug 29;64(42):e202513969. doi: 10.1002/anie.202513969 (PMC12518693; doi:10.1002/anie.202513969)
Supplement: Supplementary file 1 — Supporting Information [file ANIE-64-e202513969-s002.docx]

**SUPPORTING INFORMATION**

Potent Fluorescent Probe for Target-Engagement Studies of Allosteric Pyruvate Kinase Modulators

Oscar Nilsson,^[a]^ Anna P. Valaka,^[a]^ Liliana Håversen,^[b]^ Agnieszka Bogucka,^[c]^ István Köteles,^[a]^ Paul Brear,^[c]^ Mikael Rutberg,^[b]^ Anders Gunnarsson,^[d]^ Marko Hyvönen,^[c]^ Morten Grøtli*^[a]^

[a] MSc. O. Nilsson, MSc. A. P. Valaka, Dr. I. Köteles, Prof. M. Grøtli
Department of Chemistry and Molecular Biology
University of Gothenburg
Medicinaregatan 7B, SE-413 90, Gothenburg, Sweden
E-mail: grotli@chem.gu.se

[b] Dr. L. Håversen, Dr. M. Rutberg
Department of Molecular and Clinical Medicine
University of Gothenburg and Sahlgrenska University Hospital
SE-413 45, Gothenburg, Sweden

[c] Dr. A. Bogucka, Dr P. Brear, Prof. M. Hyvönen
Department of Biochemistry
University of Cambridge
80 Tennis Court Road, Cambridge CB2 1GA, UK

[d] Dr. A. Gunnarsson
Discovery Sciences, R&D Gothenburg, AstraZeneca
SE-431 83 Mölndal, Sweden

* Corresponding author

Supporting information for this article is given via a link at the end of the document.

**Table of contents**

[Synthetic chemistry 3](#_Toc205814279)

[Supplementary figures and tables 9](#_Toc205814280)

[Equations 24](#_Toc205814281)

[Experimental procedures 24](#_Toc205814282)

[*X-ray crystallography* 24](#_Toc205814283)

[*Buffer stability measurement of compounds* 24](#_Toc205814284)

[*PKL activity assays* 25](#_Toc205814285)

[*HepG2 cellular PKL activity assay* 25](#_Toc205814286)

[*Characterization of fluorescent properties* 25](#_Toc205814287)

[*Surface plasmon resonance* 26](#_Toc205814288)

[*Fluorescence indication titrations of tracer IV* 26](#_Toc205814289)

[Confocal microscopy 26](#_Toc205814290)

[*HEK293 cell viability assay (CellTiter-Glo 2.0)* 27](#_Toc205814291)

[*AlphaFold structure model of PKL_Nluc_ fusion protein* 27](#_Toc205814292)

[*Molecular cloning of expression constructs* 28](#_Toc205814293)

[*Native PAGE electrophoresis* 28](#_Toc205814294)

[*NanoBRET Experiments with recombinant PKL_Nluc_* 28](#_Toc205814295)

[*Protein production* 29](#_Toc205814296)

[*Overexpression of PKL_Nluc_ in HEK293 cells* 29](#_Toc205814297)

[*Live-cell NanoBRET experiments with HEK293 cells* 30](#_Toc205814298)

[*Fluorescence indication competition assays* 30](#_Toc205814299)

[LC-MS Chromatograms 31](#_Toc205814300)

[NMR Spectra 33](#_Toc205814301)

[References 66](#_Toc205814302)

# **Synthetic chemistry**

Chemical synthesis and characterization

*General information*

Unless otherwise specified, all reagents were obtained from commercial supplier Sigma-Aldrich and used without further purification. Solvents were dried using a solvent purification system (PS-MD-5/7 Inert technology). Microwave reactions were performed in a Biotage Initiator+ system with fixed hold time. Reactions were monitored by LC-MS (Waters™ Acuity Arc/QDa LC-MS system; XBridge® C18 3.5µM column, 2.1 × 50 mm, Part. No. 186003021; H_2_O:CH_3_CN (0.1% formic acid)) or by TLC (silica gel 60 F254, Merck). The TLC plates were visualized by UV light (λ = 254 nm). Flash-column chromatography was performed using Biotage Isolera One or Selekt flash chromatography systems with silica gel SNAP KP-Sil, Sfär cartridges or reversed phase Sfär C18 columns. Melting points were determined with a Büchi B-545 apparatus. NMR spectra were recorded on a Bruker 400 MHz and a Bruker Avance NEO 600 MHz spectrometer at 25 °C. All chemical shifts are reported in parts per million (δ) relative to the residual solvent peak. The following abbreviations are used to denote signal patterns: (s) singlet, (d) doublet, (t) triplet, (q) quartet, (m) multiplet, and (br) broad, unless otherwise noted. Coupling constants (J) are reported in Hertz (Hz). High-resolution mass spectrometry (HRMS) data were acquired using a QExactive HF Orbitrap mass spectrometer coupled to a Dionex Ultimate 3000 liquid chromatography system (Thermo Fisher Scientific). The instrument operated exclusively in full MS mode, acquiring ion mass spectra at a resolution of 120,000 with a maximum injection time of 200 ms for up to 3×10⁶ ions. Prior to analysis, the Orbitrap was calibrated using the Pierce LTQ ESI Positive Ion Calibration Solution, achieving a mass accuracy better than 5 ppm. Electrospray ionization was carried out using a metal emitter at 4 kV and 320 °C. Sample volumes of either 1 or 10 μL were injected onto a reversed-phase XBridge BEH C18 column (3.5 μm, 2.1 × 50 mm, Waters). Chromatographic separation was achieved using a linear gradient from 10% to 100% solvent B over 3 minutes, followed by an isocratic elution with 100% solvent B for 7 minutes, at a flow rate of 0.300 mL/min. Solvent A consisted of water with 0.1% formic acid, while solvent B was composed of 80% acetonitrile in water with 0.1% formic acid. Data were processed using Xcalibur software (Thermo Fisher Scientific).

*Chemical Synthesis*

*3-(Chloromethyl)-1H-pyrazol-1-ium hydrochloride (****1****)*

A round-bottom flask (25 mL) equipped with a stir bar was charged with thionyl chloride (7.25 mL, 100 mmol, 10 eq). The flask was sealed and cooled in an ice bath for 15 min. 3-(Hydroxymethyl)pyrazole (981 mg, 10 mmol, 1 eq) was carefully added in portions and the reaction was stirred at ambient temperature for 2 h until completion, as indicated by TLC (KMnO_4_ stain). Volatiles were removed under reduced pressure, and the crude was recrystallized from boiling MeCN to afford the title compound as yellow crystals (1.25 g, 8.137 mmol, 81%); R_f_ = 0.34 (5% MeOH/DCM); ^1^H NMR (600 MHz, CD_3_OD) δ 8.15 (d, *J* = 2.6 Hz, 1H), 6.78 (d, *J* = 2.6 Hz, 1H), 4.84 (s, 2H); ^13^C NMR (151 MHz, CD_3_OD) δ 147.3, 135.5, 108.1, 35.3.

*3-(Chloromethyl)-1-(tetrahydro-2H-pyran-2-yl)-1H-pyrazole (****2****)*

A round-bottom flask (25 mL) equipped with a stir bar was charged with **1** (700 mg, 4.575 mmol, 1 eq), MeCN (10 mL) and 2,3-dihydropyran (1.25 mL, 13.724 mmol, 3 eq). The reaction was stirred at ambient temperature for 18 h until completion, as indicated by TLC. The mixture was concentrated under reduced pressure, and the residue was loaded onto silica and purified by flash column chromatography (SNAP 25 g, 5-20% EtOAc/n-pentane, 20 CV) to afford the title compound as a clear oil (680 mg, 3.389 mmol, 74%); R_f_ = 0.39 (5% EtOAc/n-pentane); ^1^H NMR (600 MHz, CDCl_3_) δ 7.56 (d, *J* = 2.5 Hz, 1H), 6.36 (d, *J* = 2.4 Hz, 1H), 5.33 (dd, *J* = 9.5, 2.5 Hz, 1H), 4.62 (s, 2H), 4.09 – 4.04 (m, 1H), 3.72 – 3.66 (m, 1H), 2.14 – 2.02 (m, 3H), 1.72 – 1.66 (m, 2H), 1.62 – 1.58 (m, 1H); ^13^C NMR (151 MHz, CDCl_3_) δ 149.4, 129.1, 105.9, 87.8, 68.1, 39.2, 30.6, 25.0, 22.6.

*N,N-Dimethylbenzo[c][1,2,5]oxadiazol-4-amine (****3****)*

A microwave vial (5 mL) equipped with a stir bar was charged with 4-chloro-2,1,3-benzoxadiazole (1.0 g, 6.5 mmol, 1 eq) and Me_2_NH (5.6M in EtOH, 23.2 mL, 130.0 mmol, 20 eq), and the reaction was stirred at 130 °C (10 bar) under microwave irradiation for 8 h until completion, as indicated by TLC. The mixture was concentrated under reduced pressure and partitioned between EtOAc (30 mL) and aqueous NaOH (1M, 20 mL). The organic fraction was isolated, washed with brine (2x10 mL), dried over anhydrous Na_2_SO_4_, filtered and concentrated under reduced pressure to afford the title compound as a red liquid (1025 mg, 6.282 mmol, 97%); R_f_ = 0.43 (2 % EtOAc/n-pentane); ^1^H NMR (600 MHz, CDCl_3_) δ 7.25 – 7.19 (m, 1H), 7.07 – 6.99 (m, 1H), 6.04 (dd, *J* = 7.4, 3.1 Hz, 1H), 3.30 (s, 6H). NMR spectra is in accordance with previously published data.^[1]^ The product was used in the next step without further purification.

*7-(Dimethylamino)benzo[c][1,2,5]oxadiazole-4-sulfonyl chloride (****4****)*

A round-bottom flask (25 mL) equipped with a stir bar was charged with **3** (890 mg, 5.454 mmol, 1 eq), HSO_3_Cl (1.85 mL, 27.271 mmol, 5 eq), and the reaction was stirred at 100 °C for 20 min until completion, as indicated by TLC. The mixture was cooled to ambient temperature, poured into a mixture of ice and Na_2_HPO_4_ (50% w/v). The precipitate was collected by vacuum filtration, washed with ice-cooled H_2_O (2x5 mL) and dried under reduced pressure to yield the title compound as a red solid (1102 mg, 4.211 mmol, 77%); R_f_ = N/D; ^1^H NMR (600 MHz, DMSO) δ 7.55 (d, *J* = 7.8 Hz, 1H), 6.16 (d, *J* = 7.8 Hz, 1H), 3.28 (s, 6H); ^13^C NMR (151 MHz, DMSO) δ 147.0, 145.2, 139.6, 131.7, 121.9, 103.5, 41.7.

*7-(Dimethylamino)benzo[c][1,2,5]oxadiazole-4-thiol (****5****)*

A round-bottom flask (100 mL) equipped with a stir bar was charged with **4** (860 mg, 3.286 mmol, 1 eq), SnCl_2_ (1.87 g, 9.859 mmol, 3 eq) and 1,4-dioxane (30 mL). The flask was cooled in an ice bath for 10 min, HCl (12M, 1.37 mL, 16.432 mmol, 5 eq) was added slowly and the reaction was stirred at ambient temperature for 3 h until completion, as indicated by TLC. The mixture was concentrated under reduced pressure, and the residue partitioned between EtOAc (30 mL) and H_2_O (20 mL). The aqueous fraction was isolated and extracted with EtOAc (2x10 mL). The organic fractions were combined, washed with brine (2x10 mL), dried over anhydrous Na_2_SO_4_, filtered and concentrated under reduced pressure. The residue was loaded onto silica and purified by flash column chromatography (SNAP 25g, 20% EtOAc/n-pentane, 10 CV) to afford the title compound as a red crystalline solid (256 mg, 1.311 mmol, 40%); R_f_ = 0.13 (5% EtOAc/n-pentane); ^1^H NMR (600 MHz, CDCl_3_) δ 7.18 (d, *J* = 7.8 Hz, 1H), 6.00 (d, *J* = 7.8 Hz, 1H), 3.73 (s, 1H), 3.29 (s, 6H); ^13^C NMR (151 MHz, CDCl_3_) δ 151.5, 145.8, 138.9, 134.6, 105.7, 101.9, 42.1.

*2-Benzyl-6-bromophthalazin-1(2H)-one (****6a****)*

A round-bottom flask (10 mL) equipped with a stir bar was charged with NaH (90%, 107 mg, 4.0 mmol, 3 eq) and NaI (200 mg, 1.333 mmol, 1 eq). The flask was sealed, flushed with N_2_, anhydrous DMF (4 mL) was added and the mixture was stirred in an ice bath for 5 min. 6-Bromophthalazin-1(2H)-one (300 mg, 1.333 mmol, 1 eq) was added under a stream of N_2_ in small portions, the flask was resealed and the mixture stirred in the ice bath for 5 min. Benzyl bromide (0.48 mL, 4.0 mmol, 3 eq) was added dropwise and the mixture was stirred in the ice bath for 15 min, and at ambient temperature for 45 min until complete consumption of the starting material, as indicated by TLC. The mixture was poured over ice (40 mL) and stirred for 15 min. The mixture was extracted with EtOAc (3x15 mL), the organic fractions were combined, washed with LiCl (5%, 4x5 mL), brine (10 mL), dried over anhydrous Na_2_SO_4_, filtered and concentrated under reduced pressure. The residue was loaded onto silica and purified by flash column chromatography (SNAP 25g, 5% EtOAc/n-pentane, 10 CV) to afford the title compound as a light yellow solid (334 mg, 1.060 mmol, 80%); R_f_ = 0.17 (5 % MeOH/DCM); ^1^H NMR (600 MHz, CDCl_3_) δ 8.29 (d, *J* = 8.3 Hz, 1H), 8.09 (s, 1H), 7.88 – 7.82 (m, 2H), 7.47 – 7.44 (m, 2H), 7.33 (t, *J* = 7.5 Hz, 2H), 7.28 (d, *J* = 6.6 Hz, 1H), 5.39 (s, 2H); ^13^C NMR (151 MHz, CDCl_3_) δ 159.0, 136.8, 136.7, 135.1, 131.1, 129.0, 128.9, 128.8, 128.7, 128.3, 128.0, 126.9, 77.4, 77.2, 76.9, 54.9.

*6-Bromo-2-neopentylphthalazin-1(2H)-one (****6b****)*

A microwave vial (5 mL) equipped with a stir bar was charged with 6-bromophthalazin-1(2H)-one (100 mg, 0.444 mmol, 1 eq), KOtBu (150 mg, 1.333 mmol, 3 eq), DMF (1.5 mL) and 2,2'-dimethylbromopropane (0.11 mL, 0.889 mmol, 2 eq). The vial was sealed, the mixture degassed with bubbling N_2_ for 5 min, and the reaction was stirred at 150 °C under microwave irradiation for 15 min until completion, as indicated by TLC. The mixture was partitioned between EtOAc (10 mL) and an aqueous NaOH (1M, 10 mL). The organic fraction was isolated and washed with aqueous NaOH (1M, 2x5 mL). The aqueous fractions were combined, extracted with EtOAc (5 mL), the organic fractions were combined, washed with LiCl (5%, 4x5 mL), brine (5 mL), dried over anhydrous Na_2_SO_4_, filtered and concentrated under reduced pressure. The residue was loaded onto silica and purified by flash column chromatography (SNAP 10g, 10% EtOAc/n-pentane, 7 CV) to afford the title compound as a white solid (50 mg, 0.170 mmol, 38%); R_f_ = 0.43 (5% EtOAc/n-pentane); ^1^H NMR (600 MHz, CDCl_3_) δ 8.29 (dd, *J* = 9.0, 0.7 Hz, 1H), 8.06 (s, 1H), 7.84 (dq, *J* = 3.4, 1.9 Hz, 2H), 4.09 (s, 2H), 1.02 (s, 9H); ^13^C NMR (151 MHz, CDCl_3_) δ 159.7, 135.6, 134.9, 131.0, 129.2, 128.5, 128.1, 126.8, 61.1, 34.3, 28.2.

*6-Bromo-2-((1-(tetrahydro-2H-pyran-2-yl)-1H-pyrazol-3-yl)methyl)phthalazin-1(2H)-one (****6c****)*

An oven-dried round-bottom flask (25 mL) was charged with a stir bar, 6-bromophtalazin-1(2H)-one (675 mg, 3.0, 1 eq) and DMF (5 mL). NaH (360 mg, 9.0 mmol, 3 eq) was added in small portions with vigorous stirring, and the mixture was stirred at ambient temperature for 30 min. **2** (783 mg, 3.9 mmol, 1.3 eq) in DMF (1.5 mL) was added dropwise to the stirred mixture, and the reaction was stirred vigorously for 14 h at ambient temperature until completion as indicated by LC-MS. The mixture was partitioned between EtOAc (30 mL) and ice-cooled aq. NH_4_Cl (50%, 40 mL). The aqueous fraction was isolated, extracted with EtOAc (3 x 10 mL), the organic fractions were combined, washed with LiCl (5%, 5 x 10 mL), brine (10 mL), dried over anhydrous Na_2_SO_4_ and concentrated under reduced pressure to afford the crude. The residue was taken up in DMSO (3 mL) and purified by reverse phase flash column chromatography (SNAP C18 60 g, 20-95% MeCN in H_2_O, 15 CV) to afford the product as a white solid (548 mg, 1.408 mmol, 47%); R_f_ = 0.15 (40% EtOAc in n-pentane); ^1^H NMR (600 MHz, CDCl_3_) δ 8.30 (dt, *J* = 8.4, 0.7 Hz, 1H), 8.08 (d, *J* = 0.7 Hz, 1H), 7.87 – 7.81 (m, 2H), 7.52 (d, *J* = 2.4 Hz, 1H), 6.32 (d, *J* = 2.4 Hz, 1H), 5.43 (s, 2H), 5.40 – 5.34 (m, 1H), 4.08 – 4.03 (m, 1H), 3.67 (td, *J* = 11.5, 2.7 Hz, 1H), 2.04 (ddd, *J* = 6.1, 4.4, 3.3 Hz, 3H), 1.69 – 1.63 (m, 2H), 1.59 – 1.57 (m, 1H); ^13^C NMR (151 MHz, CDCl_3_) δ 158.9, 148.2, 136.6, 135.0, 131.2, 129.0, 128.6, 128.5, 128.2, 126.9, 106.1, 87.9, 68.1, 48.9, 30.8, 25.1, 22.7.

*2-Benzyl-6-(phenylthio)phthalazin-1(2H)-one (****7a****)*

An oven-dried microwave vial (5 mL) equipped with a stir bar was charged with **9a** (50 mg, 0.159 mmol, 1 eq), thiophenol (21 mg, 0.190 mmol, 1.2 eq), Cs_2_CO_3_ (103 mg, 0.317 mmol, 2 eq), Pd_2_(dba)_3_ (7.3 mg, 7.9 µmol, 5 mol%) and Xantphos (9.2 mg, 15.9 µmol, 10 mol%). The vial was sealed, flushed with N_2_, anhydrous DMF (0.86 mL) was added, N_2_ was bubbled through the mixture for 5 min, and the reaction was stirred at 100 °C under microwave irradiation for 2 h until completion, as indicated by LC-MS. The mixture was partitioned between DCM (15 mL) and a saturated aqueous solution of NaHCO_3_ (10 mL). The aqueous fraction was isolated, extracted with DCM (5 mL), the organic fractions were combined, washed with LiCl (2x5 mL), brine (10 mL), dried over anhydrous Na_2_SO_4_, filtered and concentrated under reduced pressure. The residue was loaded onto silica and purified by flash column chromatography (SNAP 5g, 40% EtOAc/n-pentane, 10 CV) to afford the title compound an orange solid (52 mg, 0.151 mmol, 95%); R_f_ = 0.35 (5% EtOAc/n-pentane); ^1^H NMR (600 MHz, DMSO-*d6*) δ 8.36 (s, 1H), 8.17 (d, *J* = 8.5 Hz, 1H), 7.67 (d, *J* = 1.9 Hz, 1H), 7.59 (dd, *J* = 8.5, 1.9 Hz, 1H), 7.58 – 7.55 (m, 2H), 7.53 – 7.49 (m, 3H), 7.33 – 7.27 (m, 5H), 5.30 (s, 2H); ^13^C NMR (151 MHz, DMSO) δ 158.1, 144.4, 137.7, 137.3, 133.6, 131.0, 130.8, 130.2, 130.1, 129.4, 128.5, 127.7, 127.4, 127.0, 124.9, 124.4, 53.6.

*2-Benzyl-6-((7-(dimethylamino)benzo[c][1,2,5]oxadiazol-4-yl)thio)phthalazin-1(2H)-one (****7b****)*

An oven-dried microwave vial (5 mL) equipped with a stir bar was charged with **9a** (50 mg, 0.159 mmol, 1 eq), **5** (37 mg, 0.190 mmol, 1.2 eq), Cs_2_CO_3_ (103 mg, 0.317 mmol, 2 eq), Pd_2_(dba)_3_ (7.3 mg, 7.9 µmol, 5 mol%) and Xantphos (9.2 mg, 15.9 µmol, 10 mol%). The vial was sealed, flushed with N_2_, anhydrous DMF (0.86 mL) was added, N_2_ was bubbled through the mixture for 5 min, and the reaction was stirred at 100 °C under microwave irradiation for 2 h until completion, as indicated by LC-MS. The mixture was partitioned between DCM (15 mL) and a saturated aqueous solution of NaHCO_3_ (10 mL). The aqueous fraction was isolated, extracted with DCM (5 mL), the organic fractions were combined, washed with LiCl (2x5 mL), brine (10 mL), dried over anhydrous Na_2_SO_4_, filtered and concentrated under reduced pressure. The residue was loaded onto silica and purified by flash column chromatography (SNAP 5g, 40% EtOAc/n-pentane, 10 CV) to afford the title compound an orange solid (54 mg, 0.126 mmol, 79%); R_f_ = 0.27 (25% EtOAc/n-pentane); ^1^H NMR (600 MHz, CDCl_3_) δ 8.22 (dd, *J* = 8.6, 0.8 Hz, 1H), 7.94 (d, *J* = 0.7 Hz, 1H), 7.60 (d, *J* = 7.9 Hz, 1H), 7.45 (dd, *J* = 8.5, 1.9 Hz, 1H), 7.42 – 7.39 (m, 2H), 7.32 – 7.28 (m, 3H), 7.26 – 7.23 (m, 1H), 6.10 (d, *J* = 8.0 Hz, 1H), 5.35 (s, 2H), 3.46 (s, 6H); ^13^C NMR (151 MHz, CDCl_3_) δ 159.3, 151.8, 146.0, 145.4, 143.4, 141.7, 137.5, 137.1, 130.3, 129.8, 128.7, 128.6, 127.8, 127.5, 125.5, 122.5, 104.1, 99.6, 54.7, 42.4.

*6-((7-(Dimethylamino)benzo[c][1,2,5]oxadiazol-4-yl)thio)-2-neopentylphthalazin-1(2H)-one (****7c****)*

A microwave vial (5 mL) equipped with a stir bar was charged with **9b** (33 mg, 0.112 mmol, 1 eq), **5** (26 mg, 0.134 mmol, 1.2 eq), Cs_2_CO_3_ (73 mg, 0.224 mmol, 2 eq) and anhydrous DMF (0.6 mL). N_2_ was bubbled through the mixture for 5 min, Pd_2_(dba)_3_ (5.1 mg, 5.6 μmol, 5 mol%) and Xantphos (6.5 mg, 11.2 μmol, 10 mol%) were added, the vial was sealed and purged with N_2_, and the reaction was stirred at 100 °C under microwave irradiation for 1 h until completion, as indicated by TLC. The mixture was partitioned between DCM (10 mL) and a saturated aqueous solution of NaHCO_3_ (10 mL). The aqueous fraction was isolated, extracted with DCM (5 mL), the organic fractions were combined, washed with LiCl (2x5 mL), brine (10 mL), dried over anhydrous Na_2_SO_4_, filtered and concentrated under reduced pressure. The residue was loaded onto silica and purified by flash column chromatography (SNAP 5g, 20% EtOAc/n-pentane, 7 CV) to afford the title compound as a red film (41 mg, 0.100 mmol, 90%); R_f_ = 0.14 (10 % EtOAc/n-pentane); ^1^H NMR (600 MHz, CDCl_3_) δ 8.22 (dd, *J* = 8.5, 0.7 Hz, 1H), 7.92 (d, *J* = 0.7 Hz, 1H), 7.61 (d, *J* = 7.9 Hz, 1H), 7.44 (dd, *J* = 8.5, 1.9 Hz, 1H), 7.32 – 7.27 (m, 1H), 6.10 (d, *J* = 7.9 Hz, 1H), 4.05 (s, 2H), 3.46 (s, 6H), 0.99 (s, 9H); ^13^C NMR (151 MHz, CDCl_3_) δ 159.9, 151.9, 146.0, 145.1, 143.4, 141.7, 136.4, 130.1, 129.6, 127.7, 125.5, 122.3, 104.1, 99.7, 60.9, 42.4, 34.3, 28.2.

*6-((7-(Dimethylamino)benzo[c][1,2,5]oxadiazol-4-yl)thio)-2-((1-(tetrahydro-2H-pyran-2-yl)-1H-pyrazol-3-yl)methyl)phthalazin-1(2H)-one (****7d****)*

An oven-dried microwave vial (5 mL) equipped with a stir bar was charged with **9c** (70 mg, 0.180 mmol, 1 eq), **5** (42 mg, 0.216 mmol, 1.2 eq), Pd_2_(dba)_3_ (8.2 mg, 9 µmol, 5 mol%), Xantphos (10.4 mg, 18 µmol, 10 mol%) and Cs_2_CO_3_ (117 mg, 0.360 mmol, 2 eq). The vial was sealed, flushed with Ar for 5 min, anhydrous DMF (1 mL) was added and the reaction was stirred at 100 °C under microwave irradiation for 2 h until completion, as indicated by TLC. The mixture was partitioned between DCM (10 mL) and a saturated aqueous solution of NH_4_Cl (20 mL). The aqueous fraction was isolated and extracted with DCM (3x5 mL). The organic fractions were combined, washed with LiCl (5%, 3x10 mL), brine (20 mL), dried over anhydrous Na_2_SO_4_, filtered and concentrated under reduced pressure. The residue was loaded on silica and purified by flash column chromatography (SNAP 10 g, 4% MeOH/DCM, 6 CV) to afford the title compound as an orange solid (88 mg, 0.175 mmol, 97%); R_f_ = 0.52 (5% MeOH/DCM); ^1^H NMR (600 MHz, CDCl_3_) δ 8.23 (d, *J* = 8.5 Hz, 1H), 7.94 (d, *J* = 0.7 Hz, 1H), 7.60 (d, *J* = 7.9 Hz, 1H), 7.50 (d, *J* = 2.4 Hz, 1H), 7.45 (dd, *J* = 8.5, 1.8 Hz, 1H), 7.28 (dd, *J* = 1.8, 0.5 Hz, 1H), 6.28 (d, *J* = 2.5 Hz, 1H), 6.10 (d, *J* = 7.9 Hz, 1H), 5.40 (s, 2H), 5.35 (dd, *J* = 9.1, 3.3 Hz, 1H), 4.06 – 4.00 (m, 1H), 3.66 (td, *J* = 11.4, 2.6 Hz, 1H), 3.46 (s, 6H), 2.06 – 1.96 (m, 3H), 1.69 – 1.63 (m, 2H), 1.59 – 1.53 (m, 1H); ^13^C NMR (151 MHz, CDCl_3_) δ 159.2, 151.9, 148.6, 146.0, 145.3, 143.4, 141.8, 137.4, 130.4, 129.7, 128.4, 127.5, 125.5, 122.5, 105.9, 104.1, 99.7, 87.8, 68.0, 48.7, 42.4, 30.7, 25.1, 22.7.

*6-((7-(Dimethylamino)benzo[c][1,2,5]oxadiazol-4-yl)sulfonyl)-2-((1-(tetrahydro-2H-pyran-2-yl)-1H-pyrazol-3-yl)methyl)phthalazin-1(2H)-one (****8a****)*

A round-bottom flask (25 mL) equipped with a stir bar was charged with **10d** (65 mg, 0.129 mmol, 1 eq), Na_2_WO_4_·2 H_2_O (13 mg, 0.039 mmol, 0.3 eq), DCM (4 mL) and MeOH (4 mL). The flask was cooled in an ice bath for 10 min, H_2_O_2_ (30%, 0.53 mL, 5.163 mmol, 40 eq) was added dropwise and the reaction was stirred at ambient temperature for 22 h until completion, as indicated by TLC. The mixture was partitioned between H_2_O (20 mL) and DCM (10 mL). The organic fraction was isolated, the aqueous fraction was extracted with DCM (3x5 mL), the combined organic fractions were dried over anhydrous Na_2_SO_4_, filtered and concentrated under reduced pressure. The residue was loaded on silica and purified by flash column chromatography (SNAP 10 g, 12.5/37.5/50% EtOH/EtOAc/n-pentane) to afford the title compound as a bright yellow solid (55 mg, 0.103 mmol, 80%); R_f_ = 0.22 (1:3:6 EtOH/EtOAc/n-pentane); ^1^H NMR (700 MHz, CDCl_3_) δ 8.56 (dd, *J* = 1.7, 0.6 Hz, 1H), 8.49 (d, *J* = 8.5 Hz, 1H), 8.32 (dd, *J* = 8.5, 1.7 Hz, 1H), 8.26 (d, *J* = 0.7 Hz, 1H), 8.11 (d, *J* = 8.5 Hz, 1H), 7.50 (d, *J* = 2.5 Hz, 1H), 6.29 (d, *J* = 2.4 Hz, 1H), 6.09 (d, *J* = 8.5 Hz, 1H), 5.42 (s, 2H), 5.37 – 5.33 (m, 1H), 4.05 – 4.01 (m, 1H), 3.65 (td, *J* = 11.5, 2.6 Hz, 1H), 3.50 (s, 6H), 2.04 – 1.97 (m, 3H), 1.66 – 1.64 (m, 2H), 1.60 – 1.55 (m, 1H); ^13^C NMR (176 MHz, CDCl_3_) δ 158.3, 148.0, 146.3, 146.0, 145.1, 144.8, 138.9, 137.4, 130.7, 129.8, 129.5, 128.5, 128.4, 126.6, 109.5, 106.0, 101.5, 87.8, 68.1, 49.0, 43.0, 30.8, 25.1, 22.7.

*2-Benzyl-6-(phenylsulfonyl)phthalazin-1(2H)-one (****I****)*

A glass vial (5 mL) equipped with a stir bar was charged with a **2a** (50 mg, 0.145 mmol, 1 eq) and DMF (2 mL). The vial was sealed and cooled in an ice bath for 5 min. Oxone (538 mg, 1.164 mmol, 10 eq) was added and the reaction was stirred at ambient temperature for 3 h until completion, as indicated by LC-MS. The mixture was partitioned between EtOAc (20 mL) and a saturated aqueous solution of NaHCO_3_ (15 mL). The aqueous fraction was isolated, extracted with EtOAc (2x5 mL), the organic fractions were combined, washed with LiCl (5%, 4x5 mL), brine (10 mL), dried over anhydrous Na_2_SO_4_, filtered and concentrated under reduced pressure. The residue was loaded onto silica and purified by flash column chromatography (SNAP 10 g, 2% MeOH/DCM, 15 CV) to afford the title compound as a yellow solid (15 mg, 0.040 mmol, 28%); R_f_ = 0.58 (10% MeCN/DCM); ^1^H NMR (600 MHz, CDCl_3_) δ 8.53 (dt, *J* = 8.4, 0.7 Hz, 1H), 8.32 (dd, *J* = 1.7, 0.6 Hz, 1H), 8.24 (d, *J* = 0.7 Hz, 1H), 8.17 (dd, *J* = 8.4, 1.8 Hz, 1H), 8.01 – 7.93 (m, 2H), 7.64 – 7.59 (m, 1H), 7.56 – 7.52 (m, 2H), 7.46 – 7.41 (m, 2H), 7.33 – 7.29 (m, 2H), 7.28 – 7.26 (m, 1H), 5.38 (s, 2H); ^13^C NMR (151 MHz, CDCl_3_) δ 158.3, 146.4, 140.3, 137.2, 136.4, 134.2, 130.8, 130.0, 129.8, 129.5, 128.9, 128.8, 128.8, 128.1, 126.0, 55.2; HRMS (m/z): [M]+ calcd. for C_21_H_16_N_2_O_3_S, 376.08816; found, 376.0882.

*2-Benzyl-6-((7-(dimethylamino)benzo[c][1,2,5]oxadiazol-4-yl)sulfonyl)phthalazin-1(2H)-one (****II****)*

A glass vial (5 mL) equipped with a stir bar was charged **2b** (50 mg, 0.116 mmol, 1 eq) and DMF (2 mL). The vial was sealed and cooled in an ice bath for 5 min. Oxone (538 mg, 1.164 mmol, 10 eq) was added and the reaction was stirred at ambient temperature for 5 h until completion, as indicated by LC-MS. The mixture was partitioned between EtOAc (20 mL) and a saturated aqueous solution of NaHCO_3_ (15 mL). The aqueous fraction was isolated, extracted with EtOAc (2x5 mL), the organic fractions were combined, washed with LiCl (5%, 4x5 mL), brine (10 mL), dried over anhydrous Na_2_SO_4_, filtered and concentrated under reduced pressure. The residue was loaded onto silica and purified by flash column chromatography (SNAP 10 g, 3-12% MeCN/DCM, 20 CV) to afford the title compound as a yellow solid (21 mg, 0.046 mmol, 39%); R_f_ = 0.44 (10 % MeCN/DCM); ^1^H NMR (600 MHz, CDCl_3_) δ 8.58 (d, *J* = 1.7 Hz, 1H), 8.49 (d, *J* = 8.4 Hz, 1H), 8.32 (dd, *J* = 8.4, 1.8 Hz, 1H), 8.27 (s, 1H), 8.12 (d, *J* = 8.4 Hz, 1H), 7.45 – 7.40 (m, 2H), 7.33 – 7.27 (m, 2H), 7.25 (s, 3H), 6.09 (d, *J* = 8.5 Hz, 1H), 5.38 (s, 2H), 3.54 (s, 6H); ^13^C NMR (151 MHz, CDCl_3_) δ 158.4, 146.3, 146.0, 145.1, 144.8, 138.9, 137.6, 136.5, 130.7, 129.8, 129.6, 128.8, 128.7, 128.4, 128.1, 126.7, 109.5, 101.5, 55.1, 43.0; HRMS (m/z): [M]+ calcd. for C_23_H_19_N_5_O_4_S, 461.11578; found, 461.1150.

*6-((7-(Dimethylamino)benzo[c][1,2,5]oxadiazol-4-yl)sulfonyl)-2-neopentylphthalazin-1(2H)-one (****III****)*

A round-bottom flask (10 mL) equipped with a stir bar was charged with **2c** (35 mg, 0.086 mmol, 1 eq) and CHCl_3_ (5 mL). The flask was cooled in an ice bath for 5 min, mCPBA (48 mg, 0.214 mmol, 2.5 eq) was added in one portion, and the reaction was stirred in the ice bath for 2 h until completion, as indicated by TLC. The mixture was diluted with EtOAc (20 mL), washed with a saturated aqueous solution of Na_2_CO_3_ (2x5 mL), brine (5 mL), dried over anhydrous Na_2_SO_4_, filtered, and concentrated under reduced pressure. The residue was loaded onto silica and purified by flash column chromatography (SNAP 10g, 3% MeOH/DCM, 10 CV) to afford the title compound as a yellow solid (26 mg, 0.059 mmol, 69%); R_f_ = 0.67 (5% MeOH/DCM); ^1^H NMR (600 MHz, CDCl_3_) δ 8.58 (d, *J* = 1.8 Hz, 1H), 8.49 (d, *J* = 8.4 Hz, 1H), 8.32 (dd, *J* = 8.5, 1.8 Hz, 1H), 8.25 (d, *J* = 0.7 Hz, 1H), 8.13 (d, *J* = 8.5 Hz, 1H), 6.09 (d, *J* = 8.5 Hz, 1H), 4.08 (s, 2H), 3.51 (s, 6H), 1.00 (s, 9H); ^13^C NMR (151 MHz, CDCl_3_) δ 159.1, 146.4, 145.9, 145.1, 144.8, 138.9, 136.5, 130.6, 129.6, 129.4, 128.6, 126.5, 109.6, 101.5, 61.2, 43.0, 34.4, 28.2; HRMS (m/z): [M]+ calcd. for C_21_H_23_N_5_O_4_S, 441.14708; found, 441.1463.

*2-((1H-Pyrazol-3-yl)methyl)-6-((7-(dimethylamino)benzo[c][1,2,5]oxadiazol-4-yl)sulfonyl)phthalazin -1(2H)-one (****IV****)*

A round-bottom flask (10 mL) equipped with a stir bar was charged with **2e** (50 mg, 0.093 mmol, 1 eq) and DCM (4 mL). The flask was sealed and cooled in an ice bath for 10 min. MeSO_4_H (0.50 mL, 7.705 mmol, 80 eq) was added dropwise with strong stirring, and the reaction was stirred at ambient temperature for 2 h until completion as indicated by TLC. The mixture was poured over ice-cooled aqueous NaOH (1 M, 25 mL). The organic fraction was isolated, and the aqueous fraction was extracted with DCM (5x5 mL). The organic fractions were combined, dried over anhydrous Na_2_SO_4_, filtered, and concentrated under reduced pressure. The crude was purified by reverse phase flash column chromatography (SNAP 25 g, 5-95% MeCN/H2O, 15 CV) to afford the title compound as a bright yellow solid (33 mg, 0.073 mmol, 78%); R_f_ = 0.28 (5% MeOH/DCM); ^1^H NMR (600 MHz, DMSO) δ 8.70 – 8.52 (m, 2H), 8.40 (d, *J* = 8.5 Hz, 1H), 8.32 (dd, *J* = 8.5, 1.9 Hz, 1H), 8.14 (d, *J* = 8.6 Hz, 1H), 7.59 (s, 1H), 6.35 (d, *J* = 8.7 Hz, 1H), 6.15 (s, 1H), 5.28 (s, 2H), 3.46 (s, 6H); ^13^C NMR (151 MHz, DMSO) δ 157.3, 146.0, 145.7, 145.6, 144.8, 144.7, 139.5, 137.7, 129.7, 129.6, 129.2, 127.7, 126.3, 106.2, 104.3, 103.8, 101.9, 47.8, 42.6; HRMS (m/z): [M]+ calcd. for C_20_H_17_N_7_O_4_S, 451.10627; found, 451.1056.

# **Supplementary figures and tables**


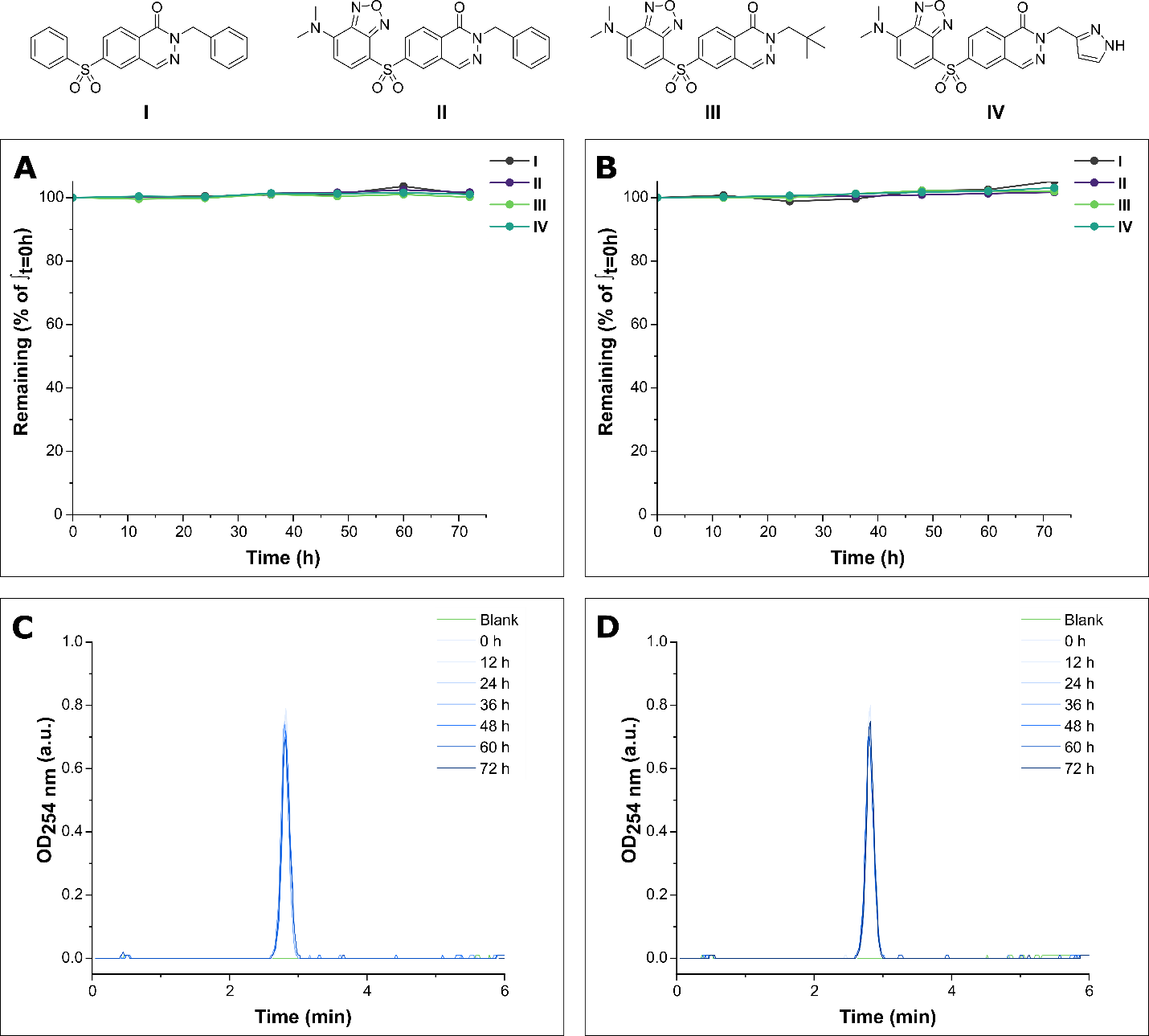


**Figure S1.** Buffer stability measurement of compounds **I**-**IV** in serum-supplemented (1% FBS) Opti-MEM buffer and serum-free Tris-HCl buffer. Stock solutions of compounds in DMSO were diluted to 100 µM (1% DMSO) in the respective buffers, and the samples were incubated at 37 °C and monitored continuously with HPLC for 72 h. For each time point, sample purities were determined by normalizing the integrated peak area against the peak area of the first injection (t = 0 h) of each sample. The chemical structures of compounds **I**-**IV** are displayed in panels A-D. (A) Stability curves of compounds **I**-**IV** incubated in serum-supplemented (1% FBS) Opti-MEM buffer. (B) Stability curves of compounds **I**-**IV** incubated in serum-free Tris-HCl buffer. (C) Superimposed HPLC chromatograms of injections from **IV** incubated in serum-supplemented (1% FBS) Opti-MEM buffer. Injections were made after 0, 12, 24, 36, 48, 60, and 72 h of incubation time. (D) Superimposed HPLC chromatograms of injections from **IV** incubated in serum-free Tris-HCl buffer. Injections were made after 0, 12, 24, 36, 48, 60, and 72 h of incubation time. OD_254 nm_ = optical density at 254 nm.


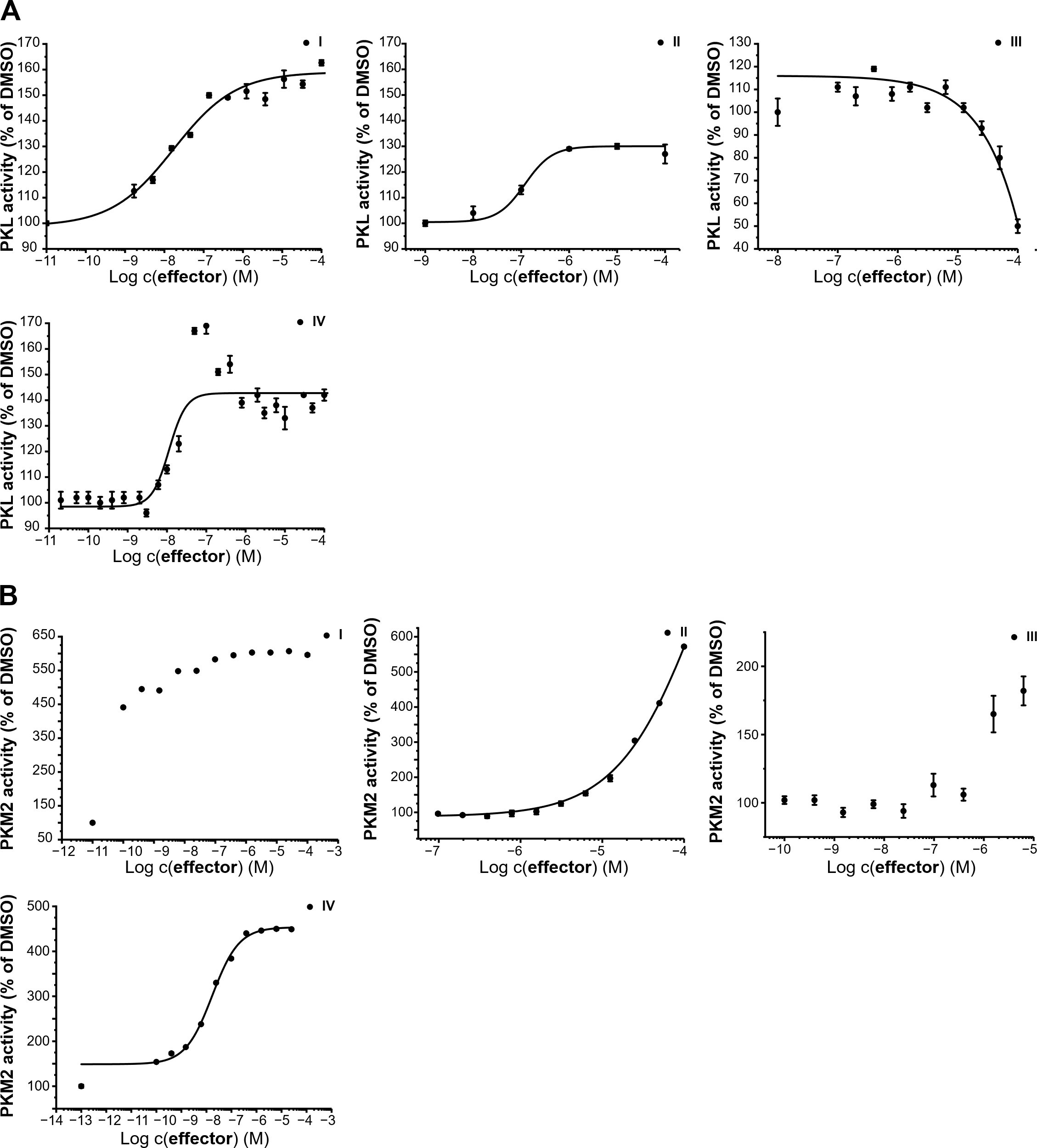


**Figure S2.** Effects of ligands **I**-**IV** on recombinant PK activities. PKL or PKM2 (10 nM) were pre-incubated with the compounds (0-100 µM) for 15 min following the addition of PEP (0.1 mM) and ADP (0.2 mM). The reactions were terminated by the addition of Kinase Glo-MAX reagent after 10 min, and the luminescence from each reaction was quantified using a plate reader. The activity values are expressed as a percentage of PK activity of the DMSO control. The potencies were calculated by best fit of the Hill equation to the luminescence data.^[2]^ (A) PKL titrations. (B) PKM2 titrations. Error bars represent the ±SE of replicates (*n = 3*).

**
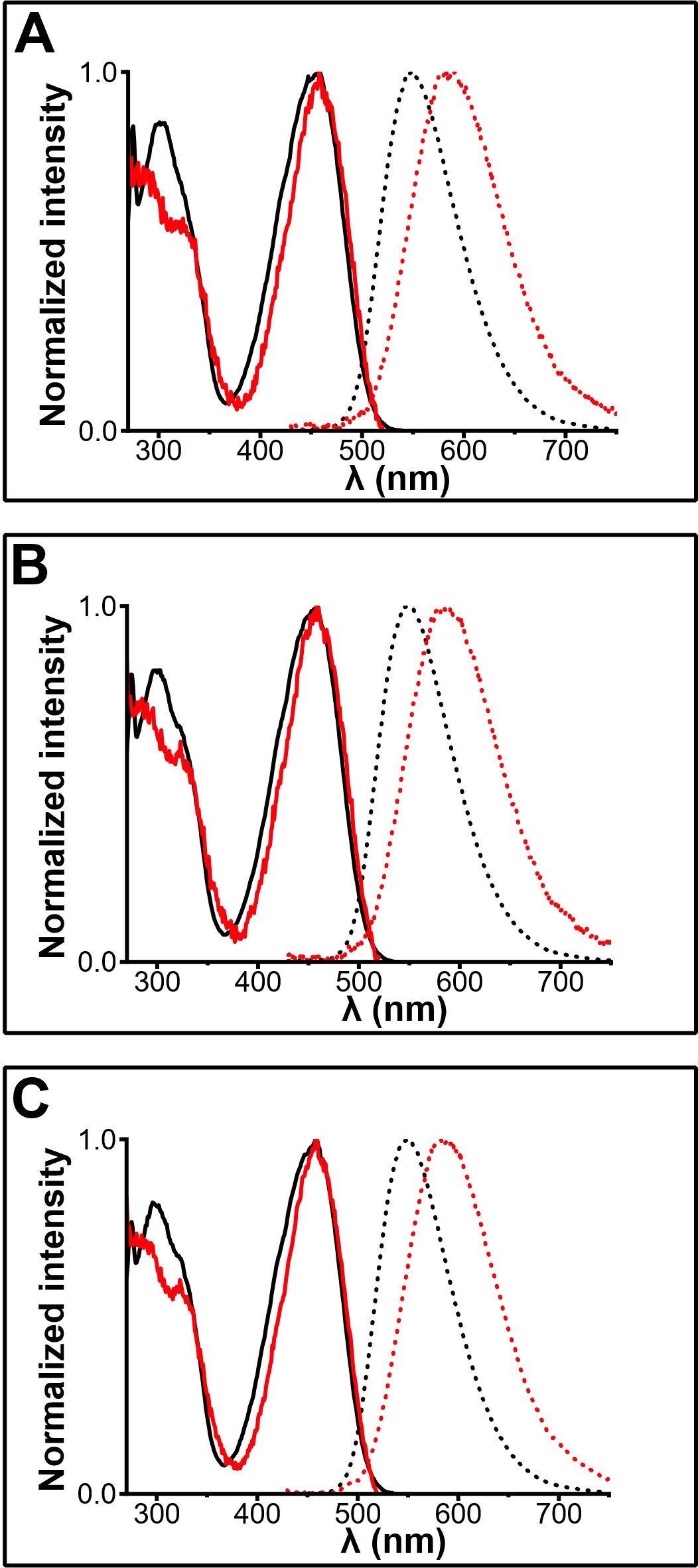
**

**Figure S3.** Fluorescence excitation and emission spectra of tracers **II**, **III,** and **IV** in MeCN and Tris-HCl buffer. Emission (Em) spectra are drawn as dotted lines, and excitation spectra (Ex) are drawn as solid lines. The traces are coloured according to solvent: MeCN (black), Tris-HCl (red). (A) Fluorescence spectra of compounds **II**. (B) Fluorescence spectra of compounds **III**. (C) Fluorescence spectra of compounds **IV**.

**Table S1.** Summary of spectroscopic properties of tracers **II-IV** in buffered media and MeCN. Determination of fluorescence quantum yields (*Φ*_f_) was done by excitation at 420 nm.

|  | *Tris-HCl buffer* | | |
| --- | --- | --- | --- |
| **Compound** | **λ_ex_ (nM)**^[a]^ | ***λ*_em_ (nM)**^[b]^ | ***Φ*_f_ (%)** |
| **II** | 458 | 591 | N/D^[c]^ |
| **III** | 456 | 593 | N/D^[c]^ |
| **IV** | 458 | 590 | 1.5 |
|  | *MeCN* | | |
| **II** | 458 | 550 | 5.6 |
| **III** | 459 | 548 | 5.3 |
| **IV** | 458 | 552 | 5.3 |

[a] Excitation maxima [b] Emission maxima [c] Not determined





**Figure S4.** Fluorescence indication titrations of tracer **IV** against PKL_wt_ in the presence (red) and absence (black) of 10 µM FBP. Data is presented as mean ± SE (*n = 3*), error bars represent ±SE.


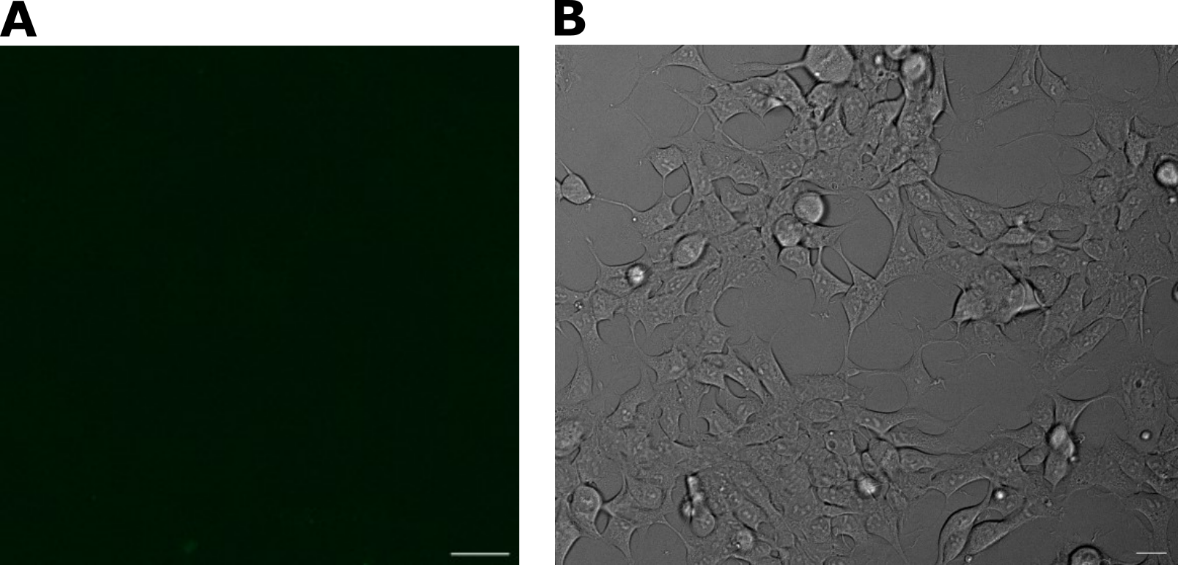


**Figure S5.** Live-cell imaging of HEK293 cells following DMSO addition. (A) Fluorescence emission upon excitation at 488 nm during the cellular uptake of DMSO. Mean fluorescence emission (*λ*_ex_ = 488 nm) was recorded immediately after the addition of DMSO to HEK293 cells. (B) Brightfield (BF) images display the cells after the incubation period of 5 min. Scale bars: 20 µm.


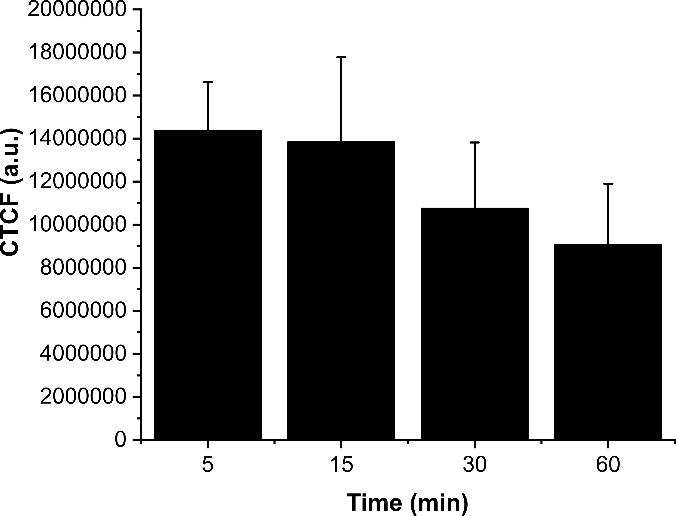


**Figure S6.** Intracellular fluorescence of tracer **IV** in live HEK293 cells assessed by CTCF analysis. Surface-adhered HEK293 cells were treated with **IV** (1 µM), and Z-stack images were captured after 5, 15, 30, and 60 min of incubation (37 °C, 5% CO_2_) across a physical depth of ~22 μm. For each image, CTCF was determined by drawing an equal number (*n=*3) of regions of interest (ROIs) around fluorescent cells and background regions in the corresponding images in Figure 3B. Corrected Total Cell Fluorescence (CTCF) was calculated by using ImageJ, and according to the following equation: *CTCF = Integrated Density – (Area of selected cell × Mean fluorescence of background readings)*. For the calculation of CTCF, three-dimensional images or Z-stacks were reduced to bidimensional images by the arithmetic sum of the fluorescence intensity of the different slices. CTCF was expressed as the average of measuring various cells. As for the background readings, an equal number of regions of interest (ROIs) were drawn in the corresponding pictures. Data is presented as mean ±SE (*n=3*), error bars represent ±SE.


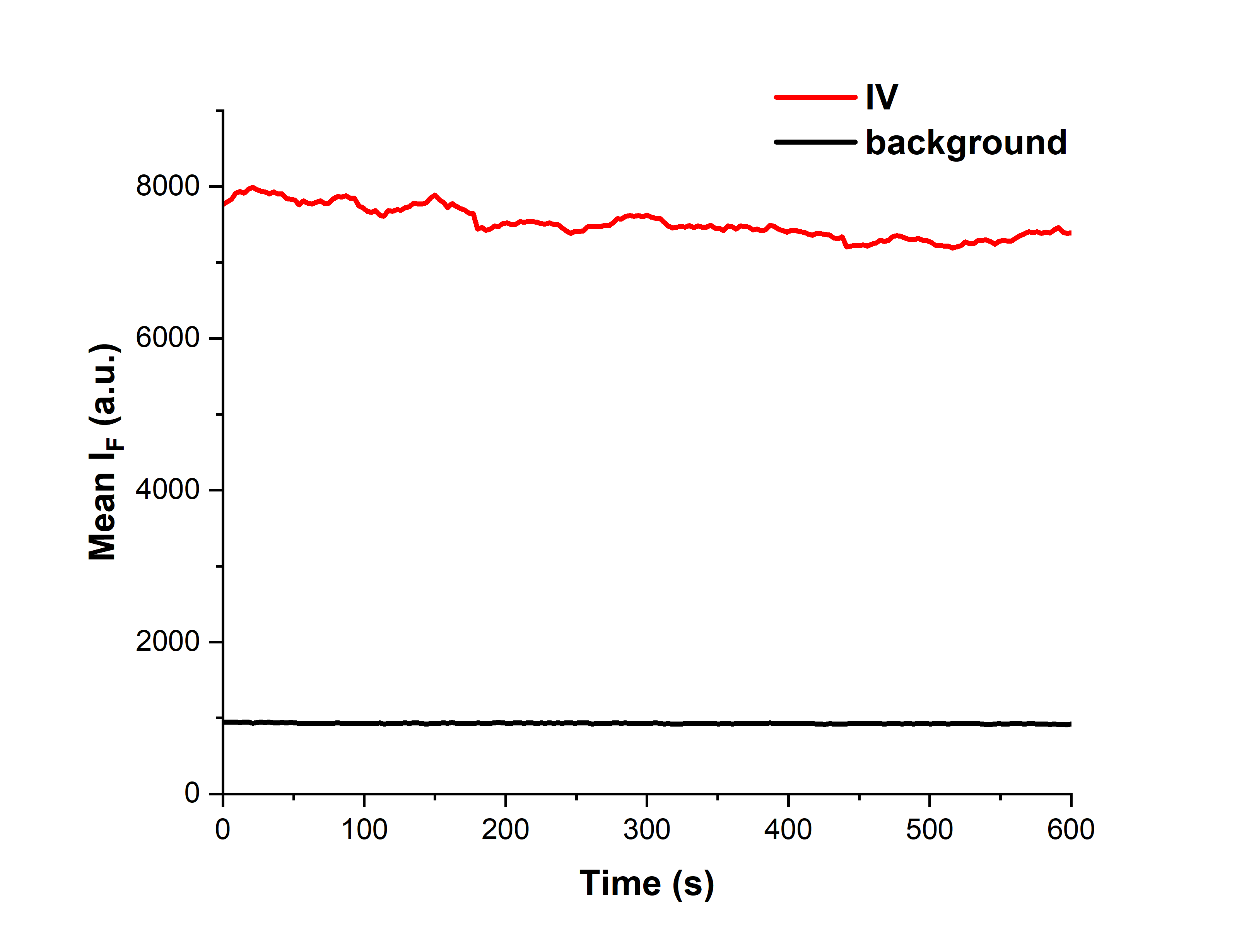


**Figure S7.** Photobleaching curve of compound **IV** (1 µM) in HEK293 cells under continuous [laser irradiation.](https://www.sciencedirect.com/topics/engineering/laser-irradiation) HEK293 cells were exposed to continuous excitation at 488 nm for 10 minutes using the same imaging settings as applied throughout this study. Integrated fluorescence intensity was recorded over time from an identical region of interest (ROI) across the time-lapse images to assess photostability.


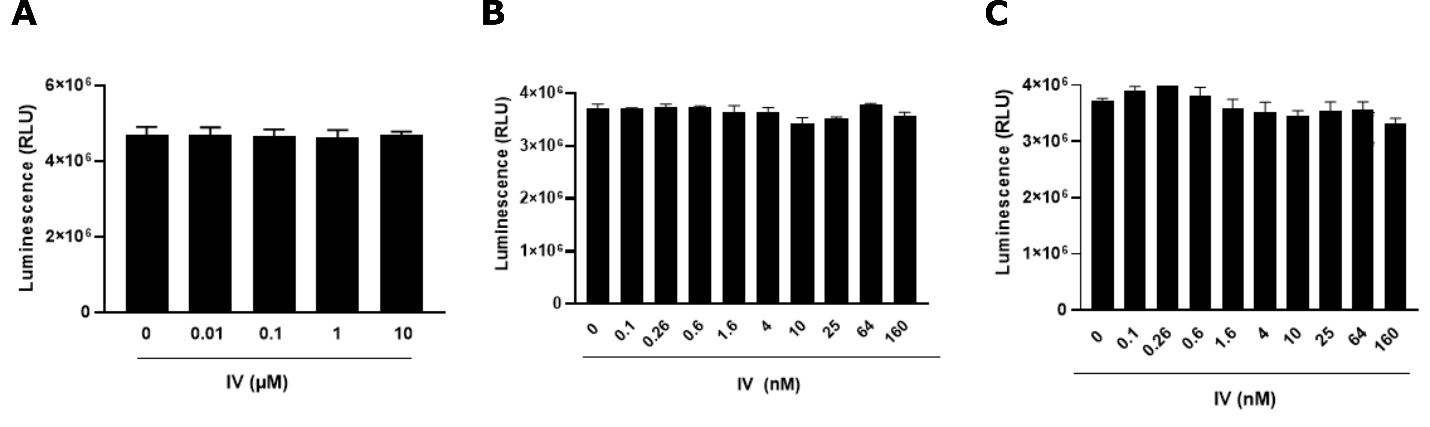


**Figure S8.** Cell viability assay (CellTiter-Glo 2.0) of HEK293 cells treated with different concentrations of tracer **IV.** (A) Cell viability assay performed on HEK293 cells incubated for 2 h with different concentrations of tracer **IV** or DMSO (0 nM **IV**) in FluoroBrite™ DMEM complete medium. Results are reported as relative luminescence values (mean ± SE, n=4, One Way ANOVA followed by Dunnett´s multiple comparison test). (B) Cell viability assay performed on HEK293 cells transfected with PKL_Nluc_ plasmid and incubated for 2 h with different concentrations of tracer **IV** or DMSO (0 nM **IV**) in Opti-MEM™ (1% FBS) medium. Results are reported as relative luminescence values (mean ± SE, n=4, One Way ANOVA followed by Dunnett´s multiple comparison test). (C) Cell viability assay performed on HEK293 cells transfected with Nluc-plasmid and incubated for 2 h with different concentrations of tracer **IV** or DMSO (0 nM **IV**) in Opti-MEM™ (1% FBS) medium. Results are reported as relative luminescence values (mean ± SE, n=4, One Way ANOVA followed by Dunnett´s multiple comparison test).


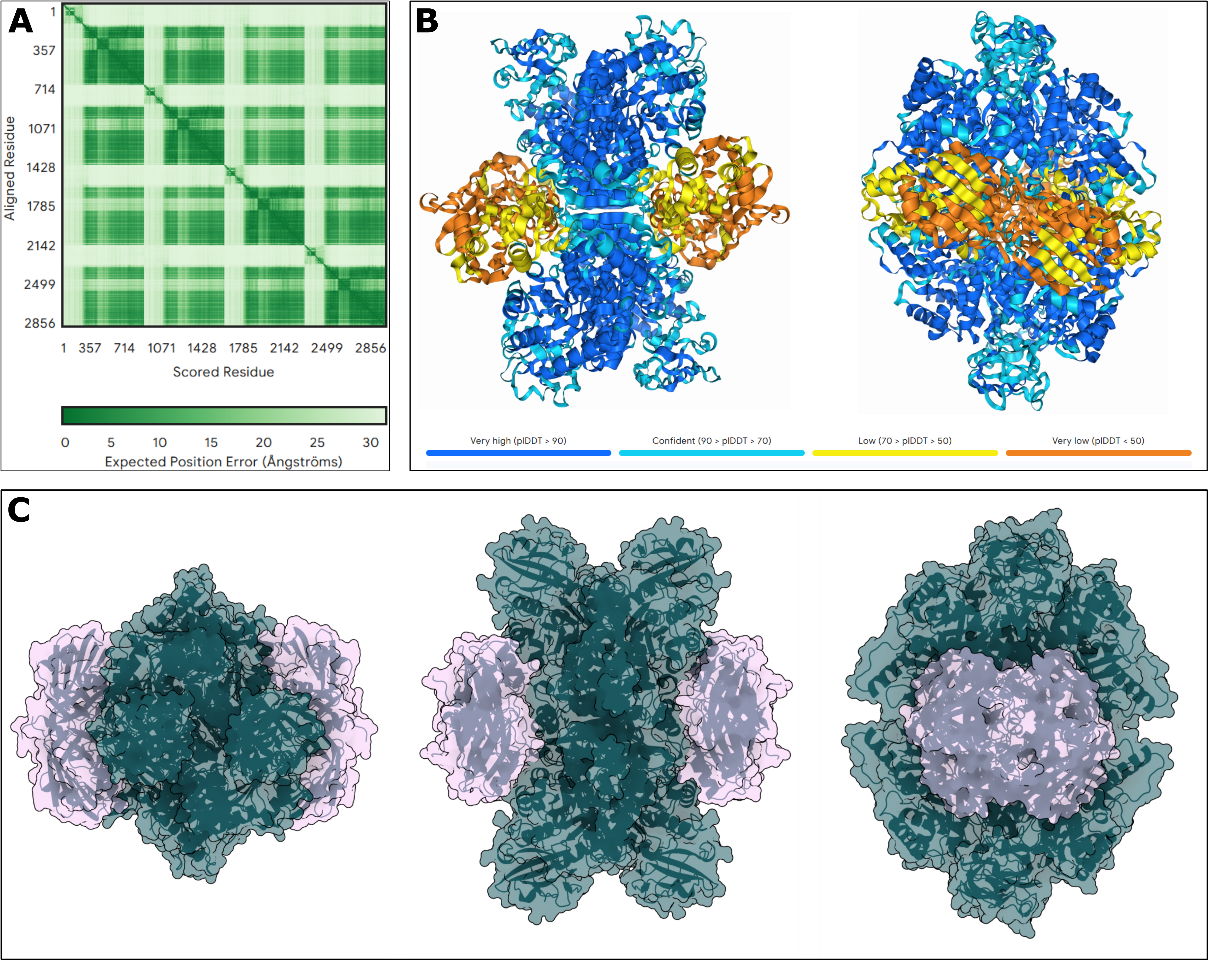


**Figure S9.** Illustrations and confidence scores of the highest confidence AlphaFold-generated PKL_Nluc_ structure. (A) Predicted aligned error matrix of the structure with the highest confidence. Darker colours indicate lower positional error in the relative position of α-carbon atoms. The light streaks correspond to Nluc and the N-terminal linker between the two proteins. (B) Colour map of the distance difference test (pDDT) scores mapped onto the protein structure, seen from two different angles. (C) Transparent surface- and cartoon-representation of the PKL_Nluc_ fusion protein, seen from three different angles.

**
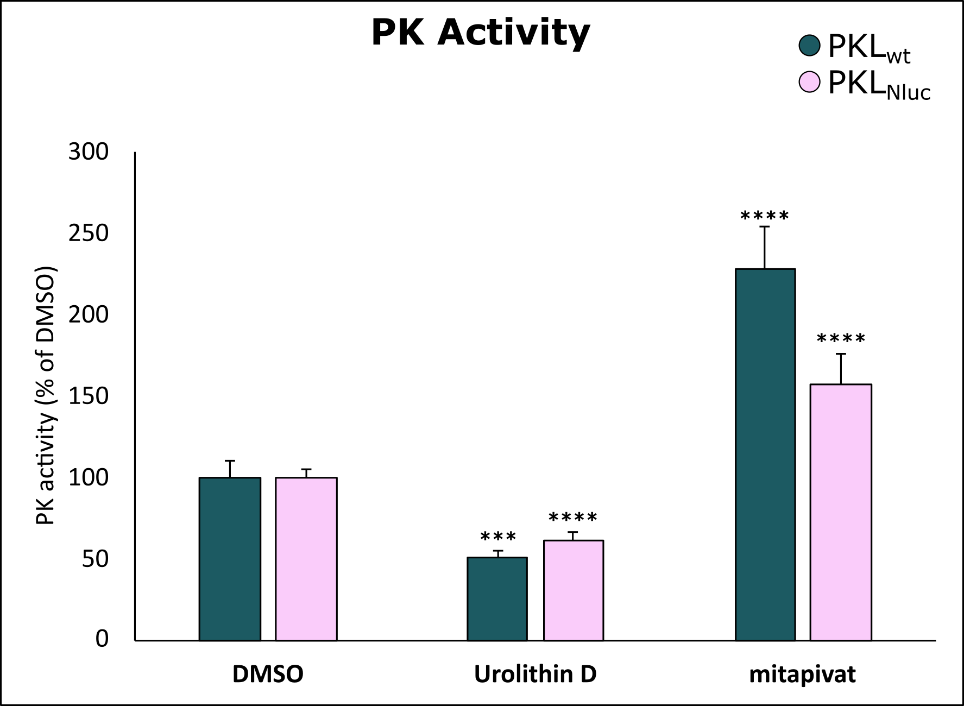
**

**Figure S10.** Relative enzymatic activities of PKL_Nluc_ compared to PKL_wt_ as determined by Kinase Glo-MAX assay. Reported allosteric inhibitor Urolithin D^[3]^ and allosteric activator mitapivat^[4]^ were used as controls to compare the allosteric regulation of PKL_wt_ and PKL_Nluc_. The enzymes (10 nM) were pre-incubated with compounds (10 µM) or DMSO for 15 min following addition of PEP (0.1 mM) and ADP (0.2 mM). The reactions were terminated by addition of Kinase Glo-MAX reagent after 10 min and the luminescence from each reaction was quantified using a plate reader. The activity levels of the samples are displayed as mean ±SE (*n = 3*), error bars represent SE, ^***^p<0.001, ^****^p<0.0001; One Way ANOVA followed by Dunnett´s multiple comparison test. The comparison was done versus DMSO control for respective PKL recombinant.


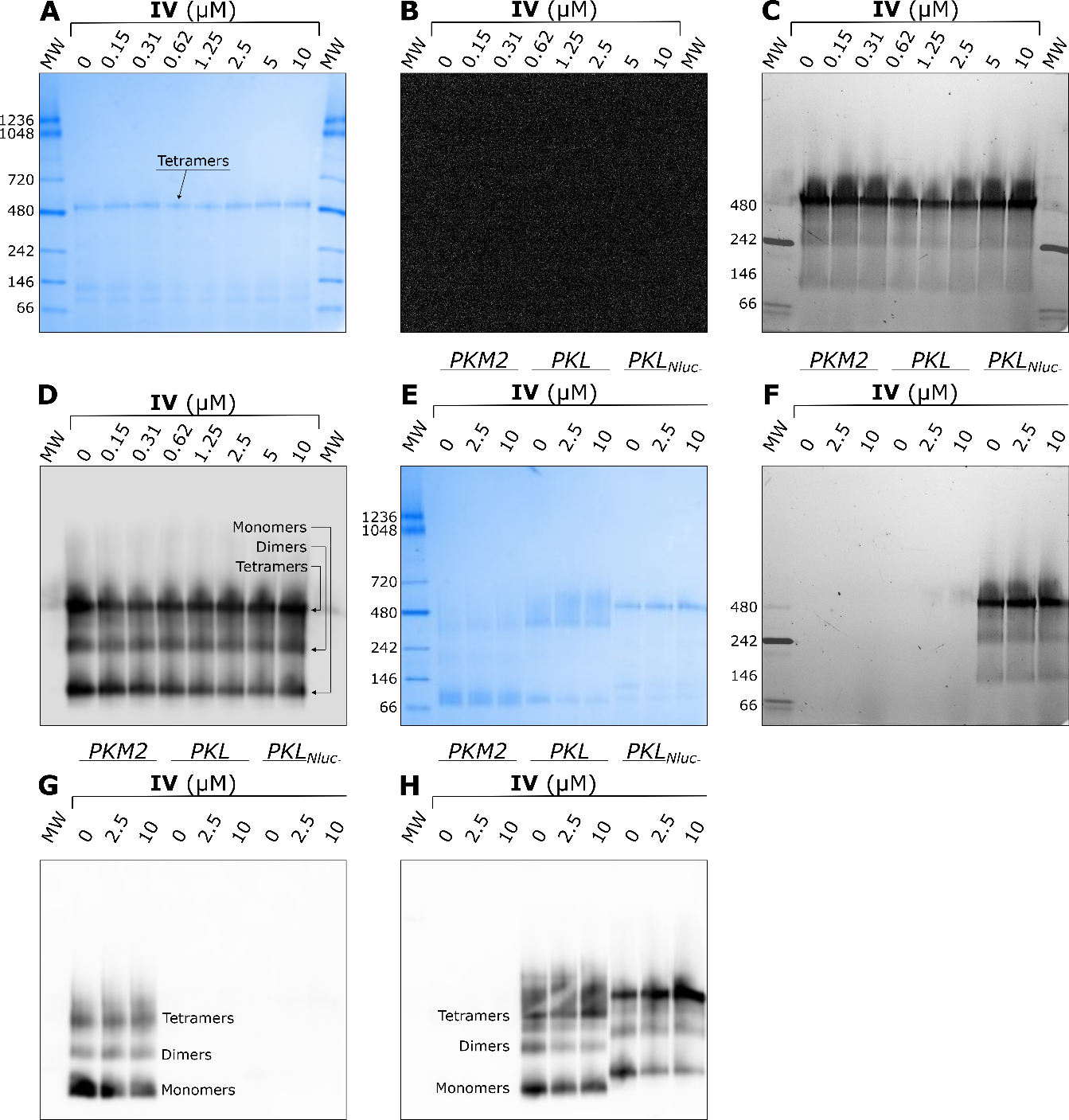


**Figure S11.** Native PAGE experiments with recombinant PK enzymes incubated with different concentrations of tracer **IV**. (A) Colloidal blue gel staining of Native PAGE gel (3-12% Bis-Tris) loaded with recombinant PKL_Nluc_ after incubation for 1 h with different concentrations of tracer **IV**. MW size markers are shown on the far left and right sides of the gel. (B) In-gel luminescence detection of PKL_Nluc_ before addition of NanoLuc substrate (background). (C) Superimposed images from panels A and B from Figure 3 in the main text. (D) Western blot of PKL_Nluc_ recombinant protein after incubation with **IV** (as in Figure 3A), separation of proteins by Native PAGE, and transfer to PVDF membrane, followed by incubation with primary antibodies anti-rabbit PKL, secondary donkey anti-rabbit-HRP and chemiluminescence detection. (E) Colloidal blue staining of Native PAGE (3-12% Bis-Tris) gel loaded with recombinant PKM2, PKL_wt_ and PKL_Nluc_ after incubation for 1 h with different concentrations of tracer **IV**, and separation. MW size markers and annotations for panels E-H are shown on the far left of the gel. (F) Superimposed images from panels C and D from Figure 3 in the main text. (G) Western blot of PKM2, PKL_wt_ and PKL_Nluc_ recombinant proteins after incubation with **IV** as in Figure 3C main text, separation of proteins by Native PAGE, and transfer to PVDF membrane, followed by incubation with primary antibodies anti-rabbit PKM2, secondary donkey anti-rabbit-HRP and chemiluminescence detection. (H) Western blot of PKL and PKL_Nluc_ recombinant proteins after incubation with **IV** as in Figure 3A, main text, separation of proteins by Native PAGE and transfer to PVDF membrane, followed by incubation with primary antibodies anti-rabbit PKL, secondary donkey anti-rabbit-HRP and chemiluminescence detection.


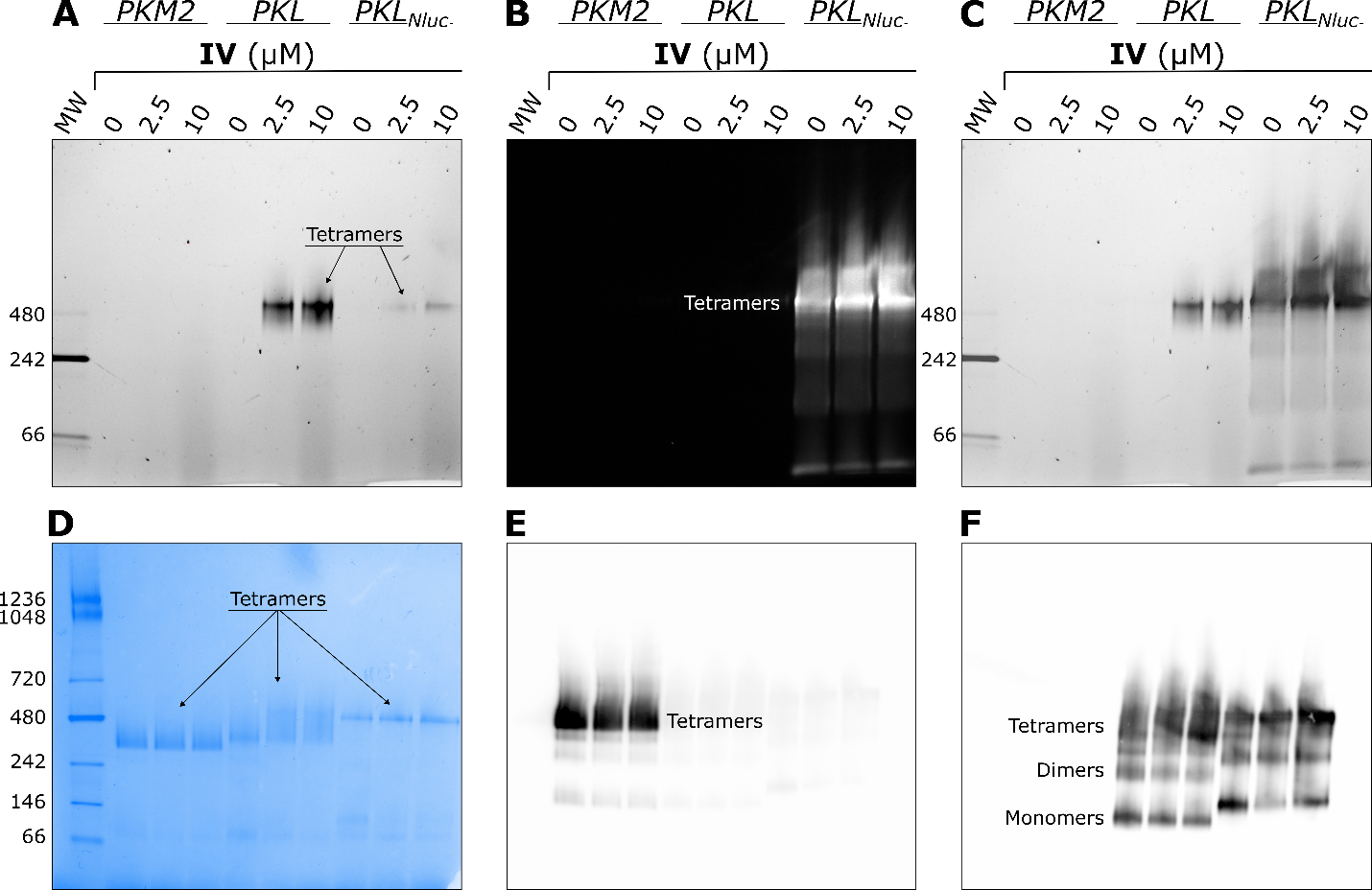


**Figure S12.** Native PAGE (3-12 % Bis-Tris gel) experiments with recombinant PK enzymes incubated with different concentrations of tracer **IV**. (A) Binding of tracer **IV** to PKM2, PKL and PKL_Nluc_ recombinant proteins as assessed by in-gel fluorescent detection of the labelled proteins after incubation for 1 h at rt with different concentrations of **IV** and FBP (10 µM)**,** followed by separation. (B) In-gel luminescence detection of PKL_Nluc_ after addition of Nluc substrate to the gel from panel A**.** (C) Superimposed images from panels A and B. (D) Colloidal blue staining of Native PAGE gel loaded with recombinant PKM2, PKL_wt_ and PKL_Nluc_ after incubation for 1 h with different concentrations of tracer **IV** and FBP (10 µM), followed by separation. (E) Chemiluminescence detection of PKL and PKL_Nluc_ recombinant proteins after incubation with **IV** and FBP (10 µM). The membrane was immunoassayed with anti-PKM2 antibodies. (F) Chemiluminescence detection of PKL and PKL_Nluc_ recombinant proteins after incubation with **IV** and FBP (10 µM). The membrane was immunoassayed with anti-PKL antibodies.

**
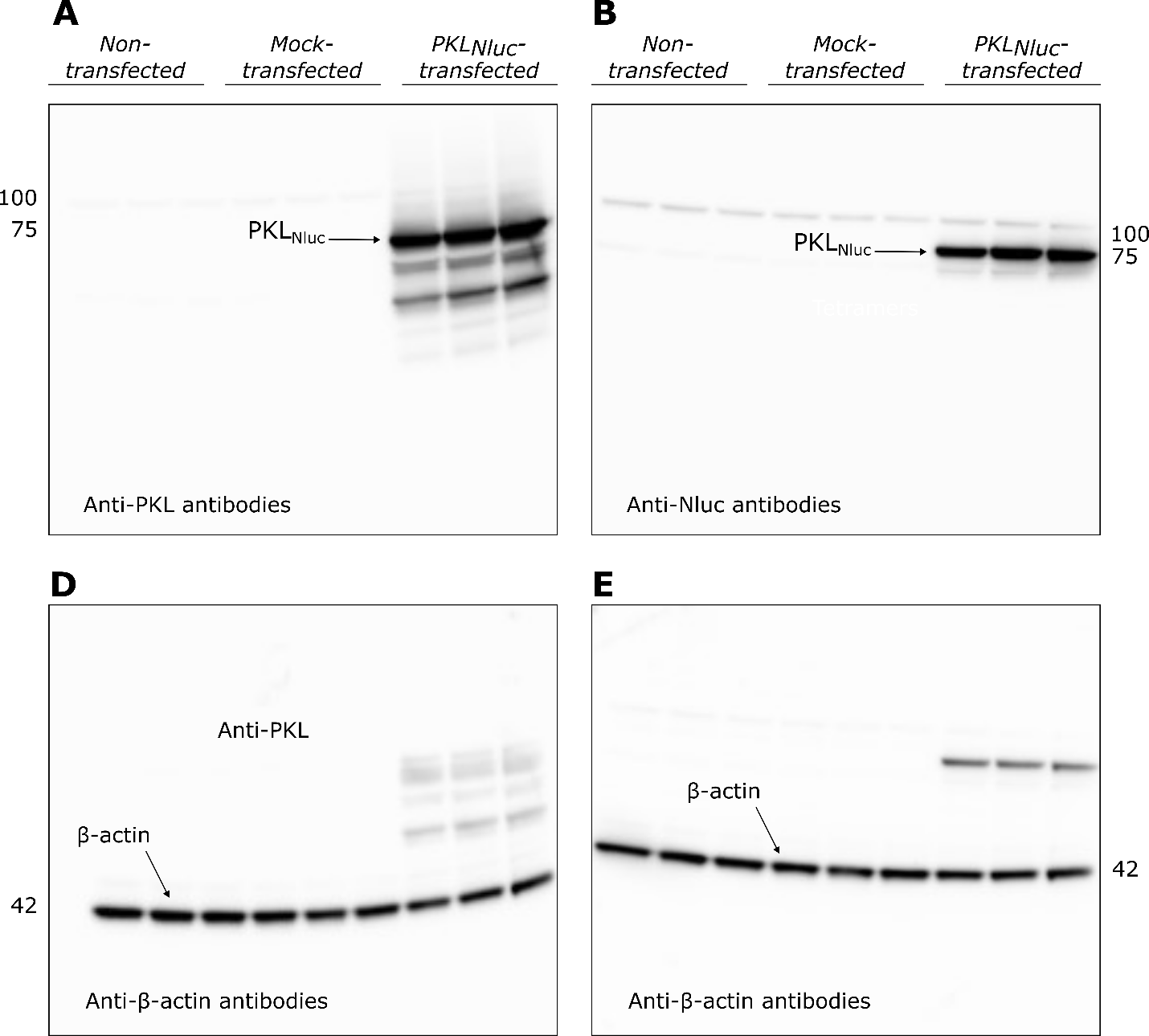
**

**Figure S13.** Western blots showing overexpression of PKL_Nluc_ in Hek293 cells. The cells were transfected with complexes of PKL_Nluc_ plasmid and carrier DNA using Fugene HD transfection reagent. Another two sets of cells were either non-transfected or mock-transfected with carrier DNA. The protein concentrations of the cell lysates (triplicates for each transfection) were equalised, and lysates were separated on SDS-PAGE gels under denatured and reduced conditions, transferred to PVDF membranes, blocked, and incubated with the respective primary and secondary antibodies. Panels show chemiluminescence imaging of the following sequences of antibodies: (A) Incubation with primary rabbit polyclonal anti-PKL antibodies followed by secondary donkey anti-rabbit-HRP antibodies. (B) Incubation with mouse monoclonal anti-Nluc antibodies followed by secondary sheep-anti-mouse-HRP antibodies. (C-D) Incubation of membranes from A and B, respectively, after stripping, with mouse monoclonal anti-β-actin antibodies, followed by sheep anti-mouse-HRP antibodies.


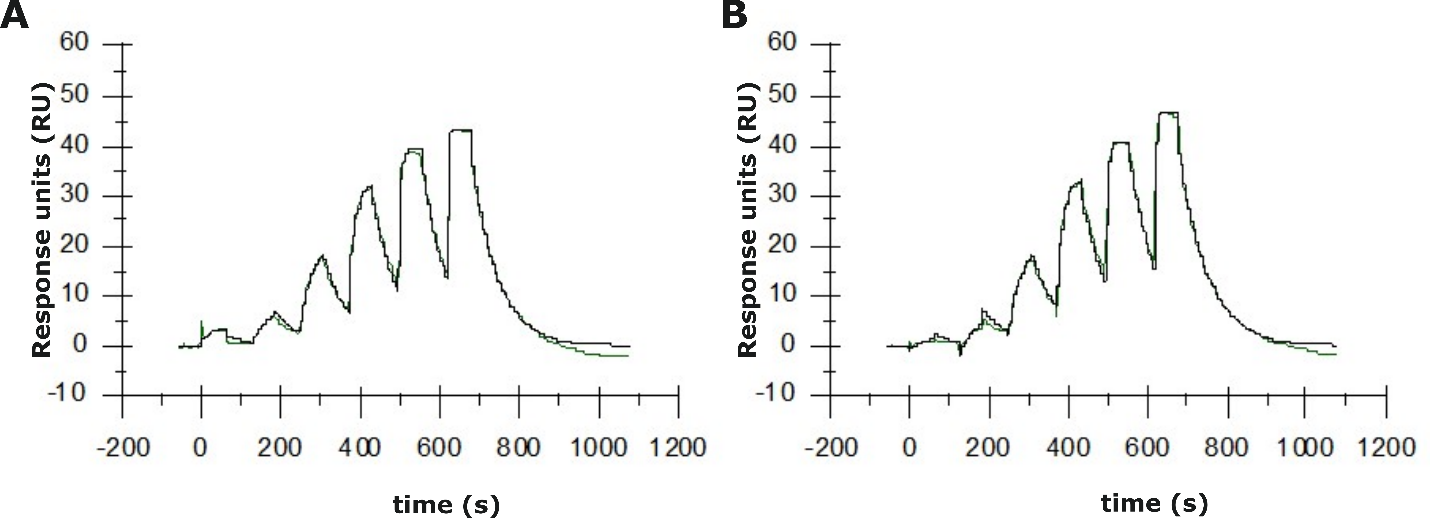


**Figure S14.** Representative sensorgrams using single cycle kinetics of **IV** (green) with corresponding fit to a 1:1 Langmuir interaction model. The two panels show data from two independent replicates of the titration. (A) *K*_D_ = 38.8 nM (B) *K*_D_ = 36.9 nM.

**Table S2.** Crystallographic Data Collection and refinement statistics.

| **PDB code** | **9RFQ** | **9RFT** | **9RDF** |
| --- | --- | --- | --- |
| Ligand | **I** | **II** | **IV** |
|  |  |  |  |
| **Data collection:** |  |  |  |
| Beamline | Diamond Light Source i03 | Diamond Light Source i03 | Diamond Light Source i04 |
| Wavelength (Å) | 0,9763 | 0,9763 | 0,9537 |
| Resolution range (Å) | 103.63-2.38 (2.85-2.38) | 188.53-1.89 (2.20-1.89) | 78.65 - 1.62 (1.650 - 1.620) |
| Space group | C 1 2 1 | C 1 2 1 | C 1 2 1 |
| Unit cell (a, b, c)(Å) | 207.36, 112.74, 187.80 | 207.96, 112.97, 188.59 | 200.18, 97.58, 136.40 |
| Unit cell (α, β, γ)(°) | 90.0, 91.8, 90.0 | 90.0, 91.4, 90.0 | 90.00, 118.53, 90.00 |
| Total reflections | 596569 (26961) | 1316805 (64506) | 2055338 (98680) |
| Unique reflections | 83049 (4152) | 183791 (9190) | 292837 (14270) |
| Multiplicity | 7.2 (6.5) | 7.2 (7.0) | 7.0 (6.9) |
| Completeness (%) | 92.5 (62.0) | 91.0 (61.2) | 99.9 (97.8) |
| Mean I/sigma(I) | 6.1 (1.7) | 8.0 (1.4) | 10.9 (0.3) |
| R-merge | 0.24 (1.19) | 0.18 (1.43) | 0.090 (3.86) |
| R-meas | 0.10 (0.50) | 0.07 (0.57) | 0.037 (1.57) |
| CC-half | 0.993 (0.705) | 0.997 (0.203) | 0.979 (0.30) |
|  |  |  |  |
| **Refinement:** |  |  |  |
| Resolution range (Å) | 187.71-2.38 (2.70-2.38) | 188.50-2.27 (2.49-2.27) | 78.65 - 1.62 (1.63 - 1.62) |
| R-factor/Rfree | 0.21/0.24 (0.27/0.33) | 0.21/0.24 (0.28/0.30) | 0.24/0.28 (0.56/0.58) |
| Number of total atoms | 26520 | 27747 | 16915 |
| Number of ligand atoms | 224 | 257 | 119 |
| Number of waters | 628 | 1833 | 1140 |
| RMS bond lengths (Å) | 0,007 | 0,008 | 0,01 |
| RMS bond angles (°) | 0,88 | 0,89 | 0,96 |
| RMS dihedral angles (°) | 2,53 | 2,74 | 3,29 |
| Average B-factors (Å^2) |  |  |  |
| All atoms | 50,8 | 58,7 | 47,5 |
| Ligands | 49,6 | 60,3 | 49,0 |
| Waters | 30,5 | 51,6 | 38,1 |

1 ATGGTCTTCACACTCGAAGATTTCGTTGGGGACTGGCGACAGACAGCCGGCTACAACCTG 60

1 M V F T L E D F V G D W R Q T A G Y N L 20

61 GACCAAGTCCTTGAACAGGGAGGTGTGTCCAGTTTGTTTCAGAATCTCGGGGTGTCCGTA 120

21 D Q V L E Q G G V S S L F Q N L G V S V 40

121 ACTCCGATCCAAAGGATTGTCCTGAGCGGTGAAAATGGGCTGAAGATCGACATCCATGTC 180

41 T P I Q R I V L S G E N G L K I D I H V 60

181 ATCATCCCGTATGAAGGTCTGAGCGGCGACCAAATGGGCCAGATCGAAAAAATTTTTAAG 240

61 I I P Y E G L S G D Q M G Q I E K I F K 80

241 GTGGTGTACCCTGTGGATGATCATCACTTTAAGGTGATCCTGCACTATGGCACACTGGTA 300

81 V V Y P V D D H H F K V I L H Y G T L V 100

301 ATCGACGGGGTTACGCCGAACATGATCGACTATTTCGGACGGCCGTATGAAGGCATCGCC 360

101 I D G V T P N M I D Y F G R P Y E G I A 120

361 GTGTTCGACGGCAAAAAGATCACTGTAACAGGGACCCTGTGGAACGGCAACAAAATTATC 420

121 V F D G K K I T V T G T L W N G N K I I 140

421 GACGAGCGCCTGATCAACCCCGACGGCTCCCTGCTGTTCCGAGTAACCATCAACGGAGTG 480

141 D E R L I N P D G S L L F R V T I N G V 160

481 ACCGGCTGGCGGCTGTGCGAACGCATTCTGGCGATGGAAGGTCCAGCAGGTTATCTGCGT 540

161 T G W R L C E R I L A M E G P A G Y L R 180

541 CGCGCATCTGTGGCCCAGCTGACCCAAGAGCTGGGCACCGCATTTTTCCAGCAGCAGCAG 600

181 R A S V A Q L T Q E L G T A F F Q Q Q Q 200

601 TTGCCTGCCGCCATGGCCGATACCTTTCTGGAACACCTGTGCCTGCTGGACATCGACTCT 660

201 L P A A M A D T F L E H L C L L D I D S 220

661 GAGCCTGTGGCCGCCAGATCCACCTCCATCATTGCTACCATCGGACCCGCCTCCAGATCC 720

221 E P V A A R S T S I I A T I G P A S R S 240

721 GTGGAACGGCTGAAAGAGATGATCAAGGCCGGCATGAATATCGCCCGGCTGAACTTCTCT 780

241 V E R L K E M I K A G M N I A R L N F S 260

781 CACGGCTCTCACGAGTACCACGCCGAGTCTATCGCTAATGTGCGCGAGGCCGTGGAATCT 840

261 H G S H E Y H A E S I A N V R E A V E S 280

841 TTTGCCGGCTCTCCACTGTCCTACAGACCTGTGGCTATCGCCCTGGATACCAAGGGACCT 900

281 F A G S P L S Y R P V A I A L D T K G P 300

901 GAGATCAGAACCGGCATCCTGCAAGGCGGACCTGAGTCTGAGGTGGAACTGGTTAAGGGC 960

301 E I R T G I L Q G G P E S E V E L V K G 320

961 TCCCAGGTGCTGGTCACCGTGGATCCTGCTTTCAGAACCAGAGGCAACGCCAACACCGTG 1020

321 S Q V L V T V D P A F R T R G N A N T V 340

1021 TGGGTCGACTACCCTAACATCGTCAGAGTGGTGCCTGTCGGCGGCAGAATCTACATCGAC 1080

341 W V D Y P N I V R V V P V G G R I Y I D 360

**Figure S15.** DNA and protein sequences of the PKL_Nluc_ fusion construct. Nluc sequence is coloured green and the PKL sequence in purple.

1081 GATGGCCTGATCTCCCTGGTGGTGCAGAAGATTGGACCTGAGGGACTCGTGACCCAGGTG 1140

361 D G L I S L V V Q K I G P E G L V T Q V 380

1141 GAAAATGGCGGAGTGCTGGGAAGCAGAAAGGGCGTTAACTTGCCTGGCGCTCAGGTTGAC 1200

381 E N G G V L G S R K G V N L P G A Q V D 400

1201 CTGCCTGGACTGTCTGAACAGGACGTGCGGGATCTGAGATTTGGCGTGGAACACGGTGTC 1260

401 L P G L S E Q D V R D L R F G V E H G V 420

1261 GATATCGTGTTCGCCTCTTTCGTGCGGAAGGCCTCTGATGTGGCTGCTGTTAGAGCTGCT 1320

421 D I V F A S F V R K A S D V A A V R A A 440

1321 CTGGGCCCTGAAGGCCACGGCATCAAGATCATCTCCAAGATCGAGAACCACGAGGGCGTG 1380

441 L G P E G H G I K I I S K I E N H E G V 460

1381 AAGAGATTCGACGAGATCCTGGAAGTGTCCGACGGCATCATGGTGGCTAGAGGCGATCTG 1440

461 K R F D E I L E V S D G I M V A R G D L 480

1441 GGAATCGAGATCCCCGCCGAAAAGGTGTTCCTGGCTCAGAAAATGATGATCGGCAGATGC 1500

481 G I E I P A E K V F L A Q K M M I G R C 500

1501 AACCTGGCCGGCAAGCCTGTTGTGTGTGCTACCCAGATGCTGGAATCCATGATCACCAAG 1560

501 N L A G K P V V C A T Q M L E S M I T K 520

1561 CCTCGGCCTACCAGAGCCGAGACATCCGATGTGGCTAATGCTGTGCTGGACGGCGCCGAC 1620

521 P R P T R A E T S D V A N A V L D G A D 540

1621 TGTATCATGTTGTCTGGCGAGACAGCCAAGGGCAACTTCCCTGTGGAAGCCGTGAAGATG 1680

541 C I M L S G E T A K G N F P V E A V K M 560

1681 CAGCACGCTATCGCCAGAGAAGCCGAGGCTGCTGTGTACCACAGACAGCTGTTCGAGGAA 1740

561 Q H A I A R E A E A A V Y H R Q L F E E 580

1741 CTGCGGAGAGCTGCCCCTCTGTCCAGAGATCCTACCGAAGTGACAGCCATCGGAGCCGTG 1800

581 L R R A A P L S R D P T E V T A I G A V 600

1801 GAAGCTGCCTTTAAGTGTTGCGCCGCTGCCATCATCGTGCTGACCACAACTGGAAGATCT 1860

601 E A A F K C C A A A I I V L T T T G R S 620

1861 GCCCAGCTGCTGTCCCGGTATAGACCTAGAGCTGCCGTGATCGCCGTGACCAGATCTGCT 1920

621 A Q L L S R Y R P R A A V I A V T R S A 640

1921 CAGGCTGCCAGACAGGTTCACCTGTGTAGGGGAGTGTTCCCACTGCTGTACAGAGAGCCT 1980

641 Q A A R Q V H L C R G V F P L L Y R E P 660

1981 CCTGAGGCCATCTGGGCCGACGATGTGGATAGAAGAGTGCAGTTCGGCATCGAGTCCGGC 2040

661 P E A I W A D D V D R R V Q F G I E S G 680

2041 AAGCTGAGAGGATTCCTGAGAGTGGGCGATCTCGTGATCGTGGTTACAGGATGGCGGCCT 2100

681 K L R G F L R V G D L V I V V T G W R P 700

2101 GGCTCCGGCTACACCAATATCATGAGAGTGCTGTCCATCTCCTAA 2145

701 G S G Y T N I M R V L S I S * 714

**Figure S15 (continuation).** DNA and protein sequences of the PKL_Nluc_ fusion construct. Nluc sequence is coloured green and the PKL sequence in purple.

# **Equations**

*Equation 1 – Hill equation^[2]^*

*(Eq. 1)* $E=E_{0}+\frac{E_{max}\cdot{[L]}^{n}}{{({EC}_{50})}^{n}+L^{n}}$

Where *E* is the observed efficacy at any given effector concentration, *E_0_* is the observed efficacy (response) in the absence of effector, *E_max_* is the maximal observed efficacy (response), *[L]* is the effector concentration, *EC_50_* is the half-maximum response effector concentration, and *n* is the Hill coefficient.

*Equation 2 – Exponential association function^[5]^*

*(Eq. 2)* $R=R_{inf}\cdot(1-e^{-k_{obs}\cdot t})$

Where *R* is the fluorescence response (approximation of target-ligand complex formation), *t* is the time, and *R_inf_* is the fluorescence response at the plateau, i.e. at infinite time. *k_obs_* is the observed rate constant at any given protein- or ligand-concentration.

*Equation 3 – Rate constants relation^[5]^*

*(Eq. 3)* $K_{D}=\frac{k_{off}}{k_{on}}$

Where *K_D_* is the equilibrium binding constant, *k_on_* is the association rate constant, and *k_off_* is the dissociation rate constant.

*Equation 4 – The Cheng-Prusoff equation^[6]^*

*(Eq. 4)* $K_{D}=\frac{{EC}_{50}}{1+\frac{[T]}{{EC}_{50}^{T}}}$

Where *K_D_* is the estimated equilibrium binding constant, *EC_50_* is the observed half-maximum response competitor concentration, *[T]* is the tracer concentration, and EC_50_^T^ is the observed half-maximum response concentration of the tracer.

# **Experimental procedures**

## *X-ray crystallography*

Expression and purification of recombinant PKL (with or without a deletion of B-domain), protein crystallization, crystal soaking and structure determination was performed as described previously^[7]^ Structure of I and II in complex with PKL were obtained by soaking into crystals of PKL lacking the B-domain, as described before.^[7]^ Crystals of complex of **IV** with PKL were obtained by co-crystallisation of full-length PKL (including the B-domain) at concentration was 9.5 mg/ml and total concentration of IV of 200 µM. The complex crystallised from Morpheus Fusion screen condition with 1.5 %w/v VitMix; 0.12 M Monosacc ; 0.1 M MB1 6.5 pH (Buffer); 37.5 %v/v PptMix4. All structures and corresponding structure factors have been deposited to Protein Data Bank under accession codes 9RFQ (**I**), 9RFT (**II**) and 9RDF (**IV**) (Table S2).

## *Buffer stability measurement of compounds*

Stock solutions of compounds were prepared in DMSO at a concentration of 10 mM. These solutions were subsequently diluted in either serum-free Tris-HCl buffer (50 mM Tris-HCl, pH 7.4, 10 mM MgCl₂, 100 mM KCl, 0.05% Tween-20) or serum-supplemented (1% FBS) Opti-MEM™ medium to achieve a final compound concentration of 100 µM, with the DMSO content adjusted to 1% (v/v). Aliquots of 1 mL were incubated at 37 °C and continuously monitored using high-performance liquid chromatography (Waters 2690 Separation Module; 996 Photodiode Array Detector). Chromatographic separation was carried out on a C18 reverse-phase column (), employing a gradient elution with mobile phases consisting of water containing 0.1% trifluoroacetic acid (TFA) and acetonitrile, at a flow rate of 2.0 mL/min. 30 µL injections were done after 0, 12, 24, 36, 48, 60, and 72 h of incubation time, and the samples were monitored at 254 nm. The retention time and peak area of the parent compound were recorded at each time point. Peak areas were normalized to the peak integral at t=0 h in each sample to determine the percentage of compound remaining over time. Compound stability was evaluated based on the percentage remaining at each time point. Data analysis was performed using Origin(Pro) 2023.^[8]^

## *PKL activity assays*

PKL reactions were performed in triplicate in 384-well white microplates (Corning™ 384-Well White PS 3824) at room temperature. Each 5 µL reaction was conducted in Tris-HCl buffer (50 mM Tris-HCl, pH 7.4, 10 mM MgCl₂, 100 mM KCl, 0.05% Tween) and incubated for 10 minutes. The reaction mixture contained 0.2 mM ADP, 0.1 mM PEP, 5 nM PKL, 1% DMSO, and test compounds at concentrations ranging from 0 to 1 mM across 12 titration points. Prior to substrate addition, PKL and test compounds were pre-incubated for 15 minutes.

After the reaction, 5 µL of Kinase-Glo Max reagent was added to each well. Luminescence was measured using a SpectraMax iD5 microplate reader with the following settings: all wavelengths read, 100 ms integration time, 1.0 mm read height, and a read temperature of 25 °C. For each data set, luminescence from the DMSO control was set as 100% activity, and luminescence from the protein blank (no PKL) as 0% activity. Percentage activity (E%) was calculated using the equation:

$$\boldsymbol{E\%}=\frac{\mathbf{L}_{\mathbf{c}}-\mathbf{L}_{\mathbf{o}}}{\mathbf{L}_{\mathbf{DMSO}}-\mathbf{L}_{\mathbf{o}}}$$

Where:

- ***L_C_*** = luminescence with test compound
- ***L_DMSO_*** = luminescence with DMSO (negative control)
- ***L_0_*** = luminescence without PKL

Activity data was analyzed using Origin(Pro) 2023.^[8]^ Titration data was fitted to the Hill equation in Origin to determine EC₅₀ values.

## *HepG2 cellular PKL activity assay*

HepG2 PKM2 KO cells (Synthego) were plated in 6 well plates at cell densities of 2 x 10^5^ cells/well and incubated for 24 h at 37 °C and 5% CO_2_. Compounds were added to the cells in different concentrations (0.1, 1, 10 µM) and the cells were further incubated for 48 h. The control cells received DMSO as vehicle.

Cells were washed with PBS, lysed on ice with 200 µL/well PK assay buffer. The protein concentration of the cell lysates was adjusted to 200 µg/ml. Pyruvate kinase (PK) activity of the cell lysates (10 µg total protein) was analyzed by using the PK assay kit from abcam (ab83432) according to manufacturer´s instructions. The PKL activity in the cell lysates treated with the compounds was expressed as a percentage of the PK activity in the vehicle.

## *Characterization of fluorescent properties*

Excitation and emission spectra of the tracers were recorded in air-equilibrated Milli-Q water or MeCN using a Cary Eclipse spectrofluorometer (Varian Inc., Mulgrave, Victoria, Australia) at room temperature in quartz cuvettes (light path 10x4 mm). Slit widths for both excitation and emission spectra were set to 5 nm. Net spectra were obtained by subtracting the corresponding vehicle reference spectra, and corrected emission spectra were calculated by applying the appropriate lamp correction factors. Quantum yields were determined following established protocols,^[9]^ with slight modifications, using the same spectrofluorometer and Coumarin 153 (Merck) as the fluorescence standard. Absorption spectra were measured via UV/Vis spectroscopy at an optical density of ~0.2 A using an CaryBio 50 UV/Vis spectrophotometer (Varian Inc., Mulgrave, Victoria, Australia).

## *Surface plasmon resonance*

Compound affinities to recombinant PKL_wt_ or PKM2 was determined in a direct binding assay using a S200/T200 surface plasmon resonance (SPR) biosensor (Cytiva) at 20°C. Briefly, PKL or PKM2 was immobilized on a CM5 sensor chip (Cytiva) in the presence of 10 µM mitapivat in 10 mM MES, pH=6.3. The surface was washed with 10 mM NaOH, 1M NaCl followed by immobilization of protein before subsequent deactivation by 1 M ethanolamine. Immobilization levels were typically 12000-16000 RU. The reference spot was treated as described, omitting the injection of PKL. Compound concentration series was injected over the immobilized protein in increasing concentrations using multi cycle (MCK) or single cycle kinetics (SCK) in running buffer (10 mM HEPES, 150 mM NaCl, 0.05% Tween20, 0.3% DMSO, pH 7.4). A 1:1 Langmuir interaction model is fitted to the experimental traces, enabling determination of *k*_on_, *k*_off_ and *K*_d_. Representative sensorgrams of titrations with compound **IV** are displayed in Figure S14.

## *Fluorescence indication titrations of tracer* ***IV***

Titrations of tracer **IV** were performed in the presence and absence of 10 µM FBP. Tracer **IV** was serially diluted in Tris-HCl buffer (pH 7.4; 50 mM Tris, 10 mM MgCl₂, 100 mM KCl, 0.05% Tween) to generate 2x tracer working solutions (0–10 µM, 3:1 dilution, 12 concentrations). PKL was prepared as a 2x working solution at a constant concentration used throughout the titration. Equal volumes (10 µL) of 2x tracer and 2x PKL solutions were mixed (1:1) in 384-well microplates (Corning Black PS, 3820). For each experiment, three 12-point triplicate series were prepared containing either: (1) tracer **IV** and buffer (no PKL), (2) tracer **IV** and PKL (0.1 µM), (3) tracer **IV** and PKL (0.1 µM) and mitapivat (10 µM, competitor control). Following an incubation period of 1 h at ambient temperature, the fluorescence intensities were measured using a SpectraMax iD5 microplate reader (Molecular Devices) with excitation at 450 nm and emission at 550 nm (integration time: 400 ms; read height: 1.0 mm). Titration data were analyzed in Origin(Pro) 2023^[8]^ and fitted to the Hill equation to determine EC₅₀ values.

## **Confocal microscopy**

*Cellular uptake of* ***IV***

To attach HEK293 cells to the bottom of 24-well imaging plates (Ibidi 82427), a Cell-Tak adhesive mixture (Corning, 354240) was used. It was prepared with a mixture of sodium bicarbonate (75 g/L), sodium hydroxide (40 g/L) and Cell-Tak (2.03 g/L) at a volume ratio of 291:5:4. For each well, 500 μL of Cell-Tak mix was added and incubated at room temperature for 30 min. The Cell-Tak mix was then removed, and the dishes were washed three times with H_2_O before cell plating. HEK293 cells were adjusted to the cell density of 2 x 10^5^ cell/ml in FluoroBrite™ DMEM medium (Invitrogen, 896701) supplemented with 10% FBS (Hyclone), 4 mM L-glutamine and 2.5 mM sodium pyruvate. 500 µL of this suspension (1 x 10^5^ cells) was added to each well, and the cells were allowed to attach to the surface for 30 min at ambient temperature and then incubated at 37 °C in 5% CO_2_ incubator for 24 h. Cells were then treated with **IV** (1 µM) and images of cellular uptake were captured every 3 seconds immediately after addition without washing. For analysing intracellular fluorescence and distribution of **IV** within HEK293 cells, Z-stack images (22 images) were acquired after 5, 15, 30 and 60 min of incubation across a physical depth of ~22 μm. Imaging data were collected on a Carl Zeiss LSM 980 confocal microscope equipped with a Plan-Apochromat 20X / 0.8 objective. Cells were visualised upon excitation at 488 nm by using a 32-channel GaAsP PMT Detector in the spectral range (500-630 nm). Bright-field images were captured with a halogen lamp. Conditions were maintained at 37°C and 5% CO_2_. Image processing used ZEN software, and analysis was performed with ImageJ.^[10]^

## *HEK293 cell viability assay (CellTiter-Glo 2.0)*

The effect of tracer **IV** on cell viability was assessed using CellTiter-Glo 2.0 assay (Promega). This is a method that determines the number of viable cells by quantifying the amount of ATP in the metabolically active cells. The CellTiter-Glo reagent contains Ultra-GloT^M^ luciferase, its substrate luciferin and a reagent which lyses the cells leading to the release of the cellular ATP. Mono-oxygenation of luciferin catalyzed by luciferase in the presence of ATP will generate light which could be measured by luminometer. The amount of ATP is directly proportional to the number of cells.

*HEK293 cells in FluoroBrite™ medium – Confocal microscopy conditions*

HEK293 cells were plated in FluoroBrite™ DMEM complete medium (medium supplemented with 10% FBS, 4 mM L-glutamine and 2 mM Sodium pyruvate) at density 2 x 10^4^ cells/well in opaque 96 well plates suitable for luminescence readings (Costar 3917) and incubated for 24 h at 37 °C and 5% CO_2_. The cells were treated for 2 h with different concentrations of the tracer (0.01, 0.1, 1 and 10 µM); the vehicle-treated cells received the same volume of DMSO. After the incubation period, the plate was allowed to reach ambient temperature for 30 min. Thereafter, 100 µL/well of CellTitter-Glo 2.0 reagent was added and the plate was shaken for 2 min to induce the cell lysis. After incubation of plate for 10 min at 22 °C, the total luminescence was recorded with SpectraMax iD5 microplate reader (Molecular Devices) using the following settings: 1000 ms integration time and 3 mm height reading from the top.

*Cell viability in serum-supplemented Opti-MEM™ medium – NanoBRET conditions*

HEK293 cells (4x10^6^ cells in Optimem+1% FBS) were transfected with 1 µg PKL_Nluc_ or Nluc plasmids, 9 µg of carrier DNA and 30 µL Fugene HD (same protocol as for NanoBRET assay recommended by Promega). Transfected cells were plated at density 2x10^4^ cells/well in opaque 96 well plates suitable for luminescence readings (Costar 3917) and incubated for 24 h at 37 °C and 5% CO_2_. The cells were incubated for 2 h with different concentrations of the tracer (0-160 nM). After the incubation period, the plate was allowed to reach room temperature for 30 min. Thereafter, 100 µL/well of CellTitter-Glo 2.0 reagent was added and the plate was shaken for 2 min to induce the cell lysis. After incubation of plate for 10 min at 22 °C, the total luminescence was recorded with Spectramax iD5 apparatus using the following settings :1000 ms integration time and 3 mm height reading from the top.

## *AlphaFold structure model of PKL_Nluc_ fusion protein*

The structure of the PKL_Nluc_ fusion protein was predicted using Alpha Fold 3 server.^[11]^ The positional alignment score indicates strong confidence in the structure of tetrameric PKL, lower confidence for NanoLuc, and very low confidence in the N-terminal linker between the proteins (Figure S4). Consequently, the final placement of the NanoLuc domain outside the tetrameric PKL assembly should be interpreted with caution.

Input sequence (Protein, 4 copies, seed = 1):

*MVFTLEDFVGDWRQTAGYNLDQVLEQGGVSSLFQNLGVSVTPIQRIVLSGENGLKIDIHVIIPYEGLSGDQMGQIEKIFKVVYPVDDHHFKVILHYGTLVIDGVTPNMIDYFGRPYEGIAVFDGKKITVTGTLWNGNKIIDERLINPDGSLLFRVTINGVTGWRLCERILAMEGPAGYLRRASVAQLTQELGTAFFQQQQLPAAMADTFLEHLCLLDIDSEPVAARSTSIIATIGPASRSVERLKEMIKAGMNIARLNFSHGSHEYHAESIANVREAVESFAGSPLSYRPVAIALDTKGPEIRTGILQGGPESEVELVKGSQVLVTVDPAFRTRGNANTVWVDYPNIVRVVPVGGRIYIDDGLISLVVQKIGPEGLVTQVENGGVLGSRKGVNLPGAQVDLPGLSEQDVRDLRFGVEHGVDIVFASFVRKASDVAAVRAALGPEGHGIKIISKIENHEGVKRFDEILEVSDGIMVARGDLGIEIPAEKVFLAQKMMIGRCNLAGKPVVCATQMLESMITKPRPTRAETSDVANAVLDGADCIMLSGETAKGNFPVEAVKMQHAIAREAEAAVYHRQLFEELRRAAPLSRDPTEVTAIGAVEAAFKCCAAAIIVLTTTGRSAQLLSRYRPRAAVIAVTRSAQAARQVHLCRGVFPLLYREPPEAIWADDVDRRVQFGIESGKLRGFLRVGDLVIVVTGWRPGSGYTNIMRVLSIS*

## *Molecular cloning of expression constructs*

Constructs for production of PKL in E. coli have been described before.^[7]^ Expression construct for PKM2 was a kind gift from Dr Teodors Pantelejevs, Latvian Institute for Organic Synthesis.^[12]^ The Nluc construct and Nluc luciferase fusion with PKL was cloned into pcDNA3.0 plasmid by fusion synthetic coding sequence of the luciferase directly to the 5’ of human PKL coding sequence. The sequence of the fusion protein is shown in Figure S15. The resulting plasmid pcDNA3.0-Nluc-PKL has been reposited at Addgene under accession code **240797**, [link](https://www.addgene.org/240797/).

For *E. coli* expression the fragment encoding for the Nano-Luciferase fusion protein was cloned into pExp-NHis expression vector (Addgene plasmid #112558) with additional GlySer linker between the TEV cleavage site and the start of the nano-luciferase coding region.

## *Native PAGE electrophoresis*

Recombinant PKL_NLuc_ (4 x 950 ng) was incubated for 1 hour at ambient temperature with different concentrations of compound **IV** in a total volume of 60 µL Tris-HCl buffer pH 7.4 (50 mM Tris, 100 mM KCl, 10 mM MgCl2, 0.05% Tween 20). PKM2 and PKL recombinants (2 x 950 ng) were also incubated for 1 hour at ambient temperature with different concentrations of the compound **IV** (0, 2.5 and 10 µM). Samples were mixed with native PAGE sample buffer and native PAGE G-250 sample additive, and the proteins (690 ng/lane) were separated by Native PAGE electrophoresis on Native PAGE 3-12% Novex Bis-Tris gels (Invitrogen). For experiments in the presence of FBP, the PKM2, PKL and PKL _Nluc_ recombinant proteins (2x950 ng) were incubated for 1 hour at ambient temperature in the presence of 10 µM FBP and different concentrations of the compound **IV** (0, 2.5 and 10 µM).

A ChemiDoc imager (Bio-Rad, Alexa 488 filter) was used for fluorescence detection of compound **IV** bound to the proteins. Thereafter the gel was incubated with 0.1x Nano-Glo® substrate (Promega, N1571) to detect PKL_Nluc_. The images of the in-gel fluorescence and luminescence detection were superimposed. Thereafter the same gels were stained for detection of the proteins with Colloidal blue staining kit (Invitrogen) according to manufacturer´s instructions. The second set of gels were used for transfer of the protein on PVDF membranes followed by immunoblotting with anti-PKL antibodies (R&D systems, AF8519, diluted 1:1000 in 5% milk TBS-Tween 20) and secondary antibodies donkey–anti-rabbit HRP (Cytiva, NA934V, diluted 1:5000 in 5% milk TBS-Tween 20). The chemiluminescent signal was developed with a ChemiDoc imager (BioRad) after incubation with HRP substrate (Supersignal west femto maximum sensitivity substrate, Thermo Fisher Scientific, 34095). The same membrane was stripped with restore plus western blot stripping buffer (Thermo Fisher Scientific, 46430) and incubated overnight at 4°C with rabbit monoclonal anti-PKM2 antibodies (Cell Signaling, 4053, diluted 1:1000 in 5% BSA TBS-Tween 20) followed by secondary antibodies donkey–anti-rabbit HRP and chemiluminescence detection as described above.

## *NanoBRET Experiments with recombinant PKL_Nluc_*

All NanoBRET measurements were performed as triplicates in 384-well white microplates (Corning™ 384-Well White PS 3824) at room temperature. Mixtures (5 µL) of recombinant PKL_Nluc_ protein (10 nM), tracer **IV** (0.2 µM), mitapivat (20 µM, non-specific control) or DMSO in Tris-HCl buffer (50 mM Tris-HCl, pH 7.4, 10 mM MgCl₂, 100 mM KCl, 0.05% Tween, 2% DMSO total) were incubated for 30 min prior to addition of 5 µL 0.1x Nano-Glo® substrate (Promega, N1571) and the luminescence was recorded after 5 min of substrate addition using a Spectramax iD5 plate reader (molecular devices) with 1000 ms integration time. The donor/acceptor wavelength pairs were recorded at 450/470 nm, 550/510 nm, 450/530 nm, 450/550 nm, 450/610 nm, 450/630 nm and 400/470 nm respectively. For each sample well, BRET ratios were calculated using the following formula:

$$BRET ratio \left( \mathrm{mBU} \right)= \frac{\mathrm{Acceptor}_{S}}{\mathrm{Donor}_{S}}-\frac{\mathrm{Acceptor}_{\mathrm{NT}}}{\mathrm{Donor}_{\mathrm{NT}}}$$

Where Acceptor_S_ is the luminescence intensity (FI) of the sample well at 550 nm, Donor_S_ is the FI of the sample well at 450 nm, and Acceptor_NT_ and Donor_NT_ are the FI of the no-tracer control at 550 nm and 450 nm respectively.

## *Protein production*

Production of PKL proteins for enzyme assays, for nanoBRET optimization and for crystallography were done as described below. PKM2 was produced according to published protocols.^[12]^

*E. coli* T7 Express cells (NEB) transformed with the appropriate pExp-NHis plasmid encoding for PKL or nano-Luc-PKL fusion were grown to an A_600_ of 1.0 at 37 °C with shaking at 200 rpm in 2x1 L of 2xYT medium. Recombinant protein expression was induced with 0.4 mM IPTG and proceeded at 18 °C with shaking at 170 rpm for an additional 16-18 h.

The cells were harvested by centrifugation and resuspended in 50 ml of 20 mM Tris-HCl pH 8.5, 500 mM NaCl, 20 mM Na_2_HPO4, 20 mM imidazole, 0.5 mM TCEP, 1 mM AEBSF. Next, 200 Kunitz units of DNase I was added to the sample, and the cells were lysed by sonication on ice. The lysate was cleared by centrifugation at 40,000 × g for 30 min, filtered through 0.45 µm filter and loaded onto a gravity flow column packed with 5 ml of HIS-Select HF Nickel Affinity Gel (Sigma) pre-equilibrated with 20 mM Tris-HCl pH 8.5, 25 mM NaCl, 0.5 mM TCEP. The column was washed with 10 column volumes of the same buffer, and the proteins were then eluted with 20 mM Tris-HCl pH 8.5, 500 mM NaCl, 20 mM Na_2_HPO_4_, 250 mM imidazole, 0.5 mM TCEP.

N-terminal 8xHis tag was removed using TEV protease (purified in house) at a molar ratio of 1:100 (TEV: PKL) at 4 °C overnight. After cleavage the protein sample was exchanged to 20 mM Tris-HCl pH 8.5, 25 mM NaCl, 20 mM imidazole, 0.5 mM TCEP using a buffer exchange column and then passed through a HIS-Select HF Nickel column again to remove uncleaved PKL, cleaved His-tag and His-tagged TEV protease. The protein solution was loaded onto a HiTrap Q HP 5 ml anion exchange column (GE Lifesciences) pre-equilibrated with 20 mM Tris-HCl pH 8.5, 25 mM NaCl, 0.5 mM TCEP > protein was eluted with a gradient to 20 mM Tris-HCl pH 8.5, 1000 mM NaCl, 0.5 mM TCEP. Pooled peak fractions were buffer exchanged to 50 mM Tris-HCl pH 7.4, 10 mM MgCl_2_, 100 mM KCl, 0.05% Tween®-20, concentrated to ca. 3.4 mg/ml, flash frozen in liquid nitrogen in 30uL aliquots, and stored at −70 °C.

## *Overexpression of PKL_Nluc_ in HEK293 cells*

To check the overexpression of PKL_Nluc_ in HEK293 cells, the cells were plated in 6 well plates at concentration of 3 x 10^5^ cells/well in FluoreBrite medium (Invitrogen) supplemented with 10% FBS (Hyclone), 4 mM L-glutamine and 5 mM sodium pyruvate. Twenty-four hours after plating, the cells were transfected with complexes of PKL_Nluc_ plasmid (0.15 µg/well) in the presence of carrier DNA (Promega, 1.35 µg/well) in Opti-MEM™ medium (145.5 µL/well) using Fugene HD (Promega) transfection reagent (4.5 µL/well). One set of cells was left non-transfected and another set of cells was mock transfected (transfected with only carrier DNA). Twenty-four hours after transfection, the cells were washed 3 x PBS. Cells were lysed for 5 min on ice with RIPA buffer (Cell Signaling, 9806) containing phosphatase and protease inhibitors (Halt Protease and Phosphatase Inhibitor Cocktail,Thermo Fisher Scientific 87785) (150 µL/well) and thereafter under rotation for 20 min at 4°C. The cell lysates were centrifuged at 28,000 x g for 10 min at 4°C and the supernatants were saved in new tubes. The protein concentration of the clear cell lysates was determined using BCA assay kit (Pierce BCA Protein Assay Kit, Thermo Fisher Scientific, 23225) according to the manufacturer´s instructions. The protein concentration of the cell lysates was then adjusted to the same concentration with RIPA buffer. The samples were mixed with LDS buffer and reducing agent (Invitrogen) and denatured at 70°C for 10 min. Proteins (35 µg) were separated by SDS-PAGE using NuPAGE 4-12% Bis-Tris gels (Invitrogen). The proteins were transferred to PVDF membranes for 1 h at 100 V. After blocking with 5% milk in TBS-Tween 20 (for PKL detection) or 5% BSA in TBS-Tween20 (for β -actin detection), the membranes were incubated overnight at 4°C with the primary antibodies: rabbit polyclonal anti-PKL diluted 1/1000 in TBS-Tween 20, mouse monoclonal anti-NanoLuc antibodies (Promega) diluted 1/500 in TBS-Tween 20 and mouse monoclonal anti-β-actin antibodies (Abcam, ab8226, 1:1000 diluted in 3% BSA/ TBS-Tween), followed by secondary antibodies donkey anti-rabbit HRP (Cytiva, NA934V) and sheep-anti-mouse-HRP (Cytiva, NA931V), respectively. The incubation with β-actin antibodies was done after incubation with PKL or Nluc antibodies, respectively, and after stripping of the membrane for 10 min with restore plus western blot stripping buffer. The chemiluminescent signal was developed with a ChemiDoc imager (Bio-Rad) after incubation with HRP substrate (Supersignal west femto maximum sensitivity substrate for PKL detection or Immobilon Forte western-HRP substrate, Millipore, WBLUF05000, for β-actin detection).

## *Live-cell NanoBRET experiments with HEK293 cells*

HEK293 cells were passed one day before transfection. The concentration of cells was adjusted to 2x10^5^ cells/ml in Opti-MEM™ without phenol red (Invitrogen) supplemented with 1% FBS (Hyclone). Cells (4x10^6^) were reversed transfected with 1 µg of PKL_Nluc_ plasmid or 1 µg Nluc plasmid and 9 µg of carrier DNA (Promega) using 30 µL of Fugene HD transfection reagent (Promega). Cells were plated (100 µL cell suspension/well) on 96 wells plates (white opaque bottom, Corning 3917) and incubated for 24-25 h. Work solutions (100x) of tracer **IV** were prepared from DMSO stocks and further diluted to 20x concentrations with Tracer buffer (Promega). Twenty-five hours after transfection, the cells were treated with 5 µL/well of each of 20x concentrations of the tracer (final concentrations in the assay were in the range 0.001-1 µM, depending on the experiment). Thereafter, 10 µL of 10x solutions of competitors (diluted in Opti-MEM™ medium from stocks of 100x prepared in DMSO) were added to some sets of cells, while the other set received only Opti-MEM™ containing 1% DMSO. The cells were incubated for 2 h at 37°C (5% CO_2_). After equilibration of plates for 10 min at rt, a solution consisting of the intracellular NanoLuc substrate (Promega intracellular Intracellular TE Nano-Glo® Substrate) and the extracellular NanoLuc Inhibitor (Promega, N2162) was added, and the luminescence was recorded after 5 min of substrate addition using a Spectramax iD5 plate reader (Molecular Devices) at 450 and 550 nm with 1000 ms integration time. For each sample well, BRET ratios were calculated using the following formula:

$$BRET ratio \left( \mathrm{mBU} \right)= \frac{\mathrm{Acceptor}_{S}}{\mathrm{Donor}_{S}}-\frac{\mathrm{Acceptor}_{\mathrm{NT}}}{\mathrm{Donor}_{\mathrm{NT}}}$$

Where Acceptor_S_ is the luminescence intensity of the sample well at 550 nm, Donor_S_ is the luminescence of the sample well at 450 nm, and Acceptor_NT_ and Donor_NT_ are the luminescence of the no-tracer control at 550 nm and 450 nm respectively. Titration data was analyzed using Origin(Pro) 2023^[8]^ and fitted to the Hill equation in Origin to determine EC₅₀ values.

## *Fluorescence indication competition assays*

Competitor working solutions (2x) were prepared by serial dilution (0–40 µM, 4:1 dilution, 12 concentrations, 1% DMSO) from DMSO stock solutions into Tris-HCl buffer (pH 7.4; 50 mM Tris, 10 mM MgCl₂, 100 mM KCl, 0.05% Tween). A combined 2x PKL-tracer **IV** solution was prepared in Tris-HCl buffer containing 1% DMSO, with final concentrations of 0.2 µM PKL and 0.1 µM tracer **IV**. Equal volumes (10 µL) of competitor and PKL-tracer solutions were mixed (1:1) in triplicate into 384-well microplates (Corning Black PS, 3820) and incubated at room temperature for 1 hour. Fluorescence intensity was measured using a SpectraMax iD5 microplate reader (Molecular Devices) with excitation at 450 nm and emission at 550 nm (integration time: 400 ms; read height: 1.0 mm). Titration data were analyzed in Origin(Pro) 2023^[8]^ and fitted to the Hill equation to determine EC₅₀ values.

# **LC-MS Chromatograms**

Chromatograms show purities and identities of compounds **I-IV** with the following configuration for each compound: Top panel – UV trace (254 nm), Middle trace – extracted mass of [M+1]^+^ ion, bottom trace – raw TIC.

Compound **I**


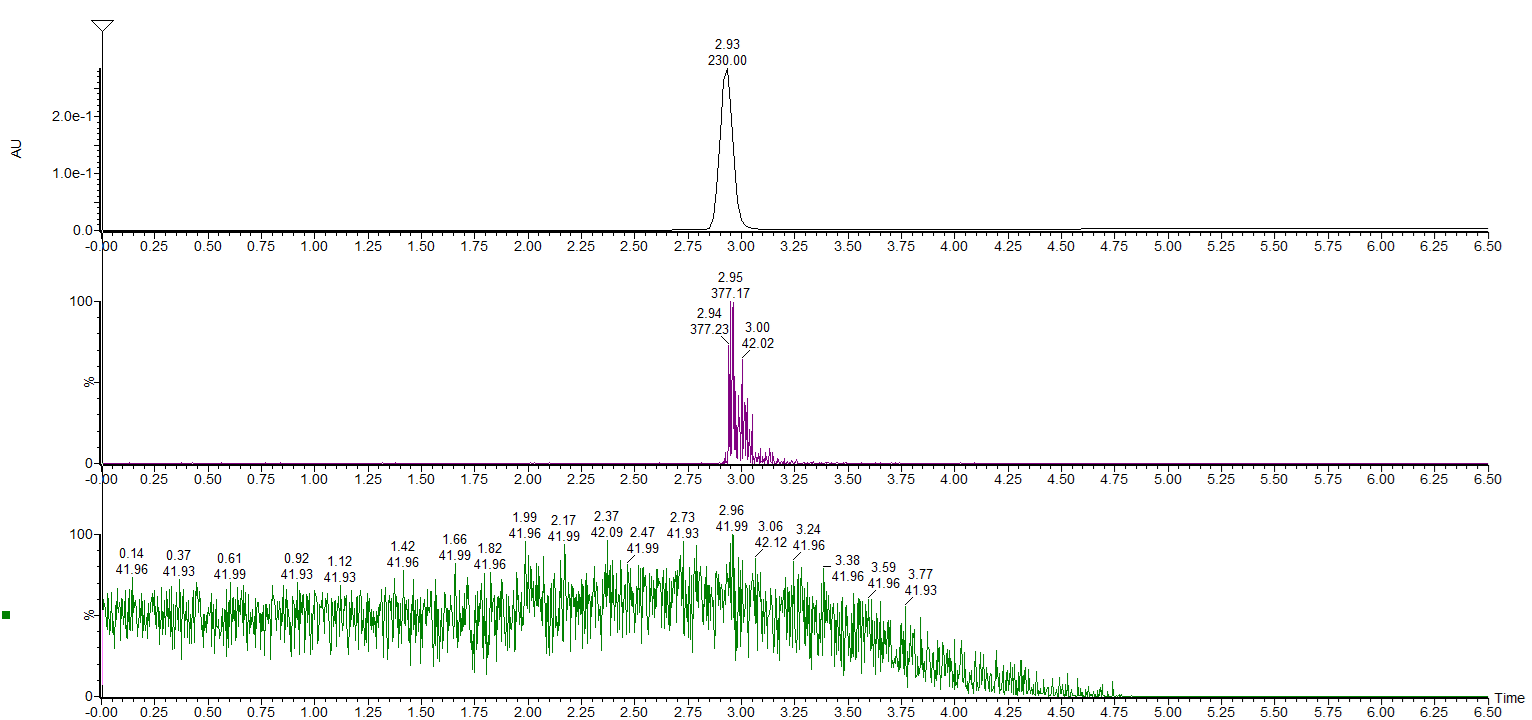


Compound **II**


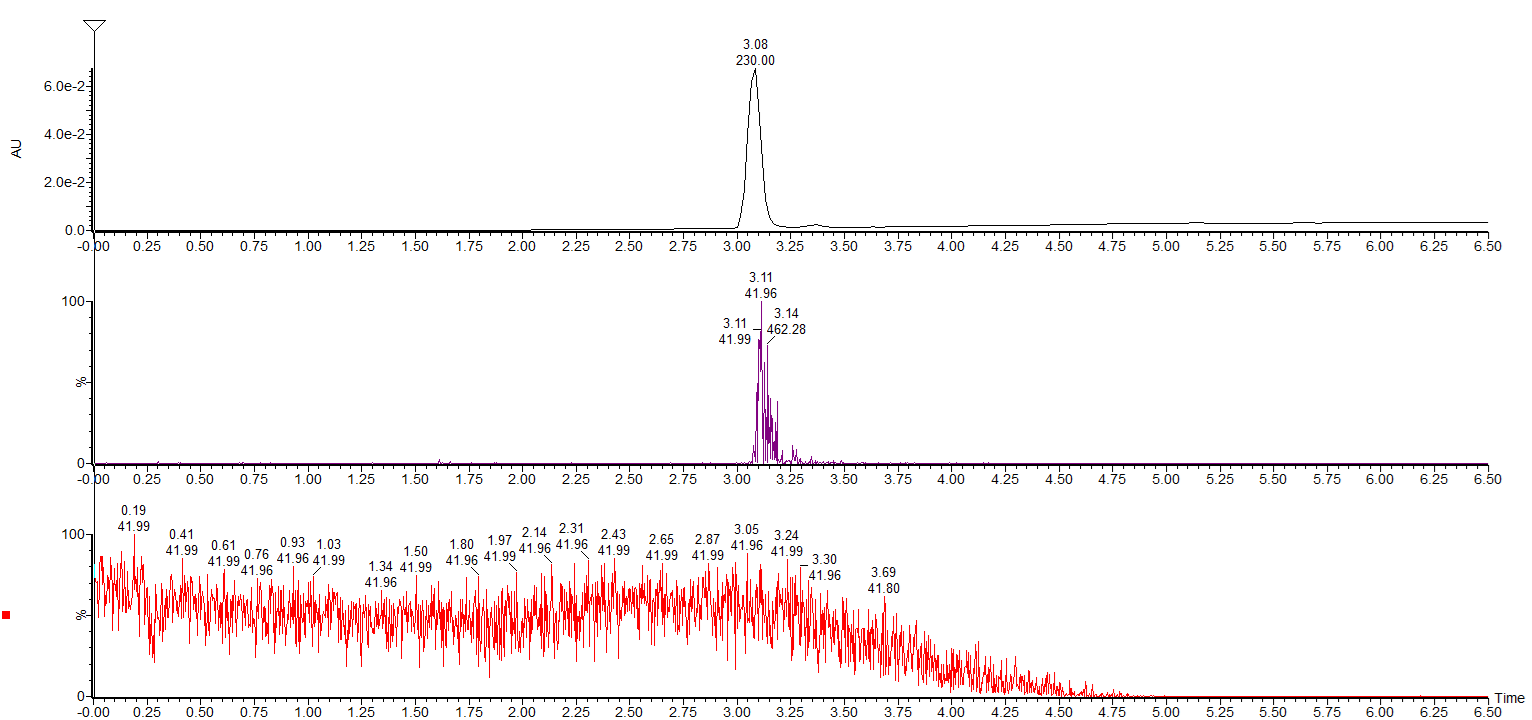


Compound **III**


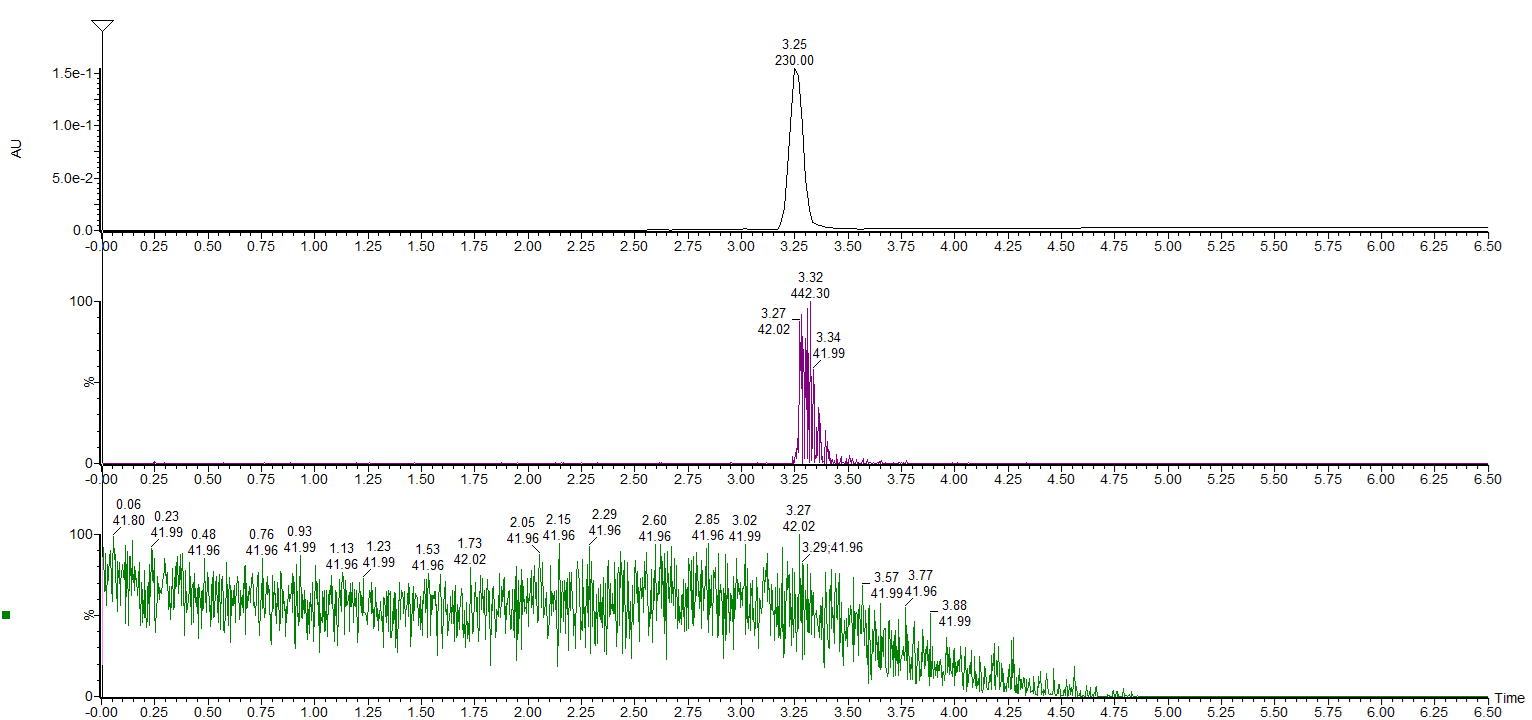


Compound **IV**


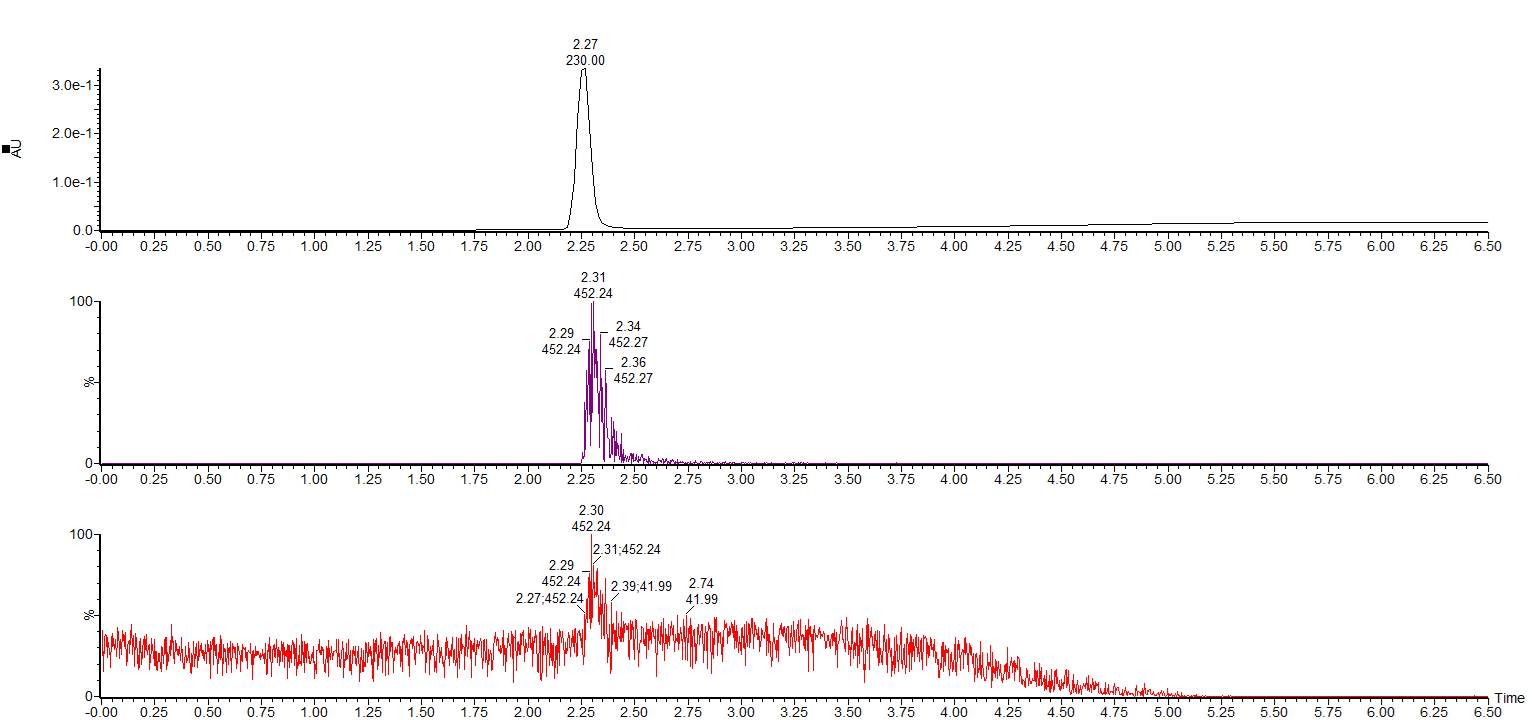


# **NMR Spectra**

Compound **1**

Compound **2**

Compound **3**

Compound **4**

Compound **5**

Compound **6a**

Compound **6b**

Compound **6c**

Compound **7a**

Compound **7b**

Compound **7c**

Compound **7d**

Compound **8a**

Compound **I**

Compound **II**

Compound **III**

Compound **IV**

# **References**

[1] Z. Yang, C. Yan, Y. Chen, C. Zhu, C. Zhang, X. Dong, W. Yang, Z. Guo, Y. Lu, W. He, *Dalton Trans* **2011**, *40*, 2173-2176.

[2] S. Goutelle, M. Maurin, F. Rougier, X. Barbaut, L. Bourguignon, M. Ducher, P. Maire, *Fundam. Clin. Pharmacol.* **2008**, *22*, 633-648.

[3] U. M. Battisti, L. Monjas, F. Akladios, J. Matic, E. Andresen, C. Nagel, M. Hagkvist, L. Håversen, W. Kim, M. Uhlen, J. Borén, A. Mardinoğlu, M. Grøtli, *Pharmaceuticals* **2023**, *16*, 669.

[4] C. Kung, J. Hixon, P. A. Kosinski, G. Cianchetta, G. Histen, Y. Chen, C. Hill, S. Gross, Y. Si, K. Johnson, B. DeLaBarre, Z. Luo, Z. Gu, G. Yao, H. Tang, C. Fang, Y. Xu, X. Lv, S. Biller, S. M. Su, H. Yang, J. Popovici-Muller, F. Salituro, L. Silverman, L. Dang, *Blood* **2017**, *130*, 1347-1356.

[5] S. R. Hoare, *Assay Guidance Manual [Internet]* **2021**.

[6] E. C. Hulme, M. A. Trevethick, *Br. J. Pharmacol.* **2010**, *161*, 1219-1237.

[7] A. Nain-Perez, O. Nilsson, A. Lulla, L. Haversen, P. Brear, S. Liljenberg, M. Hyvönen, J. Borén, M. Grøtli, *Eur. J. Med. Chem.* **2023**, *250*, 115-177.

[8] Origin(Pro), Version 2023b ed., OriginLab Corporation,, Northampton, MA, USA., **2023**.

[9] A. M. Brouwer, *Pure and Applied Chemistry* **2011**, *83*, 2213-2228.

[10] C. A. Schneider, W. S. Rasband, K. W. Eliceiri, *Nature methods* **2012**, *9*, 671-675.

[11] J. Abramson, J. Adler, J. Dunger, R. Evans, T. Green, A. Pritzel, O. Ronneberger, L. Willmore, A. J. Ballard, J. Bambrick, S. W. Bodenstein, D. A. Evans, C. C. Hung, M. O'Neill, D. Reiman, K. Tunyasuvunakool, Z. Wu, A. Zemgulyte, E. Arvaniti, C. Beattie, O. Bertolli, A. Bridgland, A. Cherepanov, M. Congreve, A. I. Cowen-Rivers, A. Cowie, M. Figurnov, F. B. Fuchs, H. Gladman, R. Jain, Y. A. Khan, C. M. R. Low, K. Perlin, A. Potapenko, P. Savy, S. Singh, A. Stecula, A. Thillaisundaram, C. Tong, S. Yakneen, E. D. Zhong, M. Zielinski, A. Zidek, V. Bapst, P. Kohli, M. Jaderberg, D. Hassabis, J. M. Jumper, *Nature* **2024**, *630*, 493-500.

[12] P. Dimitrijevs, M. Makrecka-Kuka, A. Bogucka, M. Hyvonen, T. Pantelejevs, P. Arsenyan, *Eur J Med Chem* **2023**, *257*, 115504.
